# Impact of community masking on COVID-19: A cluster-randomized trial in Bangladesh

**DOI:** 10.1126/science.abi9069

**Published:** 2022-01-14

**Authors:** Jason Abaluck, Laura H. Kwong, Ashley Styczynski, Ashraful Haque, Md. Alamgir Kabir, Ellen Bates-Jefferys, Emily Crawford, Jade Benjamin-Chung, Shabib Raihan, Shadman Rahman, Salim Benhachmi, Neeti Zaman Bintee, Peter J. Winch, Maqsud Hossain, Hasan Mahmud Reza, Abdullah All Jaber, Shawkee Gulshan Momen, Aura Rahman, Faika Laz Banti, Tahrima Saiha Huq, Stephen P. Luby, Ahmed Mushfiq Mobarak

**Affiliations:** 1 Yale School of Management, Yale University, New Haven, CT, USA.; 2 Woods Institute for the Environment, Stanford University, Stanford, CA, USA.; 3 Division of Environmental Health Sciences, University of California, Berkeley, Berkeley, CA, USA.; 4 Division of Infectious Diseases and Geographic Medicine, Stanford University, Stanford, CA, USA.; 5 Innovations for Poverty Action Bangladesh, Dhaka, Bangladesh.; 6 Innovations for Poverty Action, Evanston, IL, USA.; 7 Department of Epidemiology and Population Health, School of Medicine, Stanford University, Stanford, CA, USA.; 8 Yale Research Initiative on Innovation and Scale, Yale University, New Haven, CT, USA.; 9 Social and Behavioral Interventions Program, Johns Hopkins Bloomberg School of Public Health, Baltimore, MD, USA.; 10 NGRI, North South University, Dhaka, Bangladesh.; 11 Department of Pharmaceutical Sciences, North South University, Dhaka, Bangladesh.; 12 Department of Economics, Deakin University, Melbourne, Australia.

## Abstract

We conducted a cluster-randomized trial to measure the effect of community-level mask distribution and promotion on symptomatic severe acute respiratory syndrome coronavirus 2 (SARS-CoV-2) infections in rural Bangladesh from November 2020 to April 2021 (*N* = 600 villages, *N* = 342,183 adults). We cross-randomized mask type (cloth versus surgical) and promotion strategies at the village and household level. Proper mask-wearing increased from 13.3% in the control group to 42.3% in the intervention arm (adjusted percentage point difference = 0.29; 95% confidence interval = [0.26, 0.31]). The intervention reduced symptomatic seroprevalence (adjusted prevalence ratio = 0.91 [0.82, 1.00]), especially among adults ≥60 years old in villages where surgical masks were distributed (adjusted prevalence ratio = 0.65 [0.45, 0.85]). Mask distribution with promotion was a scalable and effective method to reduce symptomatic SARS-CoV-2 infections.

## Persuading people to mask

Even in places where it is obligatory, people tend to optimistically overstate their compliance for mask wearing. How then can we persuade more of the population at large to act for the greater good? Abaluck *et al*. undertook a large, cluster-randomized trial in Bangladesh involving hundreds of thousands of people (although mostly men) over a 2-month period. Colored masks of various construction were handed out free of charge, accompanied by a range of mask-wearing promotional activities inspired by marketing research. Using a grassroots network of volunteers to help conduct the study and gather data, the authors discovered that mask wearing averaged 13.3% in villages where no interventions took place but increased to 42.3% in villages where in-person interventions were introduced. Villages where in-person reinforcement of mask wearing occurred also showed a reduction in reporting COVID-like illness, particularly in high-risk individuals. —CA

## Abstract (structured)

**INTRODUCTION:**

Mask usage remains low across many parts of the world during the COVID-19 pandemic, and strategies to increase mask-wearing remain untested. Our objectives were to identify strategies that can persistently increase mask-wearing and assess the impact of increasing mask-wearing on symptomatic severe acute respiratory syndrome coronavirus 2 (SARS-CoV-2) infections.

**RATIONALE:**

We conducted a cluster-randomized trial of community-level mask promotion in rural Bangladesh from November 2020 to April 2021 (*N* = 600 villages, *N* = 342,183 adults). We cross-randomized mask promotion strategies at the village and household level, including cloth versus surgical masks. All intervention arms received free masks, information on the importance of masking, role modeling by community leaders, and in-person reminders for 8 weeks. The control group did not receive any interventions. Participants and surveillance staff were not informed of treatment assignments, but project materials were clearly visible. Outcomes included symptomatic SARS-CoV-2 seroprevalence (primary) and prevalence of proper mask-wearing, physical distancing, social distancing, and symptoms consistent with COVID-19 illness (secondary). Mask-wearing and distancing were assessed through direct observation at least weekly at mosques, markets, the main entrance roads to villages, and tea stalls. Individuals were coded as physically distanced if they were at least one arm’s length from the nearest adult; social distancing was measured using the total number of adults observed in public areas. At 5- and 9-week follow-ups, we surveyed all reachable participants about COVID-19–related symptoms. Blood samples collected at 10- to 12-week follow-ups for symptomatic individuals were analyzed for SARS-CoV-2 immunoglobulin G (IgG) antibodies.

**RESULTS:**

There were 178,322 individuals in the intervention group and 163,861 individuals in the control group. The intervention increased proper mask-wearing from 13.3% in control villages (*N* = 806,547 observations) to 42.3% in treatment villages (*N* = 797,715 observations) (adjusted percentage point difference = 0.29; 95% confidence interval = [0.26, 0.31]). This tripling of mask usage was sustained during the intervention period and for 2 weeks after. Physical distancing increased from 24.1% in control villages to 29.2% in treatment villages (adjusted percentage point difference = 0.05 [0.04, 0.06]). We saw no change in social distancing. After 5 months, the impact of the intervention on mask-wearing waned, but mask-wearing remained 10 percentage points higher in the intervention group. Beyond the core intervention of free distribution and promotion at households, mosques, and markets; leader endorsements; and periodic monitoring and reminders, several elements had no additional effect on mask-wearing, including text reminders, public signage commitments, monetary or nonmonetary incentives, and altruistic messaging or verbal commitments.

The proportion of individuals with COVID-19–like symptoms was 7.63% (*N* = 12,784) in the intervention arm and 8.60% (*N* = 13,287) in the control arm, an estimated 11.6% reduction after controlling for baseline covariates. Blood samples were collected from consenting, symptomatic adults (*N* = 10,790). Adjusting for baseline covariates, the intervention reduced symptomatic seroprevalence by 9.5% (adjusted prevalence ratio = 0.91 [0.82, 1.00]; control prevalence = 0.76%; treatment prevalence = 0.68%). We find that surgical masks are particularly effective in reducing symptomatic seroprevalence of SARS-CoV-2. In villages randomized to surgical masks (*N* = 200), the relative reduction was 11.1% overall (adjusted prevalence ratio = 0.89 [0.78, 1.00]). The effect of the intervention is most concentrated among the elderly population; in surgical mask villages, we observe a 35.3% reduction in symptomatic seroprevalence among individuals ≥60 years old (adjusted prevalence ratio = 0.65 [0.45, 0.85]). We see larger reductions in symptoms and symptomatic seropositivity in villages that experienced larger increases in mask use. No adverse events were reported.

**CONCLUSION:**

A randomized-trial of community-level mask promotion in rural Bangladesh during the COVID-19 pandemic shows that the intervention increased mask usage and reduced symptomatic SARS-CoV-2 infections, demonstrating that promoting community mask-wearing can improve public health.

## Graphical abstract

**Figure:**
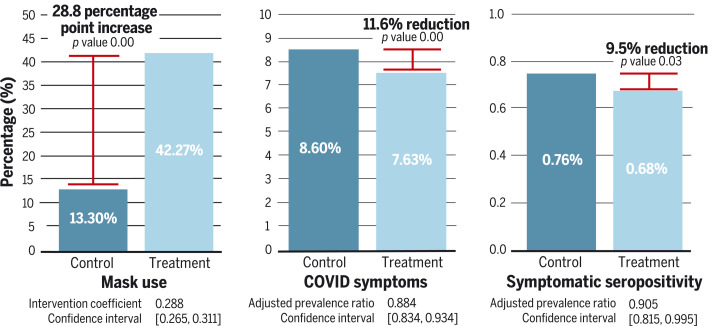
Impact of intervention on mask use and biological outcomes. The figure shows the raw means of mask-wearing (left), COVID-19 symptoms (middle), and symptomatic seropositivity (right) in the control and treatment arms. The estimated change in each outcome, confidence intervals, and *p* values adjust for preregistered covariates (and thus are not computable from the raw values). Individuals who were symptomatic but did not consent to blood collection were dropped from the sample; measured symptomatic seropositivity thus understates the true fraction of the population that was symptomatic seropositive.

As of September 2021, the COVID-19 pandemic has taken the lives of more than 4.7 million people. Inspired by the growing body of scientific evidence that face masks have the potential to slow the spread of the disease and save lives (*1*–*10*), we conducted a cluster-randomized controlled trial covering 342,183 adults in 600 villages in rural Bangladesh with the dual goals of (i) identifying strategies to increase community-wide mask-wearing and (ii) tracking changes in symptomatic severe acute respiratory syndrome coronavirus 2 (SARS-CoV-2) infections as a result of our intervention. Although vaccines may constrain the spread of SARS-CoV-2 in the long-term, it is unlikely that a substantial fraction of the population in low- and middle-income countries will have access to vaccines before the end of 2021 (*11*). Developing scalable and effective means of combating COVID-19 is thus of first-order policy importance.

The World Health Organization (WHO) declined to recommend mask adoption until June 2020, citing the lack of evidence from community-based randomized-controlled trials as well as concerns that mask-wearing would create a false sense of security (*12*). Critics argued that those who wore masks would engage in compensating behaviors, such as failing to physically distance from others, resulting in a net increase in transmission (*13*). We directly test this hypothesis by measuring physical distancing.

We designed our trial to encourage universal mask-wearing at the community level, rather than mask-wearing among only those with symptoms. We encouraged even healthy individuals to wear masks because a substantial share of COVID-19 transmission stems from asymptomatic or presymptomatic individuals (*14*) and masks may protect healthy wearers by reducing the inhalation of aerosols or droplets (*15*–*17*).

After performing pilot studies, we settled on a core intervention package that combined household mask distribution with communication about the value of mask-wearing; mask promotion and in-person reminders at mosques, markets, and other public places; and role-modeling by public officials and community leaders. We also tested several other strategies in subsamples, such as asking people to make a verbal commitment, creating opportunities for social signaling, text messaging, and providing village-level incentives to increase mask-wearing. The selection of strategies to test was informed by both our pilot study results and research in public health, psychology (*18*–*20*), economics (*21*–*23*), marketing (*24*–*26*), and other social sciences (*27*) on product promotion and dissemination strategies. We tested many different strategies because it was difficult to predict in advance which ones would lead to persistent increases in mask-wearing. Prediction studies we conducted with policy-makers and public health experts at the WHO, India’s National Council of Applied Economic Research, and the World Bank suggested that even these experts with influence over policy design could not easily predict which specific strategies would prove most effective in our trial.

We powered our intervention around the primary outcome of symptomatic seroprevalence. During our study, we collected survey data on the prevalence of WHO-defined COVID-19 symptoms from all available study participants and then collected blood samples at endline from those who reported symptoms at any time during the 8-week study. Our trial is therefore designed to track the fraction of individuals who are both symptomatic and seropositive. We chose this as our primary outcome because (i) the goal of public health policy is ultimately to prevent symptomatic infections (even if preventing asymptomatic infections is instrumentally important in achieving that goal) and (ii) symptomatic individuals are far more likely to be seropositive so powering for this outcome required conducting an order of magnitude fewer costly blood tests. As secondary outcomes, we also report the effects of our intervention on WHO-defined symptoms for probable COVID-19 infection and mask-wearing.

Bangladesh is a densely populated country with 165 million inhabitants; reported infections reached 15,000 per day during our study period, but reported cases and deaths are likely underestimated by one to two orders of magnitude (*28*–*32*). The evolution of mask use over time in Bangladesh is discussed in greater detail in (*33*). In Bangladesh, the government strongly recommended mask use from early April 2020. In an April 2020 telephone survey, more than 80% of respondents self-reported wearing a mask and 97% self-reported owning a mask. The Bangladeshi government formally mandated mask use in late May 2020 and threatened to fine those who did not comply, although enforcement was weak to nonexistent, especially in rural areas. During in-person surveillance between 21 and 25 May 2020 in 1441 places in 52 districts, we observed 51% of about 152,000 individuals wearing a mask. Another wave of surveillance was conducted between 19 and 22 June 2020 in the same 1441 locations, and mask-wearing dropped to 26%, with 20% wearing masks that covered their mouth and nose and 6% wearing masks improperly. An August 2020 phone survey in rural Kenya found that although 88% of respondents claim to wear masks in public, direct observation revealed that only 10% actually did (*34*). These observations suggest that mask promotion interventions could be useful in rural areas of low- and middle-income countries, which are home to several billion people at risk for COVID-19.

## Results

Our analysis followed our preregistered analysis plan (https://osf.io/vzdh6/) except where indicated. Our primary outcome was symptomatic seroprevalence for SARS-CoV-2. We also analyzed the impact of our intervention on mask-wearing, physical distancing, social distancing, and COVID-19–like symptoms. No adverse events were reported during the study period.

### Sample selection

**Fig. 1.**
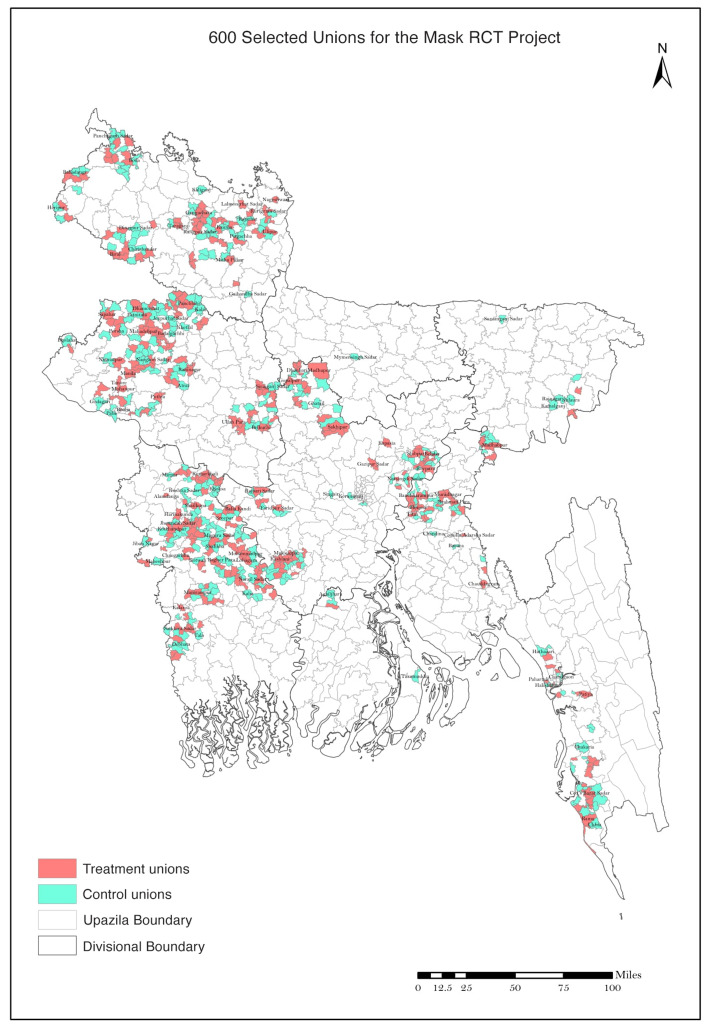
Map of 600 treatment and control unions. The figure shows the location of the 600 treatment and control unions in the study. RCT, randomized controlled trial; 1 mile = 1.6 km.

The unions where we conducted our intervention are geographically dispersed throughout rural Bangladesh, as shown in Fig. 1. (Appendix C discusses in more detail how these unions were selected.) Tables S1 and S2 summarize sample selection for our analysis. We initially approved 134,050 households, of which 125,053 provided baseline information. From these 125,053 households, we collected baseline information from 342,183 individuals. Of these, 336,010 (98%) provided symptom data at week 5 and/or 9. Of these, 27,160 (8.0%) reported COVID-19–like symptoms during the 9 weeks since the study began. We attempted to collect blood samples from all symptomatic individuals. Of these, 10,790 (39.7%) consented to have blood collected (40.2% in the treatment group and 39.3% in the control group; *p* = 0.24). We show in table S3 that consent rates are about 40% across men and women and among adults of different age groups in both treatment and control villages.

As such, the sample of individuals for whom we have symptom data is much larger than the sample for whom we have serology data. We tested 9512 (88.2%) of the collected blood samples to determine seroprevalence for SARS-CoV-2 immunoglobulin G (IgG) antibodies. Untested samples (<12%) either lacked sufficient quantity for our test or could not be matched to individuals from our sample because of a barcode scanning error. In our primary outcome analysis, we drop individuals for whom we are missing symptom data or who did not consent to blood sample collection. For the analyses where symptomatic status is the outcome, we report results using both this smaller sample as well as the larger sample of all individuals who provided symptom data. In the baseline, we collected blood samples from a random sample of individuals (*N* = 10,085), of whom 339 had COVID-19–like symptoms. We use these to check balance with respect to baseline symptomatic seropositivity (as well as baseline symptomatic status).

Of the 600 villages initially recruited for the study, the analysis sample excludes four villages where interventions could not be performed owing to a lack of local government cooperation. We exclude an additional 11 villages and their village-pairs (where a village and its village-pair are a control-treatment pair) because we did not observe them in the baseline period before the intervention and one village and its pair for lack of observational data throughout the intervention period, for a total analysis sample of 572 villages.

### Primary analyses

#### Our primary outcomes are balanced at baseline

Although our stratification procedure should have achieved balance with respect to variables observed at the time of randomization, given the many possible opportunities for errors in implementation, we confirm in appendix L that our control and treatment villages are balanced with respect to our primary outcome variables. This assessment was not preregistered. We investigated several other covariates and found a few small imbalances. We checked whether these affect the main results that we report in this paper. For example, we found more 18- to 30-year-olds in the treatment group than in the control group, perhaps because households reported teenagers as 18 years old to receive more masks; our results are robust to dropping this age range.

#### Our intervention increased mask-wearing

The first column in the top panel of Table 1 reports coefficients from a regression of mask-wearing on a constant, an intervention indicator (based on the assigned groups), baseline mask-wearing, the baseline symptom rate, and indicators for each control-intervention pair. More details of our statistical methods and standard error construction are available in appendix K. Mask-wearing was 13.3% in control villages and 42.3% in treatment villages. Our regression adjusted estimate is an increase of 28.8 percentage points (95% confidence interval = [0.26, 0.31]; numbers in brackets represent 95% confidence intervals throughout the text and tables). If we omit all covariates (except fixed effects for the strata within which we randomized), our point estimate is identical (table S5). Considering only surveillance conducted when no mask distribution was taking place, mask-wearing increased 27.9 percentage points, from 13.4% in control villages to 41.3% in intervention villages (regression adjusted estimate = 0.28 [0.26, 0.30]). We also run our analysis separately in mosques, markets, and other locations such as tea stalls, the entrance of restaurants, and the main road in the village. The increase in mask-wearing was largest in mosques (37.0 percentage points), whereas in all other locations it was 25 to 29 percentage points.

**Table 1.** Mask-wearing and physical distancing, controlling for baseline variables. All regressions include an indicator for each control-intervention pair and baseline symptom rates. The analyses in the top panel control for baseline rates of proper mask-wearing, and the analyses in the bottom panel control for baseline rates of physical distancing. “Baseline symptom rate” is defined as the rate of surveyed individuals in a village who report symptoms coinciding with the WHO definition of a probable COVID-19 case. We assume that (i) all reported symptoms were acute onset, (ii) all people live or work in an area with a high risk of transmission of virus, and (iii) all people have been a contact of a probable or confirmed case of COVID-19 or are linked to a COVID-19 cluster. “No active promotion” refers to any time that surveillance was conducted while promotion was not actively occurring (regardless of the week of the intervention). This excludes surveillance during the Friday Jumma Prayers in the mosque, when promoters were present and actively encouraged mask-wearing. “Other locations” include tea stalls, at the entrance of the restaurant as patrons enter, and the main road to enter the village. “Surgical villages” refer to all treatment villages that received surgical masks as part of the intervention and their control pairs. “Cloth villages” refer to all treatment villages that received cloth masks as part of the intervention and their control pairs. The surgical and cloth subsamples include surveillance from all available locations, equivalent to the column labeled “Full” but run separately for each subgroup. Of the 572 villages included in the analysis sample, we exclude an additional village and its pair in the mosque and market subsamples and two villages and their pairs in the other location subsample because we did not observe them in the baseline period before the intervention. There are 190 treatment villages that received surgical masks as part of the intervention and 96 treatment villages that received cloth masks. Standard errors are in parentheses.

**Parameter**	**Full**	**No active** **promotion**	**Mosques**	**Markets**	**Other** **locations**	**Surgical mask** **villages**	**Cloth mask** **villages**
*Proper mask-wearing*							
Intervention coefficient	0.288*** (0.012)	0.279*** (0.011)	0.370*** (0.016)	0.287*** (0.012)	0.251*** (0.012)	0.301*** (0.015)	0.256*** (0.019)
*Physical distancing*							
Intervention coefficient	0.051*** (0.005)	0.056*** (0.005)	0.000 (0.000)	0.074*** (0.007)	0.068*** (0.006)	0.054*** (0.006)	0.044*** (0.011)
*N* villages	572	572	570	570	568	380	192

***Significant at the 1% level.

**Significant at the 5% level.

*Significant at the 10% level.

#### Our intervention increased physical distancing

Contrary to concerns that mask-wearing would promote risk compensation, we did not find evidence that our intervention undermines distancing behavior. In the bottom panel of Table 1, we report identical specifications to the top panel but with physical distancing as the dependent variable. In control villages, 24.1% of observed individuals practiced physical distancing compared with 29.2% in intervention villages, an increase of 5.1% (regression adjusted estimate = 0.05 [0.04, 0.06]). Evidently, protective behaviors like mask-wearing and physical distancing are complements rather than substitutes: Endorsing mask-wearing and informing people about its importance encouraged rural Bangladeshis to take the pandemic more seriously and engage in another form of self-protection. The increases in physical distancing were similar in cloth and surgical mask villages.

Physical distancing increased 5.1 percentage points overall, but there was substantial heterogeneity across locations. In markets, individuals were 7.4 percentage points more likely to physically distance. By contrast, there was no physical distancing practiced in any mosque, in either treatment or control villages, probably as a result of the strong religious norm of standing shoulder-to-shoulder when praying.

#### Our intervention had no impact on social distancing

It is possible that physical distancing increases because our intervention results in fewer total people being present in public spaces. If socializing increased in the intervention group, but only among risk-conscious people, then we might see physical distancing increase despite people engaging in overall riskier behavior. To assess this, as well as to assess directly if the intervention increased socializing, we studied the effects of our intervention on the total number of people observed at public locations. Although surveillance staff were not able to count everyone in busy public areas, the total number of people they were able to observe gives some indication of the crowd size. We found no difference in the number of people observed in public areas between the treatment and control groups overall (table S6). The social distancing analysis was not preregistered, although the specification exactly parallels our analysis of physical distancing.

#### Our intervention reduced symptomatic seroprevalence

Among the 336,010 participants who completed symptom surveys, 27,160 (8.1%) reported experiencing COVID-19–like illnesses during the study period. More participants in the control villages reported incident COVID-19–like illnesses (*N* = 13,853; 8.6%) compared with participants in the intervention villages (*N* = 13,307; 7.6%). More than one-third (39.7%) of symptomatic participants agreed to blood collection. After omitting symptomatic participants who did not consent to blood collection, symptomatic seroprevalence was 0.76% in control villages and 0.68% in the intervention villages. Because the fractions we are reporting omit nonconsenters from the numerator but not the denominator, it is likely that the true rates of symptomatic seroprevalence are substantially higher (perhaps by 2.5 times, if nonconsenters have similar seroprevalence to consenters).

In Table 2 (and table S7), we report results from a regression of symptomatic seroprevalence on a treatment indicator, clustering at the village level and controlling for fixed effects for each pair of control and treatment villages. In the tables, we report results with and without additional controls for baseline symptoms and mask-wearing rates. In table S7, we report results from our prespecified linear model, and in Table 2, we report results from a generalized linear model with a Poisson family and log-link function. Here, we discuss the latter results (which are in units of relative risk); the linear model implies results of an almost identical magnitude. The prevalence ratios and accompanying confidence intervals reported in the text correspond to the specifications with baseline controls (hence, “adjusted” prevalence ratio).

**Table 2.** Symptomatic seroprevalence, expressed in prevalence ratios. All regressions include an indicator for each control-intervention pair. The regressions “with baseline controls” include controls for baseline rates of proper mask-wearing and baseline symptom rates. “Baseline symptom rate” is defined as the rate of surveyed individuals in a village who report symptoms coinciding with the WHO definition of a probable COVID-19 case. We assume that (i) all reported symptoms were acute onset, (ii) all people live or work in an area with a high risk of transmission of virus, and (iii) all people have been a contact of a probable or confirmed case of COVID-19 or are linked to a COVID-19 cluster. The analysis includes all people surveyed in the baseline household visits, excluding individuals for whom we did not collect midline or endline symptoms, symptomatic individuals from whom we did not collect blood, and individuals from whom we drew blood but did not test their blood. The regressions exclude an additional 17,377 individuals in 34 villages because there are zero people who are symptomatic-seropositive in their village pairs. To check robustness to the type of clustering, in panels 2 and 3 of fig. S2, we show the histogram of effect sizes under “randomization inference” if we randomly reassign treatment within each pair of villages and then estimate our primary specification. We find that our estimated effect size is smaller than 7.0% of the simulated estimates with controls and 7.4% of the simulated estimates without controls (these are the corresponding *p* values of the randomization inference *t* test). Blank spaces indicate variables not included in the regression specification reported in each column.

**Parameter**	**Intervention effect**	**Intervention effect by mask type**
*No baseline controls*		
Intervention prevalence ratio	0.905** [0.815, 0.995]	
Intervention prevalence ratio for surgical mask villages		0.894* [0.782, 1.007]
Intervention prevalence ratio for cloth mask villages		0.925 [0.766, 1.083]
Average symptomatic-seroprevalence rate in paired control villages^†^	0.0076	0.0076
*With baseline controls*		
Intervention prevalence ratio	0.905** [0.815, 0.995]	
Intervention prevalence ratio for surgical mask villages		0.889** [0.780, 0.997]
Intervention prevalence ratio for cloth mask villages		0.942 [0.781, 1.103]
*N* individuals	304,726	304,726
*N* villages	572	572

***Significant at the 1% level.

**Significant at the 5% level.

*Significant at the 10% level.

†We report the mean rate of symptomatic seroprevalence at endline. This is not equivalent to the coefficient on the constant due to the inclusion of the pair indicators as controls.

The results in all specifications are the same: We estimate a roughly 9% decline in symptomatic seroprevalence in the treatment group (adjusted prevalence ratio = 0.91 [0.82, 1.00]) for a 29 percentage point increase in mask-wearing over 8 weeks. In the second column of Table 2 and table S7, we split our results by mask type (surgical versus cloth). We find clear evidence that surgical masks lead to a relative reduction in symptomatic seroprevalence of 11.1% (adjusted prevalence ratio = 0.89 [0.78, 1.00]; control prevalence = 0.81%; treatment prevalence = 0.72%). Although the point estimates for cloth masks suggests that they reduce risk, the confidence limits include both an effect size similar to surgical masks and no effect at all (adjusted prevalence ratio = 0.94 [0.78, 1.10]; control = 0.67%; treatment = 0.61%).

In appendix N, we investigate the robustness of these results to alternative methods of dealing with missing data from nonconsenters. In the main text, following our prespecified analysis plan, we drop nonconsenting symptomatic individuals. If we instead impute seropositivity for symptomatic nonconsenters based on the population average seropositivity among symptomatic individuals, our pooled estimate of the impact of masking becomes larger and more precise. Notably, with this alternative imputation, we find effects for both cloth and surgical masks on symptomatic seroprevalence.

Not all symptomatic seroprevalence is necessarily a result of infections occurring during our intervention; individuals may have had preexisting SARS-CoV-2 infections and then became symptomatic (perhaps caused by an infection other than SARS-CoV-2). In appendix I, we show that if either (i) masks have the same proportional impact on COVID and non-COVID symptoms or (ii) all symptomatic seropositivity is caused by infections during our intervention, then the percentage decline in symptomatic seroprevalence will exactly equal the decline in symptomatic seroconversions. More generally, the relationship between the two quantities depends on whether masks have a greater impact on COVID or non-COVID symptoms, as well as the proportion of symptomatic seropositivity that is a result of infections preexisting at baseline.

#### Our intervention reduced WHO COVID-19 symptoms

In Table 3 and table S8, we report results from the same specifications with WHO-defined COVID-19 symptomatic status as the outcome. This is defined as any of following:

**Table 3.** WHO-defined COVID-19 symptoms, expressed in prevalence ratios. All regressions include an indicator for each control-intervention pair. The regressions “with baseline controls” include controls for baseline rates of proper mask-wearing and baseline symptom rates. “Baseline symptom rate” is defined as the rate of surveyed individuals in a village who report symptoms coinciding with the WHO definition of a probable COVID-19 case. We assume that (i) all reported symptoms were acute onset, (ii) all people live or work in an area with a high risk of transmission of virus, and (iii) all people have been a contact of a probable or confirmed case of COVID-19 or are linked to a COVID-19 cluster. The analysis includes all people surveyed in the baseline household visits, excluding individuals for whom we did not collect midline or endline symptoms. Blank spaces indicate variables not included in the regression specification reported in each column.

**Parameter**	**Intervention effect**	**Intervention effect by mask type**
*No baseline controls*		
Intervention prevalence ratio	0.885*** [0.834, 0.934]	
Intervention prevalence ratio for surgical mask villages		0.865*** [0.803, 0.928]
Intervention prevalence ratio for cloth mask villages		0.922* [0.838, 1.005]
Average symptomatic rate in paired control villages^†^	0.0860	0.0860
*With baseline controls*		
Intervention prevalence ratio	0.884*** [0.834, 0.934]	
Intervention prevalence ratio for surgical mask villages		0.874*** [0.809, 0.939]
Intervention prevalence ratio for cloth mask villages		0.907** [0.823, 0.991]
*N* individuals	321,948	321,948
*N* villages	572	572

***Significant at the 1% level.

**Significant at the 5% level.

*Significant at the 10% level.

†We report the mean rate of symptomatic status at endline. This is not equivalent to the coefficient on the constant due to the inclusion of the pair indicators as controls.

1) Fever and cough.

2) Any three of the following: fever; cough; general weakness and/or fatigue; headache; muscle aches; sore throat; coryza (nasal congestion or runny nose); dyspnoea (shortness of breath or difficulty breathing); anorexia (loss of appetite), nausea, and/or vomiting; diarrhea; or altered mental status.

3) Anosmia (loss of smell) and ageusia (loss of taste).

We find clear evidence that the intervention reduced symptoms: We estimate a reduction of 11.6% (adjusted prevalence ratio = 0.88 [0.83, 0.93]; control = 8.60%; treatment = 7.63%). Additionally, when we look separately by cloth and surgical masks, we find that the intervention led to a reduction in COVID-19–like symptoms under either mask type (*p* = 0.000 for surgical; *p* = 0.066 for cloth), but the effect size in surgical mask villages was 30 to 80% larger depending on the specification. In table S9, we run the same specifications using the smaller sample used in our symptomatic seroprevalence regression (i.e., those who consented to give blood). In this sample, we continue to find an effect overall and an effect for surgical masks but see no statistically significant effect for cloth masks.

### In-person reinforcement is crucial to our intervention

Our core intervention package combined multiple distinct elements: We provided people with free masks and information about the importance of mask-wearing, we had mask promoters reinforce mask-wearing by stopping individuals in public places who were not wearing masks and reminding them to do so, and we partnered with local leaders to encourage mask-wearing at mosques and markets. Additionally, in some villages, we provided a variety of reminders, commitment devices, and incentives for village leaders. In appendix J, we attempt to disentangle the role played by these different elements in encouraging mask use.

We find no evidence that any of our village-level or household-level treatments, other than mask color, affected mask-wearing. For mask color, we see marginally significant differences that are small in magnitude. In surgical mask villages, blue masks were more likely to be observed than green masks (adjusted percentage point difference = 0.03 [−0.00, 0.06]), and in cloth mask villages, red masks were more likely to be observed than purple masks (adjusted percentage point difference = −0.02 [−0.04, −0.00]). Text message reminders, incentives for village-leaders, or explicit commitment signals explain little of the observed increase in mask-wearing. Compared with self-protection messaging alone, altruistic messaging had no greater impact on mask-wearing, and twice-weekly text messages and a verbal commitment had no significant effects. We saw no significant difference in the rates of mask-wearing in the village-level randomization of surgical versus cloth masks.

We do find nonexperimental evidence that in-person mask promotion and reinforcement is a crucial part of our intervention. Our first pilot study contained all elements of our intervention except in-person reinforcement. Our second pilot study (1 week later) and the full intervention (several months later) added in-person reinforcement. Under the assumption that treatment effects would otherwise be constant over time, we find that in-person reinforcement accounts for 19.2 percentage points of our effect (regression adjusted estimate = 0.19 [−0.33, −0.05]), or 65% of the total effect size. In table S10, we show that this difference is statistically significant whether or not we include baseline controls. This was not a prespecified analysis.

### Our intervention yields persistent increases in mask-wearing

In appendix M, we present results on mask-wearing after our intervention ended. Even though the door-to-door free mask distribution occurred in the first week only, there was almost no attenuation of mask-wearing over the initial 10 weeks of surveillance. Notably, mask-wearing remained comparably increased in the treatment group during the 2 weeks we continued surveillance after the end of all intervention activities in the village. Three to 4 months later, mask-wearing waned but remained 10 percentage points higher in treatment regions.

### Subgroup analyses

#### Women wear masks more often, but men respond more to the intervention

In table S11, we analyze the impact of our intervention on mask-wearing and physical distancing separately by gender, as well as by whether baseline mask-wearing was above or below the median. Gender was recorded in 65% of observations; age was not recorded during the direct observation surveillance of mask-wearing in public places, and thus we do not conduct an age-stratified assessment. This observed sample is representative of the rural Bangladeshi population that is present in crowded public places during the day; this population is largely composed of men, who have more social contacts outside the home than women. In the gender results, we drop surveillance observations for mosques because in rural Bangladesh it is rare for women to attend mosque. We found that the intervention increased mask-wearing by 27.1 percentage points for men ([0.25, 0.30]) and 22.5 percentage points for women ([0.20, 0.25]). Although we do not have the variation to test this, the gendered difference in effect size may be because our mask promoters were predominantly men or because the mask-wearing rate in control villages was so much higher for women (31% for women versus 12% for men). We intentionally hired predominantly men because most staff interactions would be with men. Men constituted 88.2% of all observed adults. We also found a larger increase in mask-wearing in villages with below-median baseline mask-wearing (where mask-wearing increased from 8.7 to 41.9% at endline) than in those with above-median baseline mask-wearing (where the increase was from 17.5 to 42.6%).

#### The effect on symptomatic seroprevalence is especially large among the elderly

In Table 4 and table S12, we report results from our primary specification separately by age. Table S12 reports our preregistered specification, a linear model run separately for each decade of age, pooling cloth mask and surgical mask villages. Table 4 synthesizes these results, collapsing by categories of <40, 40 to 49, 50 to 59, and ≥60 years old, reporting results as a relative risk reduction, and showing results separately for surgical and cloth masks. We generally find that the impact of the intervention is concentrated among individuals over age 50. In surgical mask villages, we observe a 22.8% decline in symptomatic seroprevalence among individuals aged 50 to 59 years (adjusted prevalence ratio = 0.77 [0.60, 0.95]) and a 35.3% decline among individuals ≥60 years old in our baseline specification (*p* = 0.000) (adjusted prevalence ratio = 0.65 [0.45, 0.85]). For cloth masks, we find an insignificant (5%) reduction overall but some evidence of a reduction in symptomatic seroprevalence among 40- to 49-year-olds; we investigate more deeply in appendix N and find that the age gradient appears to be sensitive to how we deal with missing values. In the bottom panel of Table 4, we report results where we impute the population average seroprevalence among all nonconsenters rather than dropping them. This alternative approach yields more precise overall estimates and suggests that both cloth and surgical masks have greater impacts on symptomatic seroprevalence at older ages, although the impact of surgical masks among those ≥60 years old is smaller than in our baseline specification. Ex ante, it is not obvious to us which imputation method should be preferred, although the second approach makes our results less sensitive to differential consent rates that we observe in some waves of our intervention, as discussed in appendix N.

**Table 4.** Symptomatic seroprevalence by age groups and mask type, expressed in prevalence ratios. All regressions include an indicator for each control-intervention pair. The regressions include controls for baseline rates of mask-wearing and baseline symptom rates. “Baseline symptom rate” is defined as the rate of surveyed individuals in a village who report symptoms coinciding with the WHO definition of a probable COVID-19 case. We assume that (i) all reported symptoms were acute onset, (ii) all people live or work in an area with a high risk of transmission of virus, and (iii) all people have been a contact of a probable or confirmed case of COVID-19 or are linked to a COVID-19 cluster. The analysis in the top panel uses the preregistered sample, equivalent to that in Table 2; it includes all people surveyed in the baseline household visits, excluding individuals for whom we did not collect midline or endline symptoms, symptomatic individuals from whom we did not collect blood, and individuals from whom we drew blood but did not test their blood. The analysis in the bottom panel replicates the regressions in the top panel but imputes the seropositivity of individuals from whom we did not draw blood. For symptomatic individuals from whom we did not draw blood, we simulate their symptomatic-seroprevalence status by using the average rate of conditional seropositivity among all symptomatic individuals. This analysis includes all people surveyed in the baseline household visits, excluding individuals for whom we did not collect midline or endline symptoms.

**Parameter**	**All**	**<40 years old**	**40 to** **49 years old**	**50 to** **59 years old**	**≥60 years old**
*Preregistered sample: Drop individuals without blood draws*					
Intervention prevalence ratio for surgical mask villages	0.889** [0.780, 0.997]	0.967 [0.834, 1.100]	1.009 [0.817, 1.200]	0.772** [0.595, 0.949]	0.647*** [0.448, 0.845]
Intervention prevalence ratio for cloth mask villages	0.942 [0.781, 1.103]	1.058 [0.870, 1.247]	0.713** [0.459, 0.967]	0.838 [0.524, 1.153]	1.084 [0.769, 1.399]
Average symptomatic-seroprevalence in paired control villages^†^	0.0076	0.0055	0.0095	0.0108	0.0104
*N* individuals	287,349	146,306	35,839	24,086	27,943
*N* villages	538	480	384	348	360
*Imputing symptomatic-seroprevalence for missing blood draws*					
Intervention prevalence ratio for surgical mask villages	0.873*** [0.801, 0.945]	0.917* [0.829, 1.005]	0.975 [0.862, 1.088]	0.815*** [0.688, 0.942]	0.701*** [0.577, 0.824]
Intervention prevalence ratio for cloth mask villages	0.890** [0.787, 0.993]	0.861*** [0.758, 0.965]	0.838** [0.678, 0.998]	1.153 [0.970, 1.336]	0.792** [0.601, 0.983]
Average symptomatic-seroprevalence in paired control villages^†^	0.0189	0.0152	0.0226	0.0229	0.0251
*N* individuals	321,383	177,708	51,676	37,340	43,431
*N* villages	570	566	528	504	534

***Significant at the 1% level.

**Significant at the 5% level.

*Significant at the 10% level.

†We report the mean rate of symptomatic seroprevalence at endline. This is not equivalent to the coefficient on the constant due to the inclusion of the pair indicators as controls.

#### The effect on WHO COVID-19 symptoms is larger among the elderly

In tables S13 and S14 (the latter being our preregistered specification), we perform the same analysis using the larger sample of individuals who reported symptom information. In this sample, we continue to find larger effects at older ages, although the differences are not as stark as those for the symptomatic seroprevalence outcome. In table S15, we show that the age gradient is steeper for surgical masks.

#### Men and women have similar reductions in symptoms and symptomatic seroprevalence

In appendix N and table S28, we show results for symptoms and symptomatic seropositivity by gender. We see a similar pattern to the cloth and surgical mask results: We see significant effects for both genders for symptoms and symptomatic seroposivity when we impute seropositivity at the average value for nonconsenters. If we instead drop nonconsenters, the symptomatic seropositivity estimates for men become less precise and are no longer significantly different from zero, whereas the estimates for women remain unchanged.

#### Additional preregistered specifications

In appendix P, we discuss additional preregistered specifications that are not reported in the text, either because they were substantially underpowered given the available data or because data on required variables were unavailable. We also discuss ways in which trial implementation deviated from our preregistered protocol, such as switching from exclusively phone surveys to household visits at weeks 5 and 9 to increase response rates.

### Intervention cost and benefit estimates

In appendix Q, we assess the costs of implementing our intervention relative to the health benefits, specifically focusing on our ongoing efforts to implement this same intervention at scale in Bangladesh. We consider a range of possible estimates for excess deaths from COVID-19 from 1 May to 1 September 2021, and we assume that our age-specific impacts on symptomatic seroprevalence will lead to proportional reductions in mortality. We estimate that a scaled version of our intervention being implemented in Bangladesh will cost about $1.50 per person, and between $10,000 and $52,000 per life saved, depending on which estimate we use for excess deaths.

## Discussion

We present results from a cluster-randomized controlled trial of a scalable intervention designed to increase mask-wearing and reduce COVID-19 symptomatic infections. Our estimates suggest that mask-wearing increased by 28.8 percentage points, corresponding to an estimated 51,357 additional adults wearing masks in intervention villages, and this effect was persistent even after active mask promotion was discontinued. The intervention led to a 9.5% reduction in symptomatic SARS-CoV-2 seroprevalence (which corresponds to 105 fewer symptomatic seropositives) and an 11.6% reduction in the prevalence of COVID-19–like symptoms, corresponding to 1541 fewer people reporting these symptoms. If we assume that nonconsenting symptomatic individuals were seropositive at the same rate as consenting symptomatic individuals, the total estimated symptomatic seropositives prevented would be 354. The effects were substantially larger (and more precisely estimated) in communities where we distributed surgical masks, consistent with their greater filtration efficiency as measured in the laboratory (manuscript forthcoming). In villages randomized to receive surgical masks, the relative reduction in symptomatic seroprevalence was 11% overall, 23% among individuals aged 50 to 59 years, and 35% among those ≥60 years of age in preferred specifications.

We found clear evidence that surgical masks are effective in reducing symptomatic seroprevalence of SARS-CoV-2. Although cloth masks clearly reduce symptoms, we find less clear evidence of their impact on symptomatic SARS-CoV-2 infections, with the statistical significance depending on whether we impute missing values for nonconsenting adults. The number of cloth mask villages (100) was half that for surgical masks (200), meaning that our results tend to be less precise. Additionally, we found evidence that surgical masks were no less likely to be adopted than cloth masks. Surgical masks have higher filtration efficiency, are cheaper, are consistently worn, and are better supported by our evidence as tools to reduce COVID-19 cases.

Our results should not be taken to imply that mask-wearing can prevent only 10% of COVID-19 cases, let alone 10% of COVID-19 mortality. Our intervention induced 29 more people out of every 100 to wear masks, with 42% of people wearing masks in total. The total impact with near-universal masking—perhaps achievable with alternative strategies or stricter enforcement—may be several times larger than our 10% estimate. Additionally, the intervention reduced symptomatic seroprevalence more when surgical masks were used and even more for the highest-risk individuals in our sample (23% for ages 50 to 59 years and 35% for ages ≥60 years). These numbers likely give a better sense of the impact of our intervention on severe morbidity and mortality, because most of the disease burden of the COVID-19 pandemic is borne by the elderly. Where achievable, universal mask adoption is likely to have still larger impacts.

There are several possible theories for why we might observe a larger reduction in COVID-19 cases for older adults. We did not directly measure age during surveillance, but mask-wearing could have increased more for older adults. A second theory is that older adults are more susceptible to infections at viral loads that are preventable by masks. A third theory is that older adults have fewer social connections, so that reducing transmission through any one connection is more likely to prevent infection by severing all transmissible routes. A fourth theory is that people exercised more care and were more likely to wear masks when proximate to the elderly.

We identified a combination of core intervention elements that were effective in increasing mask-wearing in rural Bangladesh: Mask distribution and role-modeling, combined with mask promotion, lead to large and sustained increases in mask use. Results from our pilot studies suggest that combining mask distribution, role-modeling, and active mask promotion—rather than mask distribution and role-modeling alone—seems critical to achieving the full effect. Our trial results also highlight many factors that appear inessential: We find no evidence that public commitments, village-level incentives, text messages, altruistic messaging, or verbal commitments change mask-wearing behavior. The null results on our cross-randomizations do not necessarily imply that these approaches are not worth trying in other contexts, but they teach us that large, persistent increases in mask-wearing are possible without these elements.

Prediction studies that we conducted with policy-makers and public health experts at the WHO and the World Bank before presentations of the study results suggest that our results are informative for policy design. Most of the respondents in the prediction studies anticipated that text messages, verbal commitments, and incentives would increase mask-wearing, when in reality, we estimated fairly precise null effects, and poll respondents believed that in-person mask promotion would have no additional effect, whereas the evidence from our pilot studies suggests that it is essential (for additional details, see appendix R).

Our intervention design is immediately relevant for Bangladesh’s plans for larger-scale distribution of masks across all rural areas. The Bangladesh Directorate General of Health has assigned the study team and the nongovernmental organization Bangladesh Rural Advancement Committee (BRAC) the responsibility to scale up the strategies that were proven most effective in this trial to reach 81 million people (*35*). At the time of writing, we are implementing this program in the 37 districts prioritized by the government based on SARS-CoV-2 test positivity rates. Our results are also relevant for mask dissemination and promotion campaigns planned in other countries and settings that face similar challenges in ensuring mask usage as a result of limited reach and enforcement capacity. The mask promotion model described in this paper was subsequently adopted by governments and other implementers in Pakistan (*36*), India (*37*), and Nepal (*38*). The intervention package would be feasible to implement in a similar fashion in other world regions as well. Beyond face masks, the conceptual underpinning of our strategies could be applied to encourage the adoption of other health behaviors and technologies, in particular, those easily observable by others outside the household, such as purchase and consumption of food, alcohol, and tobacco products in stores, restaurants, or other public spaces (*39*); hand washing and infection control in health care facilities (*40*–*42*); hygiene interventions in childcare and school settings (*43*, *44*); improved sanitation (*45*, *46*); or vaccination drives (*47*).

Although critics of mask mandates suggest that individuals who wear masks are more likely to engage in high-risk behaviors (*48*), we found no evidence of risk compensation as a result of increased mask-wearing. Indeed, we found that our intervention slightly increased the likelihood of physical distancing, presumably because individuals participating in the intervention took the threat of COVID-19 more seriously. These findings are consistent with other behaviors, including seat belt use (*49*) or immunization (*50*), where risk compensation, even if present, is not sufficient to outweigh direct effects.

The intervention may have influenced rates of COVID-19 by increasing mask use, physical distancing, and/or other risk prevention behaviors. Three factors suggest that the direct impact of masks is the most likely explanation for our documented health impacts. First, in appendix O, we analyze cross-sectionally the relationship between our biological outcomes and both mask-wearing and physical distancing. We find that symptoms and symptomatic seropositivity are negatively correlated with mask-wearing, but not with physical distancing, after controlling for mask-wearing. This analysis uses variation in observational data, rather than solely experimental data, and should therefore be interpreted with caution, as discussed in the appendix. Second, we see no change in physical distancing in the highest-risk environment in our study, typically crowded indoor mosques. However, women do not typically go to mosques in rural Bangladesh, and their symptomatic seropositivity decreased by just as much as that of men, so outdoor transmission or transmission in settings that we do not observe directly may be important. Third, our study complements a large body of laboratory and quasi-experimental evidence that masks have a direct effect on SARS-CoV-2 transmission (*1*).

We estimate that a scaled version of our intervention being implemented in Bangladesh will cost between $10,000 and $52,000 per life saved, depending on what fraction of excess deaths are attributable to COVID-19. This is considerably lower than the value of a statistical life in Bangladesh [$205,000 (*51*)] and, under severe outbreaks, is comparable to the most cost-efficient humanitarian programs at scale [e.g., distributing insecticide nets to prevent malaria costs $9200 per life saved (*52*)]. This estimate includes only mortality impacts and not morbidity, and greater cost-efficiency is possible if our intervention can be streamlined to further isolate the essential components. Most of our costs were the personnel costs for mask-promoters: If we consider only the costs of mask production, these numbers would be 20 times lower. Thus, the overall cost to save a life in countries where mask mandates can be enforced at minimal cost with existing infrastructure may be substantially lower than our estimates above.

### Study limitations

Our study has several limitations. The distinct appearance of project-associated masks and increased mask-wearing in intervention villages made it impossible to blind surveillance staff to study-arm assignment. However, staff were not informed about the exact purpose of the study. Even though surveillance staff were plain-clothed and were instructed to remain discreet, community members could have recognized that they were being observed and changed their behavior. Additionally, survey respondents could have changed their likelihood of reporting symptoms in places where mask-wearing was more widespread. If respondents were more cognizant of symptoms in mask-wearing areas, this may bias us toward underestimating the impact of masks; if respondents in mask-wearing areas were less concerned with mild symptoms and thus were less likely to recall them, this might bias us toward overestimating the impact of masks. Although we confirm that blood consent rates are not significantly different in the treatment and control groups and are comparable across all demographic groups, we cannot rule out that the composition of consenters differed between the treatment and control groups. The slightly higher point estimate for consent in the treatment group biases us away from finding an effect, because it raises symptomatic seroprevalence in the treatment group. Although control villages were at least 2 km from intervention villages, adults from control villages may have come to intervention villages to receive masks, reducing the apparent impact of the intervention. Although we did not directly assess harms in this study, there could be costs resulting from discomfort with increased mask-wearing, adverse health effects such as dermatitis or headaches, or impaired communication.

Because the study was powered to detect differences in symptomatic seroprevalence, we cannot distinguish whether masks work by making symptoms less severe (through a reduced viral load at transmission) or by reducing new infections. We selected the WHO case definition of COVID-19 for its sensitivity, though its limited specificity may imply that the impact of masks on symptoms comes partly from non–SARS-CoV-2 respiratory infections. If masks reduce COVID-19 by reducing symptoms (for a given number of infections), they could help ease the morbidity and mortality resulting from a given number of SARS-CoV-2 infections. If masks reduce infections, they may reduce the total number of infections over the long-term by buying more time to increase the fraction of the population that is vaccinated. At the time of the study, the predominant circulating SARS-CoV-2 strain was B.1.1.7 (Alpha) (*53*). The impacts of the Delta variant on the number of infections prevented by a given mask-wearer are uncertain; the population-wide consequences of infections prevented by a given mask-wearer may be larger given a higher reproduction number.

We found that mask distribution, role modeling, and promotion in a low- and middle-income country setting increased mask-wearing and physical distancing, leading to lower illness, particularly in older adults. We find especially robust evidence that surgical masks prevent COVID-19. Whether people with respiratory symptoms should generally wear masks to prevent respiratory virus transmission, including for viruses other than SARS-CoV-2, is an important area for future research. Our findings suggest that such behavior may benefit public health.

## Methods and materials

### Sampling frame and timeline

The intervention protocol, prespecified analysis plan, and CONSORT checklist are available at https://osf.io/vzdh6/. We discuss our sample-size calculations in appendix B and discuss the selection and pairwise randomization in appendix C. In brief, we stratified villages based on geographic location and available case data, and then selected one treatment and one control village from each pair.

Village-level cluster randomization was important for three reasons. First, unlike technologies with primarily private benefits, mask adoption is likely to yield especially large benefits at the community level. Second, mask adoption by some may influence mask adoption by others because mask-wearing is immediately visible to other members of the community (*45*). Third, this design allows us to assess the full impact of masks on symptomatic infections, including through source control. Individual-level randomization would identify only whether masks protect wearers.

Our intervention was designed to last 8 weeks in each village. The intervention started in different villages at different times, rolling out over a 6-week period in seven waves. There were between 16 and 61 village-pairs grouped in each wave based on geographic proximity, and paired control and treatment villages were always included in the same wave. The first wave was rolled out on 17 and 18 November 2020 and the last wave was rolled out on 5 and 6 January 2021.

Innovations for Poverty Action (IPA) staff traveled to many villages that had low mask uptake in the first 5 weeks of the study and found that in these villages, local leaders were not very engaged in supporting mask promotion. Hence, we retrained mask-promotion staff partway through the intervention to work more closely with local leaders and set specific milestones for that partnership.

### Outcomes

Our primary outcome was symptomatic seroprevalence of SARS-CoV-2. Our secondary outcomes were prevalence of proper mask-wearing, physical distancing, and symptoms consistent with COVID-19. For COVID-19 symptoms, we used the symptoms that correspond to the WHO case definition of probable COVID-19 given epidemiological risk factors: (i) fever and cough; (ii) three or more of the following symptoms (fever; cough; general weakness and/or fatigue; headache; myalgia; sore throat; coryza; dyspnea; anorexia, nausea, and/or vomiting; diarrhea; and altered mental status); or (iii) loss of taste or smell. Seropositivity was defined by having detectable IgG antibodies against SARS-CoV-2.

### Intervention materials and activities

Our entire intervention was designed to be easily adopted by other nongovernmental organizations or government agencies and required minimal monitoring. We have made the materials public in multiple languages to ease widespread adoption and replication by other implementers (https://osf.io/23mws/).

We provide design specifications for our masks in appendix F. We used high-quality surgical masks that had a filtration efficiency of 95% [standard deviation (SD) = 1%]; this is substantially higher than the filtration efficiency of the cloth masks we designed, which had a filtration efficiency of 37% (SD = 6%). These cloth masks had substantially higher filtration than common commercial three-ply cotton masks but lower filtration than hybrid masks that use materials not commonly available for community members in low-resource settings (*54*). Although cloth masks have less leakage because they fit the face more closely (*55*) and can be sewn without specialized equipment, they are an order of magnitude more expensive than surgical masks. The filtration efficiency of the high-quality surgical masks used in this study was 76% after washing them with bar soap and water 10 times (manuscript forthcoming). Although surgical masks can break down into microplastics that can enter the environment if disposed of improperly, an analysis of waste generated in Bangladesh’s first lockdown finds that the mass of surgical mask waste was one-third that of polyethylene bags, which also break down into macro- and microplastics (*56*–*58*).

Surgical masks were outfitted with a sticker that had a logo of a mask with an outline of the Bangladeshi flag and a phrase in Bengali that noted that the mask could be washed and reused (*59*). The relatively large scale of our bulk order allowed us to negotiate mask prices of $0.50 per cloth mask and $0.13 per surgical mask ($0.06 of which was the cost of a sticker reminding people that they could wash and reuse the surgical mask).

Adult household members were asked to wear masks whenever they were outside their house and around other people. To emphasize the importance of mask-wearing, we prepared a brief video of notable public figures discussing why, how, and when to wear a mask. The video was shown to each household during the mask distribution visit and featured the Honorable Prime Minister of Bangladesh Sheikh Hasina, the head of the Imam Training Academy, and the national cricket star Shakib Al Hasan. During the distribution visit, households also received a brochure based on WHO materials that depicted proper mask-wearing.

We implemented a basic set of interventions in all treatment villages and cross-randomized additional intervention elements in randomly chosen subsets of treatment villages to investigate whether those have any additional impact on mask-wearing. The basic intervention package consists of five main elements:

1) One-time mask distribution and information provision (about masks) at households.

2) Mask distribution in markets for 3 to 6 days per week during all 8 weeks of the intervention.

3) Mask distribution at mosques on three Fridays during the first 4 weeks of the intervention.

4) Mask promotion in public spaces and markets where non–mask wearers were encouraged to wear masks (weekly or biweekly).

5) Role modeling and advocacy by local leaders, including imams discussing the importance of mask-wearing at Friday prayers using a scripted speech provided by the research team.

Participants and mask surveillance staff were not told which villages were in which intervention arm, but the intervention materials were clearly visible. The prespecified analyses and sample exclusions were made by analysts blinded to the treatment assignment.

### Cross-randomization of behavior change communication and incentives

#### Village-level cross-randomizations

Within the intervention arm, we cross-randomized villages to four village-level and four household-level treatments to test the impact of a range of social and behavior change communication strategies on mask-wearing. All intervention villages were assigned to either the treatment or the control group of each of these four randomizations. These village-level randomizations were as follows:

1) Randomization of treated villages to either cloth or surgical masks.

2. Randomization of treated villages to public commitment (providing households signage and asking them to place signage on doors that declares they are a mask-wearing household) or not. The signage was meant to encourage formation of social norms through public signaling.

3. Randomization of treated villages to no incentive, nonmonetary incentive, or monetary incentive of $190 given to the village leader for a project benefitting the public. We announced that the monetary reward or the certificate would be awarded if village-level mask-wearing among adults exceeded 75% at 8 weeks after the intervention started.

4. Randomization of treated villages to 0 or 100% of households receiving twice-weekly text message reminders about the importance of mask-wearing.

#### Household-level cross-randomizations

We had three household-level cross-randomizations. In any single village, only one of these household randomizations was operative. Because our data collection protocols relied on passive observation at the village level, we could not record the mask-wearing behavior of individual households. To infer the effect of the household-level treatments, we therefore varied the color of the masks distributed to the household based on its cross-randomization status and had surveillance staff record the mask color of observed individuals. In surgical mask villages, a household received blue or green masks and promoters distributed an equal number of blue and green masks in public settings. In cloth mask villages, households received violet or red masks and promoters distributed blue masks in public settings. To avoid conflating the effect of the household-specific treatment with the effect of the mask color, we randomized which color corresponded to which treatment status across villages (this way a specific color was not fully coincident with a specific treatment). The household-level randomizations, described in further detail in appendix D and visualized in fig. S1, were as follows:

1) Households were randomized to receive messages emphasizing either altruism or self-protection.

2) Households were randomized to making a verbal commitment to be a mask-wearing household (all adults in the household promise to wear a mask when they are outside and around other people) or not. This experiment was conducted in a third set of villages where there was no public signage commitment.

3) Households were randomized to receive twice-weekly text reminders or not. As mentioned above, the text message saturation was randomly varied to 0, 50, or 100% of all households receiving texts, and in the 50% villages, the specific households that received the texts was also random.

#### Conceptual basis for tested social and behavioral change communication

We selected intervention elements that had a reasonable chance of persuading rural Bangladeshis to wear masks by consulting literature in public health, development and behavioral economics, and marketing to identify some of the most promising strategies. An extensive literature identifies price and access as key deterrents to the adoption of welfare-improving products, and especially of technologies that produce positive health externalities, such as face masks (*21*, *60*). Household distribution of free face masks therefore formed the core part of our strategy. Inspired by large literature in marketing and economics on the role of opinion leaders in new product diffusion, we additionally emphasized a partnership with community leaders in mask distribution (*25*, *61*).

The additional village- and household-level treatments we experimented with were also motivated by insights from marketing, public health, development, and behavioral economics. For example, masks are a visible good where social norms are expected to be important, so we consulted the literature that documented peer effects in product adoption (*62*–*65*). We experimented with incentives because it is unclear whether extrinsic rewards crowd out intrinsic motivation (*66*–*68*). We tested whether soft commitment devices encourage targets to follow through with actual behavior change (*69*, *70*), whether public displays can promote social norms (*27*), whether an altruistic framing inspires people more or less than self-interest (*71*), whether social image concerns and signaling can lead to higher compliance (*22*, *72*), and whether regular reminders are a useful tool to ensure adoption (*23*).

### Piloting interventions

IPA implemented two pilot studies: Pilot 1 from 22 to 31 July 2020 and Pilot 2 from 13 to 26 August 2020. The objective of the pilot studies was to mimic some of the major aspects of the main experiment to identify implementation challenges. Each pilot study was conducted in 10 unions that were not part of the main study area. We used the difference between the pilot studies to better understand which elements of our full intervention were essential. We also conducted focus group discussions and in-depth interviews with village residents, community leaders, religious leaders, and political leaders to elicit opinions on how to maximize the effectiveness of the intervention.

### Surveillance strategies

Mask-wearing and physical distancing were measured through direct observation. Surveillance was conducted using a standard protocol that instructed staff to spend 1 hour at each of the following high-traffic locations in the village: market, restaurant entrances, main road, tea stalls, and mosque; the location and timing changed so that the mask-wearing and physical distancing practices of as many individuals as possible could be recorded. Although SARS-CoV-2 transmission is more likely in indoor locations with limited ventilation than outside, rural Bangladeshi villages have few nonresidential spaces where people gather, so observations were conducted outside except at the mosque, where surveillance was conducted inside.

Surveillance staff were distinct from intervention implementation staff and conducted surveillance in paired intervention and control villages. To minimize the likelihood that village residents would perceive that their mask-wearing behavior was being observed, surveillance staff were separate from mask promoters and wore no identifying apparel while passively observing mask-wearing and physical distancing practices in the communities. They recorded the mask-wearing behavior of all of the adults that they were able to observe during surveillance periods; observations were not limited to adults from enrolled households. Surveillance staff noted whether adults were wearing any mask or face covering, whether the mask was one distributed by our project (and, if so, the color), and how the mask was worn. We defined proper mask-wearing as wearing either a project mask or an alternative face-covering over the mouth and nose and improper mask-wearing as wearing a mask in any way that did not fully cover the mouth and nose. Surveillance staff observed a single individual and recorded that person as practicing physical distancing if he or she was at least one arm’s length away from all other people. Additional details are available in appendix G.

### Symptomatic SARS-CoV-2 testing

#### Symptom reporting

The owner of the household’s primary phone completed surveys by phone or in-person at weeks 5 and 9 after the start of the intervention. They were asked to report symptoms experienced by any household member that occurred in the previous week and over the previous month. COVID-19–like symptoms were defined by whether they were consistent with the WHO COVID-19 case definition for suspected or probable cases with an epidemiological link (*73*).

#### Blood sample collection

We collected endline capillary blood samples from participants who reported COVID-19–like symptoms during the study period and consented to blood collection. We additionally collected samples on a subset of randomly selected participants at baseline, independent of symptoms, to assess overall seropositivity. For the purposes of blood collection, endline was defined as 10 to 12 weeks from the start of the intervention. Blood samples were obtained by puncture with a 20-gauge safety lancet to the third or fourth digit. Five hundred microliters of blood were collected into Microtainer capillary blood collection serum separator tubes (BD, Franklin Lakes, NJ). Blood samples were transported on ice and stored at −20°C until testing.

#### SARS-CoV-2 testing

Blood samples were tested for the presence of IgG antibodies against SARS-CoV-2 using the SCoV-2 Detect IgG ELISA kit (InBios, Seattle, WA). This assay detects IgG antibodies against the spike protein subunit (S1) of SARS-CoV-2. The assays were performed according to the manufacturer’s instructions. Additional details are presented in appendix H.

### Symptomatic seropositivity

Our primary outcome is symptomatic seropositivity. As noted above, individuals are symptomatic if they (i) meet the WHO surveillance definition of probable COVID-19 illness and (ii) are seropositive in our blood test at endline. If either of these conditions fail to hold, $Y_{ij}=0$, where $Y_{ij}$ is an indicator for whether individual *i* in village *j* is symptomatic seropositive. To assess seropositivity, we tested all individuals who were symptomatic in either our 5- or 9-week household survey.

Our goal is to estimate the impact of the intervention on symptomatic seropositivity, defined as $\psi_{0}=E_{x}[E(Y_{ij}|T_{j}=1,x_{j})-E(Y_{ij}|T_{j}=0,x_{j})]$ where $T_{j}$ is an indicator for whether a village was treated and $x_{j}$ are village-level covariates, including baseline mask use in each village (constructed as described below) and baseline influenza-like illness and COVID-19 illness based on reported symptoms, as well as indicators for each pair of villages from our pairwise stratification method.

In our preregistered specification, we estimate this parameter by ordinary least squares, clustering at the village level using the approach in (*74*–*76*). The dependent variable is $Y_{ij}$, the independent variable of interest is $T_{j}$, and controls are included for the $x_{j}$ covariates, including baseline mask use and baseline respiratory symptom rates in each village. We also report results from a generalized linear model with a Poisson family and log-link function to compute relative risk (*77*). More details of our statistical analyses are reported in appendix K.

## Supplementary Material

20211202-1

## Supplementary Materials

### This PDF file includes:

Figs. S1 to S6 Tables S1 to S37 Appendices References (*79*–*81*)

### Other Supplementary Material for this manuscript includes the following:

MDAR Reproducibility Checklist
