# Supplementary material for: Impact of community masking on COVID-19: A cluster-randomized trial in Bangladesh
Source: Science. 2022 Jan 14;375(6577):eabi9069. doi: 10.1126/science.abi9069 (PMC9036942; doi:10.1126/science.abi9069)
Supplement: 20211202-1 [file science.abi9069.v1.pdf]

Cite as: J. Abaluck *et al*., *Science* 10.1126/science.abi9069 (2021).

Impact of community masking on COVID-19: A cluster-randomized trial in Bangladesh

Jason Abaluck[1]†*, Laura H. Kwong[2,3]†, Ashley Styczynski[4]†, Ashraful Haque[5], Md. Alamgir Kabir[5], Ellen Bates-Jefferys[6], Emily Crawford[1], Jade Benjamin-Chung[7], Shabib Raihan[5], Shadman Rahman[5], Salim Benhachmi[8], Neeti Zaman Bintee[5], Peter J. Winch[9], Maqsud Hossain[10], Hasan Mahmud Reza[11], Abdullah All Jaber[10], Shawkee Gulshan Momen[10], Aura Rahman[10], Faika Laz Banti[10], Tahrima Saiha Huq[10], Stephen P. Luby[2,4]‡, Ahmed Mushfiq Mobarak[1,12]‡*

[1]Yale School of Management, Yale University, New Haven, CT, USA. [2]Woods Institute for the Environment, Stanford University, Stanford, CA, USA. [3]Division of Environmental Health Sciences, University of California, Berkeley, Berkeley, CA, USA. [4]Division of Infectious Diseases and Geographic Medicine, Stanford University, Stanford, CA, USA. [5]Innovations for Poverty Action Bangladesh, Dhaka, Bangladesh. [6]Innovations for Poverty Action, Evanston, IL, USA. [7]Department of Epidemiology and Population Health, School of Medicine, Stanford University, Stanford, CA, USA. [8]Yale Research Initiative on Innovation and Scale, Yale University, New Haven, CT, USA. [9]Social and Behavioral Interventions Program, Johns Hopkins Bloomberg School of Public Health, Baltimore, MD, USA. [10]NGRI, North South University, Dhaka, Bangladesh. [11]Department of Pharmaceutical Sciences, North South University, Dhaka, Bangladesh. [12]Department of Economics, Deakin University, Melbourne, Australia.

†These authors contributed equally to this work. ‡These authors contributed equally to this work. *Corresponding author. Email: jason.abaluck@yale.edu (J.A.); ahmed.mobarak@yale.edu (A.M.M.)

We conducted a cluster-randomized trial to measure the effect of community-level mask distribution and promotion on symptomatic SARS-CoV-2 infections in rural Bangladesh from November 2020 to April 2021 (N = 600 villages, N = 342,183 adults). We cross-randomized mask type (cloth vs. surgical) and promotion strategies at the village and household level. Proper mask-wearing increased from 13.3% in the control group to 42.3% in the intervention arm (adjusted percentage point difference = 0.29 [0.26, 0.31]). The intervention reduced symptomatic seroprevalence (adjusted prevalence ratio = 0.91 [0.82, 1.00]), especially among adults 60+ years in villages where surgical masks were distributed (adjusted prevalence ratio = 0.65 [0.45, 0.85]). Mask distribution and promotion was a scalable and effective method to reduce symptomatic SARS-CoV-2 infections.

As of September 2021, the COVID-19 pandemic has taken the lives of more than 4.7 million people. Inspired by the growing body of scientific evidence that face masks have the potential to slow the spread of the disease and save lives (*1*–*10*), we conducted a cluster-randomized controlled trial covering 342,183 adults in 600 villages in rural Bangladesh with the dual goals of (a) identifying strategies to increase community-wide mask-wearing, and (b) tracking changes in symptomatic SARS-CoV-2 infections as a result of our intervention. While vaccines may constrain the spread of SARS-CoV-2 in the long-term, it is unlikely that a substantial fraction of the population in low- and middle-income countries will have access to vaccines before the end of 2021 (*11*). Developing scalable and effective means of combating COVID-19 is thus of first-order policy importance.

The World Health Organization declined to recommend mask adoption until June 2020, citing the lack of evidence from community-based randomized-controlled trials, as well as concerns that mask-wearing would create a false sense of security (*12*). Critics argued those who wore masks would engage in compensating behaviors, such as failing to physically distance from others, resulting in a net increase in transmission (*13*). We directly test this hypothesis by measuring physical distancing.

We designed our trial to encourage *universal* mask-wearing at the community level, rather than mask-wearing among only those with symptoms. We encouraged even healthy individuals to wear masks since a substantial share of COVID-19 transmission stems from asymptomatic or pre-symptomatic individuals (*14*), and masks may protect healthy wearers by reducing the inhalation of aerosols or droplets (*15*–*17*).

After piloting, we settled on a core intervention package that combined household mask distribution with communication about the value of mask-wearing, mask promotion and in-person reminders at mosques, markets, and other public places, and role-modeling by public officials and community leaders. We also tested several other strategies in sub-samples, such as asking people to make a verbal commitment, creating opportunities for social signaling, text messages, and providing village-level incentives to increase mask-wearing. The selection of strategies to test was informed by both our pilot results and research in public health, psychology (*18*–*20*), economics (*21*–*23*), marketing (*24*–*26*), and other social sciences (*27*) on product promotion and dissemination strategies. We tested many different strategies because it was

difficult to predict in advance which ones would lead to persistent increases in mask-wearing. Prediction studies we conducted with policymakers and public health experts at the World Health Organization, India's National Council of Applied Economic Research, and the World Bank suggest that even these experts with influence over policy design could not easily predict which specific strategies would prove most effective in our trial.

We powered our intervention around the primary outcome of symptomatic seroprevalence. During our study, we collected survey data on the prevalence of WHO-defined COVID-19 symptoms from all available study participants, and then collected blood samples at endline from those who reported symptoms anytime during the 8-week study. Our trial is therefore designed to track the fraction of individuals who are *both* symptomatic and seropositive. We chose this as our primary outcome because (a) the goal of public health policy is ultimately to prevent symptomatic infections (even if preventing asymptomatic infections is instrumentally important in achieving that goal) and (b) symptomatic individuals are far more likely to be seropositive so powering for this outcome required conducting an order of magnitude fewer costly blood tests. As secondary outcomes, we also report the effects of our intervention on WHO-defined symptoms for probable COVID-19 and mask-wearing.

Bangladesh is a densely populated country with 165 million inhabitants; reported infections reached 15,000 per day in during our study period, but reported cases and deaths are likely underestimated by 1-2 orders of magnitude (*28*–*32*). The evolution of mask use over time in Bangladesh is discussed in greater detail in (*33*). In Bangladesh, the government strongly recommended mask use from early April 2020. In an April 2020 telephone survey, over 80% of respondents self-reported wearing a mask and 97% self-reported owning a mask. The Bangladeshi government formally mandated mask use in late May 2020 and threatened to fine those who did not comply, although enforcement was weak to non-existent, especially in rural areas. During in-person surveillance between May 21-25, 2020 in 1,441 places in 52 districts, we observed 51% of approximately 152,000 individuals wearing a mask. In another wave of surveillance was conducted between June 19-22, 2020 in the same 1,441 locations, and mask-wearing dropped to 26%, with 20% wearing masks that covered their mouth and nose and 6% wearing masks improperly. An August 2020 phone survey in rural Kenya found that while 88% of respondents claim to wear masks in public, direct observation revealed that only 10% actually did (*34*). These observations suggest that mask promotion interventions could be useful in rural areas of low- and middle-income countries (LMIC), home to several billion people at risk for COVID-19.

Results

Our analysis followed our preregistered analysis plan (https://osf.io/vzdh6/) except where indicated. Our primary outcome was symptomatic seroprevalence for SARS-CoV-2. We also analyzed the impact of our intervention on mask-wearing, physical distancing, social distancing, and COVID-like symptoms. No adverse events were reported during the study period.

Sample selection

The unions where we conducted our intervention are geographically dispersed throughout rural Bangladesh as shown in fig. S3 (appendix C discusses in more detail how these unions were selected). Tables S1 and S2 summarize sample selection for our analysis. We initially approved 134,050 households, of which 125,053 provided baseline information. From these 125,049 households, we collected baseline information from 342,183 individuals. Of these, 336,010 (98%) provided symptom data at week 5 and/or 9. Of these, 27,160 (8.0%) reported COVID-like symptoms during the 9-weeks since the study began. We attempted to collect blood samples from all symptomatic individuals. Of these, 10,790 (39.7%) consented to have blood collected (40.2% in the treatment group and 39.3% in the control group; p = 0.24). We show in Table S3 that consent rates are about 40% across men and women and among adults of different age groups in both treatment and control villages.

As such, the sample of individuals for whom we have symptom data is much larger than the sample for whom we have serology data. We tested 9,512 (88.2%) of the collected blood samples to determine seroprevalence for SARS-CoV-2 IgG antibodies. Untested samples (<12%) either lacked sufficient quantity for our test or could not be matched to individuals from our sample because of a barcode scanning error. In our primary outcome analysis, we drop individuals for whom we are missing symptom data or who did not consent to blood sample collection. For the analyses where symptomatic status is the outcome, we report results using both this smaller sample, as well as the larger sample of all individuals who provided symptom data. In the baseline, we collected blood samples from a random sample of individuals (n = 10,085), of whom 339 had COVID-like symptoms. We use these to check balance with respect to baseline symptomatic seropositivity (as well as baseline symptomatic status).

Of the 600 villages initially recruited for the study, the analysis sample excludes 4 villages where interventions could not be performed due to lack of local government cooperation. We exclude an additional 11 villages and their village-pairs because we did not observe them in the baseline period prior to the intervention, and 1 village and its pair for lack of observational data throughout the intervention period, for a total analysis sample of 572 villages.

Primary analyses

Our primary outcomes are balanced at baseline

While our stratification procedure should have achieved balance with respect to variables observed at the time of randomization, given the many possible opportunities for errors in implementation, we confirm in appendix L that our control and treatment villages are balanced with respect to our primary outcome variables. This assessment was not preregistered. We investigate several other covariates and find a few small imbalances. We check whether these affect the main results we report in this paper. For example, we find more 18-30 year olds in the treatment group than in control, perhaps because households reported teenagers as 18 in order to receive more masks; our results are robust to dropping this age range.

Our intervention increased mask-wearing

The first column in the top panel of Table 1 reports coefficients from a regression of mask-wearing on a constant, an intervention indicator (based on the assigned groups), baseline mask-wearing, the baseline symptom rate, and indicators for each control-intervention pair. More details of our statistical methods and standard error construction are available in appendix K. Mask-wearing was 13.3% in control villages and 42.3% in treatment villages. Our regression adjusted estimate is an increase of 28.8 percentage points (95% CI: 0.26, 0.31). If we omit all covariates (except fixed effects for the strata within which we randomized), our point-estimate is identical (table S5). Considering only surveillance conducted when no mask distribution was taking place, mask-wearing increased 27.9 percentage points, from 13.4% in control villages to 41.3% in intervention villages (regression adjusted estimate: 0.28, 95% CI: 0.26, 0.30). We also run our analysis separately in mosques, markets, and other locations such as tea stalls, the entrance of restaurants, and the main road in the village. The increase in mask-wearing was largest in mosques (37.0 percentage points), while in all other locations it was 25-29 percentage points.

Our intervention increased physical distancing

Contrary to concerns that mask-wearing would promote risk compensation, we did not find evidence that our intervention undermines distancing behavior. In the second panel of Table 1, we report identical specifications to the first panel, but with physical distancing as the dependent variable. In control villages 24.1% of observed individuals practiced physical distancing compared to 29.2% in intervention villages, an increase of 5.1% (a regression adjusted estimate of 0.05 [95% CI: 0.04,0.06]). Evidently, protective behaviors like mask-wearing and physical-distancing are complements rather than substitutes: endorsing mask-wearing and informing people about its importance encouraged rural Bangladeshis to take the pandemic more seriously and engage in another form of self-protection. The increases in physical distancing were similar in cloth and surgical mask villages.

Physical distancing increased 5.1 percentage points overall but there was substantial heterogeneity across locations. In markets, individuals become 7.4 percentage points more likely to physically distance. In contrast, there was no physical distancing practiced in any mosque, in either treatment or control villages, probably as a result of the strong religious norm of standing shoulder-to-shoulder when praying.

Our intervention had no impact on social distancing

It is possible that physical distancing increases because our intervention results in fewer total people being present in public spaces. If socializing increased in the intervention group, but only among risk-conscious people, then we might see physical distancing increase despite people engaging in overall riskier behavior. To assess this, as well as to assess directly if the intervention increased socializing, we study the effects of our intervention on the total number of people observed at public locations. While surveillance staff were not able to count everyone in busy public areas, the total number of people they were able to observe gives some indication of the crowd size. We find no difference in the number of people observed in public areas between the treatment and control groups overall (table S6). The social distancing analysis was not pre-registered, although the specification exactly parallels our analysis of physical distancing.

Our intervention reduced symptomatic seroprevalence

Among the 336,010 participants who completed symptom surveys, 27,160 (8.1%) reported experiencing COVID-like illnesses during the study period. More participants in the control villages reported incident COVID-like illnesses (n=13,853, 8.6%) compared with participants in the intervention villages (n=13,307, 7.6%). Over one-third (39.7%) of symptomatic participants agreed to blood collection. Omitting symptomatic participants who did not consent to blood collection, symptomatic seroprevalence was 0.76% in control villages and 0.68% in the intervention villages. Because the fractions we are reporting omit non-consenters from the numerator but not the denominator, it is likely that the true rates of symptomatic seroprevalence are substantially higher (perhaps by 2.5 times, if non-consenters have similar seroprevalence to consenters).

In Table 2 (and table S7), we report results from a regression of symptomatic seroprevalence on a treatment indicator, clustering at the village level and controlling for fixed effects for each pair of control-treatment villages. In the tables, we report results with and without additional controls for baseline symptoms and mask-wearing rates. In table S7, we report results from our pre-specified linear model and in Table 2 we report results from a generalized linear model with a Poisson family and log-link function. Here we discuss the latter

results (which are in units of relative risk); the linear model implies results of an almost identical magnitude. The prevalence ratios and accompanying confidence intervals reported in text correspond to the specifications with baseline controls (hence, “adjusted” prevalence ratio).

The results in all specifications are the same: we estimate a roughly 9% decline in symptomatic seroprevalence in the treatment group {adjusted prevalence ratio (aPR) = 0.91 [0.82, 1.00]} for a 29 percentage point increase in mask wearing over 8 weeks. In the second column of Table 2 and table S7, we split our results by mask type (surgical vs. cloth). We find clear evidence that surgical masks lead to a relative reduction in symptomatic seroprevalence of 11.1% (aPR = 0.89 [0.78,1.00]; control prevalence = 0.81%; treatment prevalence = 0.72%). Although the point estimates for cloth masks suggests that they reduce risk, the confidence limits include both an effect size similar to surgical masks and no effect at all. (aPR = 0.94 [0.78,1.10]; control: 0.67%; treatment: 0.61%).

In appendix N, we investigate the robustness of these results to alternative methods of dealing with missing data from non-consenters. In the main text, following our prespecified analysis plan, we drop non-consenting symptomatic individuals. If we instead impute seropositivity for symptomatic non-consenters based on the population average seropositivity among symptomatic individuals, our pooled estimate of the impact of masking becomes larger and more precise. Notably, with this alternative imputation, we find effects for both cloth and surgical masks on symptomatic seroprevalence.

Not all symptomatic seroprevalence is necessarily a result of infections occurring during our intervention; individuals may have pre-existing SARS-CoV-2 infections and then become symptomatic (perhaps caused by an infection other than SARS-CoV-2). In appendix I, we show that if either: a) masks have the same proportional impact on COVID and non-COVID symptoms or b) all symptomatic seropositivity is caused by infections during our intervention, then the percentage decline in symptomatic seroprevalence will exactly equal the decline in symptomatic seroconversions. More generally, the relationship between the two quantities depends on whether masks have a greater impact on COVID or non-COVID symptoms, as well as the proportion of symptomatic seropositivity that is a result of infections pre-existing at baseline.

Our intervention reduced WHO COVID-19 symptoms

In Table 3 and table S8, we report results from the same specifications with WHO-defined COVID-19 symptomatic status as the outcome. This is defined as any of following:

- Fever and Cough;
- Any three of the following: fever, cough, general weakness/fatigue, headache, muscle aches, sore throat, coryza [nasal congestion or runny nose], dyspnoea [shortness of breath or difficulty breathing], anorexia [loss of appetite]/nausea/vomiting, diarrhea, altered mental status;
- Anosmia [loss of smell] and ageusia [loss of taste].

We find clear evidence that the intervention reduced symptoms: we estimate a reduction of 11.6% (aPR = 0.88 [0.83,0.93]; control: 8.60%; treatment: = 7.63%). Additionally, when we look separately by cloth and surgical masks, we find that the intervention led to a reduction in COVID-like symptoms under either mask type (p = 0.000 for surgical, p = 0.066 for cloth), but the effect size in surgical mask villages was 30-80% larger depending on the specification. In table S9, we run the same specifications using the smaller sample used in our symptomatic seroprevalence regression (i.e., those who consented to give blood). In this sample we continue to find an effect overall and an effect for surgical masks, but see no statistically significant effect for cloth masks.

In-person reinforcement is crucial to our intervention

Our core intervention package combined multiple distinct elements: we provided people with free masks and information about the importance of mask-wearing; we had mask promoters reinforce by stopping individuals in public places who were not wearing masks and reminding them, and we partnered with local leaders to encourage mask-wearing at mosques and markets. Additionally, in some villages we provided a variety of reminders, commitment devices, and incentives for village leaders. In appendix J, we attempt to disentangle the role played by these different elements in encouraging mask use.

We find no evidence that any of our village-level or household-level treatments, other than mask color, impacted mask-wearing. For mask-color, we see marginally significant differences, small in magnitude. In surgical mask villages, blue masks were more likely to be observed than green (adjusted percentage point difference = 0.03, [-0.00,0.06]), and in cloth mask villages, red more likely than purple (adjusted percentage point difference = -0.02, [-0.04,-0.00]). Text message reminders, incentives for village-leaders, or explicit commitment signals explain little of the observed increase in mask-wearing. Compared to self-protection messaging alone, altruistic messaging had no greater impact on mask-wearing, and twice-weekly text messages and a verbal commitment had no significant effects. We saw no significant difference in the rates of mask-wearing in the village-level randomization of surgical vs. cloth masks.

We do find non-experimental evidence that in-person mask promotion and reinforcement is a crucial part of our intervention. Our first pilot contained all elements of our intervention except in-person reinforcement. Our second pilot (one week later) and the full intervention (several months later) added in-person reinforcement. Under the assumption

that treatment effects would otherwise be constant over time, we find that in-person reinforcement accounts for 19.2 percentage points of our effect (regression adjusted estimate 0.19 [-0.33,-0.05]), or 65% of the total effect size. In table S10, we show that this difference is statistically significant whether or not we include baseline controls. This was not a pre-specified analysis.

Our intervention yields persistent increases in mask-wearing

In appendix M, we present results on mask-wearing after our intervention ended. Even though the door-to-door free mask distribution occurred in the first week only, there was almost no attenuation of mask-wearing over the initial 10 weeks of surveillance. Notably, mask-wearing remained comparably elevated in the treatment group during the two weeks we continued surveillance after the end of all intervention activities in the village. 3-4 months later, mask-wearing waned, but remained 10 percentage point higher in treatment regions.

Subgroup analyses

We also considered how the impact of our intervention differed between subgroups.

Women wear masks more but men respond more to the intervention

In table S11, we analyze the impact of our intervention on mask-wearing and physical distancing separately by gender, as well as by whether baseline mask-wearing was above or below the median. Gender was recorded in 65% of observations; age was not recorded during the direct observation surveillance of mask-wearing in public places, and thus we do not conduct an age-stratified assessment. This observed sample is representative of the rural Bangladeshi population that is present in crowded public places during the day; this population is largely men, who have more social contacts outside the home than women. In the gender results, we drop surveillance observations for mosques because in rural Bangladesh it is rare for women to attend mosque. We found that the intervention increased mask-wearing by 27.1 percentage points for men ([0.25,0.30]) and 22.5 percentage points for women ([0.20,0.25]). Although we do not have the variation to test this, the gendered difference in effect size may be because our mask promoters were predominantly men, or because the mask-wearing rate in control villages was so much higher for women (31% for women vs. 12% for men). We intentionally hired predominantly men because most staff interactions would be with men. Men constituted 88.2% of all observed adults.

We also found a larger increase in mask-wearing in villages with below-median baseline mask-wearing (where mask-wearing increased from 8.7% to 41.9% at endline) than those with above-median baseline mask-wearing (where the increase was from 17.5% to 42.6%).

The effect on symptomatic seroprevalence is especially large among the elderly

In Table 4 and table S12, we report results from our primary specification separately by age. Table S12 reports our preregistered specification, a linear model run separately for each decade of age, pooling cloth and surgical villages. Table 4 synthesizes these results, collapsing by categories of <40, 40-50, 50-60 and 60+, reporting results as a relative risk reduction, and showing results separately for surgical and cloth masks. We generally find that the impact of the intervention is concentrated among individuals over age 50. In surgical mask villages, we observe a 22.8% decline in symptomatic seroprevalence among individuals aged 50-60 (adjusted prevalence ratio of 0.77 [0.60,0.95]) and a 35.3% decline among individuals aged 60+ in our baseline specification (p = 0.000) (adjusted prevalence ratio of 0.65 [0.45, 0.85]). For cloth masks, we find an insignificant (5%) reduction overall, but some evidence of a reduction in symptomatic seroprevalence among 40-49 year olds; we investigate more deeply in appendix N, and find that the age gradient appears to be sensitive to how we deal with missing values. In the second panel of Table 4, we report results where we impute the population average seroprevalence among all non-consenters rather than dropping them. This alternative approach yields more precise overall estimates, and suggests that both cloth and surgical masks have greater impacts on symptomatic seroprevalence at older ages, although the impact of surgical masks among age 60+ is smaller than in our baseline specification. Ex ante, it is not obvious to us which imputation method should be preferred, although the second approach makes our results less sensitive to differential consent rates that we observe in some waves of our intervention, as discussed in appendix N.

The effect on WHO COVID-19 symptoms is larger among the elderly

In tables S13 and S14 (the latter our preregistered specification), we perform the same analysis using the larger sample of individuals who reported symptom information. In this sample, we continue to find larger effects at older ages, although the differences are not as stark as for the symptomatic seroprevalence outcome. In table S15, we show that the age gradient is steeper for surgical masks.

Men and women have similar reductions in symptoms and symptomatic seroprevalence

In appendix N and table S28, we show results for symptoms and symptomatic seropositivity by gender. We see a similar pattern to the cloth and surgical results: we see significant effects for both genders for symptoms and symptomatic

seroposivity when we impute seropositivity at the average value for non-consenters. If we instead drop non-consenters, the symptomatic seropositivity estimates for men become less precise and are no longer significantly different from zero, while the estimates for women remain unchanged.

Additional preregistered specifications

In appendix P, we discuss additional preregistered specifications not reported in the text, either because they were substantially underpowered given the available data or because data on required variables was unavailable. We also discuss ways in which trial implementation deviated from our preregistered protocol, such as switching from exclusively phone surveys to household visits at weeks 5 and 9 in order to increase response rates.

Intervention cost and benefit estimates

In appendix Q, we assess the costs of implementing our intervention relative to the health benefits, specifically focusing on our ongoing efforts to implement this same intervention at scale in Bangladesh. We consider a range of possible estimates for excess deaths from COVID-19 from May 1, 2021 - September 1, 2021, and we assume that our age-specific impacts on symptomatic seroprevalence will lead to proportional to reductions in mortality. We estimate that a scaled version of our intervention being implemented in Bangladesh will cost about $1.50 per person, and between $10K and $52K per life saved, depending which estimate we use for excess deaths.

Discussion

We present results from a cluster-randomized controlled trial of a scalable intervention designed to increase mask-wearing and reduce COVID-19. Our estimates suggest that mask-wearing increased by 28.8 percentage points, corresponding to an estimated 51,357 additional adults wearing masks in intervention villages, and this effect was persistent even after active mask promotion was discontinued. The intervention led to a 9.5% reduction in symptomatic SARS-CoV-2 seroprevalence (which corresponds to a 105 fewer symptomatic seropositives) and an 11.6% reduction in the prevalence of COVID-like symptoms, corresponding to 1,541 fewer people reporting these symptoms. If we assume that non-consenting symptomatic individuals were seropositive at the same rate as consenting symptomatic individuals, the total estimated symptomatic seropositives prevented would be 354. The effects were substantially larger (and more precisely estimated) in communities where we distributed surgical masks, consistent with their greater filtration efficiency measured in the laboratory (manuscript forthcoming). In villages randomized to receive surgical masks, the relative reduction in symptomatic seroprevalence was 11% overall, 23% among individuals aged 50-60, and 35% among those over 60 in preferred specifications.

We found clear evidence that surgical masks are effective in reducing symptomatic seroprevalence of SARS-CoV-2. While cloth masks clearly reduce symptoms, we find less clear evidence of their impact on symptomatic SARS-CoV-2 infections, with the statistical significance depending on whether we impute missing values for non-consenting adults. The number of cloth mask villages (100) was half that for surgical masks (200), meaning that our results tend to be less precise. Additionally, we found evidence that surgical masks were no less likely to be adopted than cloth masks. Surgical masks have higher filtration efficiency, are cheaper, are consistently worn, and are better supported by our evidence as tools to reduce COVID-19.

Our results should *not* be taken to imply that mask-wearing can prevent only 10% of COVID-19 cases, let alone 10% of COVID-19 mortality. Our intervention induced 29 more people out of every 100 to wear masks, with 42% of people wearing masks in total. The total impact with near-universal masking–perhaps achievable with alternative strategies or stricter enforcement–may be several times larger than our 10% estimate. Additionally, the intervention reduced symptomatic seroprevalence more when surgical masks were used, and even more for the highest-risk individuals in our sample (23% for ages 50-60 and 35% for ages 60+). These numbers likely give a better sense of the impact of our intervention on severe morbidity and mortality, since most of the disease burden of the COVID-19 pandemic is borne by the elderly. Where achievable, universal mask adoption is likely to have still larger impacts.

There are several possible theories for why we might observe a larger reduction in COVID-19 cases for older adults. We did not directly measure age during surveillance, but mask-wearing could have increased more for older adults. A second theory is that older adults are more susceptible to infections at viral loads preventable by masks. A third theory is that older adults have fewer social connections, so that reducing transmission through any one connection is more likely to prevent infection by severing all transmissible routes. A fourth theory is that people exercised more care and were more likely to wear masks when proximate to the elderly.

We identified a combination of core intervention elements that were effective in increasing mask-wearing in rural Bangladesh: mask distribution and role-modeling, combined with mask promotion, leads to large and sustained increases in mask use. Results from our pilots suggest that combining mask distribution, role-modeling, and active mask promotion – rather than mask distribution and role-modeling alone – seems critical to achieving the full effect. Our trial results also highlight many factors that appear inessential: we find no evidence that public commitments, village-level incentives, text

messages, altruistic messaging, or verbal commitments change mask-wearing behavior. The null results on our cross-randomizations do not necessarily imply that these approaches are not worth trying in other contexts, but they teach us that large, persistent increases in mask-wearing are possible without these elements.

Prediction studies we conducted with policymakers and public health experts at the World Health Organization and the World Bank prior to presentations of the study results suggests that our results are informative for policy design. The majority of respondents in the prediction studies anticipated that text messages, verbal commitments, and incentives would increase mask-wearing, when in reality, we estimated fairly precise null effects, and poll respondents believed that in-person mask promotion would have no additional effect, whereas the evidence from our pilots suggests it is essential (for additional detail see appendix R).

Our intervention design is immediately relevant for Bangladesh's plans for larger-scale distribution of masks across all rural areas. The Bangladesh Directorate-General of health has assigned the study team and the NGO *BRAC* the responsibility to scale up the strategies that were proven most effective in this trial to reach 81 million people (*35*). At the time of writing, we are implementing this program in the 37 districts prioritized by the government based on SARS-CoV-2 test positivity rates. Our results are also relevant for mask dissemination and promotion campaigns planned in other countries and settings which face similar challenges in ensuring mask usage as a result of limited reach and enforcement capacity. The mask promotion model described in this paper was subsequently adopted by governments and other implementers in Pakistan (*36*), India (*37*), and Nepal (*38*). The intervention package would be feasible to implement in a similar fashion in other world regions as well. Beyond face masks, the conceptual underpinning of our strategies could be applied to encourage the adoption of other health behaviors and technologies, in particular those easily observable by others outside the household, such as purchase and consumption of food, alcohol, and tobacco products in stores, restaurants, or other public spaces (*39*), hand washing and infection control in healthcare facilities (*40*–*42*), hygiene interventions in childcare and school settings (*43*, *44*), improved sanitation (*45*, *46*), or vaccination drives (*47*).

While critics of mask mandates suggest that individuals who wear masks are more likely to engage in high-risk behaviors (*48*), we found no evidence of risk compensation as a result of increased mask-wearing. In fact, we found that our intervention slightly increased the likelihood of physical distancing, presumably because individuals participating in the intervention took the threat of COVID-19 more seriously. These findings are consistent with other behaviors including seat belt use (*49*) or immunization (*50*) where risk compensation–even if present–is not sufficient to outweigh direct effects.

The intervention may have influenced rates of COVID-19 by increasing mask use and/or physical distancing and/or other risk prevention behaviors. Three factors suggest that the direct impact of masks is the most likely explanation for our documented health impacts. First, in appendix O, we analyze cross-sectionally the relationship between our biological outcomes and both mask-wearing and physical distancing. We find that symptoms and symptomatic seropositivity are negatively correlated with mask-wearing but not with physical distancing after controlling for mask-wearing. This analysis uses variation in observational rather than solely experimental data, and should therefore be interpreted with caution, as discussed in the appendix. Second, we see no change in physical distancing in the highest risk environment in our study, typically crowded indoor mosques. However, women do not typically go to mosques in rural Bangladesh and their symptomatic seropositivity decreased by just as much as men, so outdoor transmission or transmission in settings we do not observe directly may be important. Third, our study complements a large body of laboratory and quasi-experimental evidence that masks have a direct effect on SARS-CoV-2 transmission (*1*).

We estimate that a scaled version of our intervention being implemented in Bangladesh will cost between $10K and $52K per life saved, depending on what fraction of excess deaths are attributable to COVID-19. This is considerably lower than the value of a statistical life in Bangladesh ($205,000, (*51*)) and under severe outbreaks, is comparable to the most cost-efficient humanitarian programs at scale (e.g., distributing insecticide nets to prevent malaria costs $9,200 per life saved (*52*)). This estimate includes only mortality impacts but not morbidity, and greater cost-efficiency is possible if our intervention can be streamlined to further isolate the essential components. The vast majority of our costs were the personnel costs for mask-promoters: if we consider only the costs of mask production, these numbers would be 20x lower. Thus, the overall cost to save a life in countries where mask-mandates can be enforced at minimal cost with existing infrastructure may be substantially lower than our estimates above.

Study limitations

Our study has several limitations. The distinct appearance of project-associated masks and elevated mask-wearing in intervention villages made it impossible to blind surveillance staff to study arm assignment. However, staff were not informed about the exact purpose of the study. Even though surveillance staff were plain-clothed and were instructed to remain discreet, community members could have recognized that they were being observed and changed their behavior.

Additionally, survey respondents could have changed their likelihood of reporting symptoms in places where mask-wearing was more widespread. If respondents were more cognizant of symptoms in mask-wearing areas, this may bias us toward underestimating the impact of masks; if respondents in mask-wearing areas were less concerned with mild symptoms and thus were less likely to recall them, this might bias us toward overestimating the impact of masks. While we confirm that blood consent rates are not significantly different in the treatment and control group and are comparable across all demographic groups, we cannot rule out that the composition of consenters differed between the treatment and control groups. The slightly higher point estimate for consent in the treatment group biases us away from finding an effect, since it raises symptomatic seroprevalence in the treatment group. Although control villages were at least 2 km from intervention villages, adults from control villages may have come to intervention villages to receive masks, reducing the apparent impact of the intervention. While we did not directly assess harms in this study, there could be costs resulting from discomfort with increased mask-wearing, adverse health effects such as dermatitis or headaches, or impaired communication.

Because the study was powered to detect differences in symptomatic seroprevalence, we cannot distinguish whether masks work by making symptoms less severe (through a reduced viral load at transmission) or by reducing new infections. We selected the WHO case definition of COVID-19 for its sensitivity, though its limited specificity may imply that the impact of masks on symptoms comes partly from non-SARS-CoV-2 respiratory infections. If masks reduce COVID-19 by reducing symptoms (for a given number of infections), they could help ease the morbidity and mortality resulting from a given number of SARS-CoV-2 infections. If masks reduce infections, they may reduce the total number of infections over the long-term by buying more time to increase the fraction of the population vaccinated. At the time of the study, the predominant circulating SARS-CoV-2 strain was B.1.1.7 (alpha) (*53*). The impacts of the delta variant on the number of infections prevented by a given mask-wearer are uncertain; the population-wide consequences of infections prevented by a given mask-wearer may be larger given a higher reproduction number.

In summary, we found that mask distribution, role modeling, and promotion in a LMIC setting increased mask-wearing and physical distancing, leading to lower illness, particularly in older adults. We find especially robust evidence that surgical masks prevent COVID-19. Whether people with respiratory symptoms should generally wear masks to prevent respiratory virus transmission—including for viruses other than SARS-CoV-2—is an important area for future research. Our findings suggest that such behavior may benefit public health.

Methods and materials

Sampling frame and timeline

We discuss our sample size calculations in appendix B and discuss the selection and pairwise randomization in appendix C. In brief, we stratified villages based on geographic location and available case data, and then selected one treatment and control village from each pair.

Village-level cluster randomization was important for three reasons. First, unlike technologies with primarily private benefits, mask adoption is likely to yield especially large benefits at the community-level. Second, mask adoption by some may influence mask adoption by others because mask-wearing is immediately visible to other members of the community (*45*). Third, this design allows us to assess the full impact of masks on symptomatic infections, including via source control. Individual-level randomization would identify only whether masks protect wearers.

Our intervention was designed to last 8 weeks in each village. The intervention started in different villages at different times, rolling out over a 6-week period in 7 waves. There were between 16 and 61 village-pairs grouped in each wave based on geographic proximity and paired control and treatment villages were always included in the same wave. The first wave was rolled out on 17-18 November 2020 and the last wave was rolled out on 5-6 January 2021.

IPA staff travelled to many villages that had low mask uptake in the first five weeks of the study and found that in these villages local leaders were not very engaged in supporting mask promotion. Hence, we retrained mask promotion staff part-way through the intervention to work more closely with local leaders and set specific milestones for that partnership.

The intervention protocol, pre-specified analysis plan, and CONSORT checklist are available at https://osf.io/vzdh6/.

Outcomes

Our primary outcome was symptomatic seroprevalence of SARS-CoV-2. Our secondary outcomes were prevalence of proper mask-wearing, physical distancing, and symptoms consistent with COVID-19. For COVID-19 symptoms, we used the symptoms that correspond to the WHO case definition of probable COVID-19 given epidemiological risk factors: (a) fever and cough; (b) three or more of the following symptoms (fever, cough, general weakness/fatigue, headache, myalgia, sore throat, coryza, dyspnea, anorexia/nausea/vomiting, diarrhea, altered mental status); or (c) loss of taste or smell. Seropositivity was defined by having detectable IgG antibodies against SARS-CoV-2.

Intervention materials and activities

Our entire intervention was designed to be easily adopted by other NGOs or government agencies and required minimal monitoring. We have made the materials public in multiple languages to ease widespread adoption and replication by other implementers (https://osf.io/23mws/).

We provide design specifications for our masks in appendix F. We used high-quality surgical masks that had had a filtration efficiency of 95% [standard deviation (SD) = 1%]; this is substantially higher than the filtration efficiency of the cloth masks we designed, which had a filtration efficiency of 37% (SD = 6%). These cloth masks had substantially higher filtration than common commercial 3-ply cotton masks, but lower than hybrid masks that use materials not commonly available for community members in low-resource settings (*54*). While cloth masks have less leakage because they fit the face more closely (*55*) and can be sewn without specialized equipment, they are an order of magnitude more expensive than surgical masks. The filtration efficiency of the high-quality surgical masks used in this study was 76% after washing them with bar soap and water 10 times (manuscript forthcoming). While surgical masks can break down into microplastics that can enter the environment if disposed of improperly, analysis of waste generated in Bangladesh's first lockdown finds that the mass of surgical mask waste was one-third that of polyethylene bags, which also break down into macro- and micro-plastics (*56*–*58*).

Surgical masks were outfitted with a sticker that had a logo of a mask with an outline of the Bangladeshi flag and a phrase in Bengali that noted the mask could be washed and reused (*59*). The relatively large scale of our bulk order allowed us to negotiate mask prices of $0.50 per cloth mask and $0.13 per surgical mask ($0.06 of which was the cost of a sticker reminding people they could wash and reuse the surgical mask).

Adult household members were asked to wear masks whenever they were outside their house and around other people. To emphasize the importance of mask-wearing, we prepared a brief video of notable public figures discussing why, how, and when to wear a mask. The video was shown to each household during the mask distribution visit and featured the Honorable Prime Minister of Bangladesh Sheikh Hasina, the head of the Imam Training Academy, and the national cricket star Shakib Al Hasan. During the distribution visit, households also received a brochure based on WHO materials depicting proper mask-wearing.

We implemented a basic set of interventions in all treatment villages, and cross-randomized additional intervention elements in randomly chosen subsets of treatment villages to investigate whether those have any additional impact on mask-wearing. The basic intervention package consists of five main elements:

1. One-time mask distribution and information provision (about masks) at households
2. Mask distribution in markets on 3-6 days per week during all eight weeks of the intervention.
3. Mask distribution at mosques on three Fridays during the first four weeks of the intervention.
4. Mask promotion in public spaces and markets where non-mask wearers were encouraged to wear masks (weekly or biweekly).
5. Role-modeling and advocacy by local leaders, including imams discussing the importance of mask-wearing at Friday prayers using a scripted speech provided by the research team.

Participants and mask surveillance staff were not told which villages were in which intervention arm, but the intervention materials were clearly visible. The pre-specified analyses and sample exclusions were made by analysts blinded to the treatment assignment.

Cross-randomization of behavior change communication and incentives

Village-level cross-randomizations

Within the intervention arm, we cross-randomized villages to four village-level and four household-level treatments to test the impact of a range of social and behavior change communication strategies on mask-wearing. All intervention villages were assigned to either the treatment or the control group of each of these four randomizations. These village-level randomizations were:

1. Randomization of treated villages to either cloth or surgical masks.
2. Randomization of treated villages to public commitment (providing households signage and asking them to place signage on doors that declares they are a mask-wearing household), or not. The signage was meant to encourage formation of social norms through public signaling.
3. Randomization of treated villages to no incentive, non-monetary incentive, or monetary incentive of 190 USD given to the village leader for a project benefitting the public. We announced that the monetary reward or the certificate would be awarded if village-level mask-wearing among adults exceeded 75% 8-weeks after the intervention started.
4. Randomization of treated villages to 0% or 100% of households receiving twice-weekly text message reminders about the importance of mask-wearing.

Household-level cross-randomizations

We had three household-level cross-randomizations. In any single village, only one of these household randomizations was operative. As our data collection protocols relied on passive observation at the village-level, we could not record the mask-wearing behavior of individual households. To infer the

effect of the household-level treatments we therefore varied the color of the masks distributed to the household based on its cross-randomization status and had surveillance staff record the mask color of observed individuals. In surgical mask villages, a household received blue or green and promoters distributed and equal number of blue and green masks in public settings. In cloth mask villages, households received violet or red masks and promoters distributed blue masks in public settings. To avoid conflating the effect of the household-specific treatment with the effect of the mask color, we randomized which color corresponded to which treatment status across villages (this way a specific color was not fully coincident with a specific treatment). The household-level randomizations, described in further detail in appendix D and visualized in fig. S1, were:

1. Households were randomized to receive messages emphasizing either altruism or self-protection.
2. Households were randomized to making a verbal commitment to be a mask-wearing household (all adults in the household promise to wear a mask when they are outside and around other people) or not. This experiment was conducted in a third set of villages where there was no public signage commitment.
3. Households were randomized to receive twice-weekly text reminders or not. As mentioned above, the text message saturation was randomly varied to 0%, 50%, or 100% of all households receiving texts, and in the 50% villages, the specific households that received the texts was also random.

Conceptual basis for tested social and behavior change communication

We selected intervention elements that had a reasonable chance of persuading rural Bangladeshis to wear masks by consulting literature in public health, development and behavioral economics, and marketing to identify some of the most promising strategies. An extensive literature identifies price and access as key deterrents to the adoption of welfare-improving products, and especially of technologies that produce positive health externalities, such as face-masks (*21, 60*). Household distribution of free face-masks therefore formed the core part of our strategy. Inspired by large literature in marketing and economics on the role of opinion leaders in new product diffusion, we additionally emphasized a partnership with community leaders in mask distribution (*25*, *61*).

The additional village- and household-level treatment we experimented with were also motivated by insights from marketing, public health, development, and behavioral economics. For example, masks are a visible good where social norms are expected to be important, so we consulted the literature documenting peer effects in product adoption (*62*–*65*). We experimented with incentives because it is unclear whether extrinsic rewards crowd out intrinsic motivation (*66*–*68*). We test whether soft commitment devices encourage targets to follow through with actual behavior change (*69*, *70*), whether public displays can promote social norms (*27*), whether an altruistic framing inspires people more or less than self-interest (*71*), whether social image concerns and signaling can lead to higher compliance (*22*, *72*), and whether regular reminders are a useful tool to ensure adoption (*23*).

Piloting interventions

IPA implemented two pilots: Pilot 1 from July 22-31 and Pilot 2 from August 13-26, 2020. The objective of the pilots was to mimic some of the major aspects of the main experiment to identify implementation challenges. Each pilot was conducted in 10 unions that were not part of the main study area. We used the difference between the pilots to better understand which elements of our full intervention were essential. We also conducted focus group discussions and in-depth interviews with village residents, community leaders, religious leaders, and political leaders to elicit opinions on how to maximize the effectiveness of the intervention.

Surveillance strategies

Mask-wearing and physical distancing were measured through direct observation. Surveillance was conducted using a standard protocol that instructed staff to spend one hour at each of the following high-traffic locations in the village: market, restaurant entrances, main road, tea stalls, and mosque, changing the location and timing to record the mask-wearing and physical distancing practices of as many individuals as possible. While SARS-CoV-2 transmission is more likely in indoor locations with limited ventilation than outside, rural Bangladeshi villages have few non-residential spaces where people gather, so observations were conducted outside except at the mosque, where surveillance was conducted inside.

Surveillance staff were distinct from intervention implementation staff and conducted surveillance in paired intervention and control villages. To minimize the likelihood that village residents would perceive that their mask-wearing behavior was being observed, surveillance staff were separate from mask promoters and wore no identifying apparel while passively observing mask-wearing and physical distancing practices in the communities. They recorded the mask-wearing behavior of all of the adults they were able to observe during surveillance periods; observations were not limited to adults from enrolled households. Surveillance staff noted whether adults were wearing any mask or face covering, whether the mask was one distributed by our project (and if so, the color), and how the mask was worn. We defined proper mask-wearing as wearing either a project mask or an alternative face-covering over the mouth and nose and improper mask-wearing as wearing a mask in any way that did not fully cover the mouth and nose. Surveillance staff

observed a single individual and recorded that person as practicing physical distancing if s/he was at least one arm's length away from all other people. Additional details are in appendix G

Symptomatic SARS-CoV-2 testing

Symptom reporting

The owner of the household's primary phone completed surveys by phone or in-person at weeks 5 and 9 after the start of the intervention. They were asked to report symptoms experienced by any household member that occurred in the previous week and over the previous month. COVID-like symptoms were defined by whether they were consistent with the WHO COVID-19 case definition for suspected or probable cases with an epidemiological link (*73*).

Blood sample collection

We collected endline capillary blood samples from participants who reported COVID-like symptoms during the study period and consented to blood collection. We additionally collected samples on a subset of randomly-selected participants at baseline, independent of symptoms, to assess overall seropositivity. For the purposes of blood collection, endline was defined as 10-12 weeks from the start of the intervention. Blood samples were obtained by puncture with a 20-gauge safety lancet to the third or fourth digit. 500 microliters of blood were collected into Microtainer capillary blood collection serum separator tubes (BD, Franklin Lakes, NJ). Blood samples were transported on ice and stored at -20°C until testing.

SARS-CoV-2 testing

Blood samples were tested for the presence of IgG antibodies against SARS-CoV-2 using the SCoV-2 Detect IgG ELISA kit (InBios, Seattle, Washington). This assay detects IgG antibodies against the spike protein subunit (S1) of SARS-CoV-2. The assays were performed according to the manufacturer's instructions. Additional details are presented in appendix H.

Symptomatic seropositivity

Our primary outcome is symptomatic seropositivity. As noted above, individuals are symptomatic if they 1) meet the WHO surveillance definition of probable COVID-19 and 2) are seropositive in our blood test at endline. If either of these conditions fail to hold, $Y_{ij} = 0$. To assess seropositivity, we tested all individuals who were symptomatic in either our 5-week or 9-week household survey.

Our goal is to estimate the impact of the intervention on symptomatic seropositivity, defined as: $\psi_0 = E_x[E(Y_{ij} \mid T_j = 1, x_j) - E(Y_{ij} \mid T_j = 0, x_j)]$ where T_j is an indicator for whether a village was treated and x_j are village-level covariates including baseline mask-use in each village (constructed as described below) and baseline influenza-like illness and COVID-19 based on reported symptoms, as well as indicators for each pair of villages from our pairwise stratification method.

In our pre-registered specification, we estimate this parameter by ordinary least squares, clustering at the village-level using the approach in (*74*–*76*). The dependent variable is Y_{ij}, the independent variable of interest is T_j, and controls are included for the x_j covariates, including baseline mask-use and baseline respiratory symptom rates in each village. We also report results from a generalized linear model with a Poisson family and log-link function to compute relative risk (*77*). More details of our statistical analyses are reported in appendix K.

REFERENCES AND NOTES

1. J. Howard, A. Huang, Z. Li, Z. Tufekci, V. Zdimal, H.-M. van der Westhuizen, A. von Delft, A. Price, L. Fridman, L.-H. Tang, V. Tang, G. L. Watson, C. E. Bax, R. Shaikh, F. Questier, D. Hernandez, L. F. Chu, C. M. Ramirez, A. W. Rimoin, An evidence review of face masks against COVID-19. *Proc. Natl. Acad. Sci. U.S.A.* **118**, e2014564118 (2021). doi:10.1073/pnas.2014564118 Medline
2. N. H. L. Leung, D. K. W. Chu, E. Y. C. Shiu, K.-H. Chan, J. J. McDevitt, B. J. P. Hau, H.-L. Yen, Y. Li, D. K. M. Ip, J. S. M. Peiris, W.-H. Seto, G. M. Leung, D. K. Milton, B. J. Cowling, Respiratory virus shedding in exhaled breath and efficacy of face masks. *Nat. Med.* **26**, 676–680 (2020). doi:10.1038/s41591-020-0843-2 Medline
3. C. R. MacIntyre, A. A. Chughtai, Facemasks for the prevention of infection in healthcare and community settings. *BMJ* **350**, h694 (2015). doi:10.1136/bmj.h694 Medline
4. H. Bundgaard, J. S. Bundgaard, D. E. T. Raaschou-Pedersen, C. von Buchwald, T. Todsen, J. B. Norsk, M. M. Pries-Heje, C. R. Vissing, P. B. Nielsen, U. C. Winsløw, K. Fogh, R. Hasselbalch, J. H. Kristensen, A. Ringgaard, M. Porsborg Andersen, N. B. Goecke, R. Trebbien, K. Skovgaard, T. Benfield, H. Ullum, C. Torp-Pedersen, K. Iversen, Effectiveness of adding a mask recommendation to other public health measures to prevent SARS-CoV-2 infection in Danish mask wearers: A randomized controlled trial. *Ann. Intern. Med.* **174**, 335–343 (2021). doi:10.7326/M20-6817 Medline
5. C. N. Ngonghala, E. Iboi, S. Eikenberry, M. Scotch, C. R. MacIntyre, M. H. Bonds, A. B. Gumel, Mathematical assessment of the impact of non-pharmaceutical interventions on curtailing the 2019 novel Coronavirus. *Math. Biosci.* **325**, 108364 (2020). doi:10.1016/j.mbs.2020.108364 Medline
6. C. T. Leffler, E. Ing, J. D. Lykins, M. C. Hogan, C. A. McKeown, A. Grzybowski, Association of country-wide coronavirus mortality with demographics, testing, lockdowns, and public wearing of masks. *Am. J. Trop. Med. Hyg.* **103**, 2400–2411 (2020). doi:10.4269/ajtmh.20-1015 Medline
7. W. Lyu, G. L. Wehby, Community use of face masks and COVID-19: Evidence from a natural experiment of state mandates in the US. *Health Aff.* **39**, 1419–1425 (2020). doi:10.1377/hlthaff.2020.00818 Medline
8. V. Chernozhukov, H. Kasahara, P. Schrimpf, Causal impact of masks, policies, behavior on early covid-19 pandemic in the U.S. *J. Econom.* **220**, 23–62 (2021). doi:10.1016/j.jeconom.2020.09.003 Medline
9. J. Abaluck, J. A. Chevalier, N. A. Christakis, H. P. Forman, E. H. Kaplan, A. Ko, S. H. Vermund, The case for universal cloth mask adoption and policies to increase supply of medical masks for health workers. SSRN 3567438 [Preprint] (2020); https://dx.doi.org/10.2139/ssrn.3567438.
10. Y. Cheng, N. Ma, C. Witt, S. Rapp, P. S. Wild, M. O. Andreae, U. Pöschl, H. Su, Face masks effectively limit the probability of SARS-CoV-2 transmission. *Science* **372**, 1439–1443 (2021). doi:10.1126/science.abg6296 Medline
11. A. Mullard, "How COVID vaccines are being divvied up around the world," *Nature News*, 30 November 2020.
12. T. A. Ghebreyesus, WHO Director-General's opening remarks at the media briefing

on COVID-19 - 5 June 2020 (World Health Organization, 2020).
13. L. M. Brosseau, M. Sietsema, "Commentary: Masks-for-all for COVID-19 not based on sound data" (Center for Infectious Disease Research and Policy, University of Minnesota, 2020); www.cidrap.umn.edu/news-perspective/2020/04/commentary-masks-all-covid-19-not-based-sound-data.
14. M. A. Johansson, T. M. Quandelacy, S. Kada, P. V. Prasad, M. Steele, J. T. Brooks, R. B. Slayton, M. Biggerstaff, J. C. Butler, SARS-CoV-2 transmission from people without COVID-19 symptoms. *JAMA Netw. Open* **4**, e2035057 (2021). doi:10.1001/jamanetworkopen.2020.35057 Medline
15. Centers for Disease Control and Prevention (CDC), "Science brief: Community use of cloth masks to control the spread of SARS-CoV-2" (CDC, 2021).
16. J. M. Brophy, "Covid-19: Controversial trial may actually show that masks protect the wearer," *BMJ Opinion*, 24 November 2020.
17. J. Pan, C. Harb, W. Leng, L. C. Marr, Inward and outward effectiveness of cloth masks, a surgical mask, and a face shield. *Aerosol Sci. Technol.* **55**, 718–733 (2021). doi:10.1080/02786826.2021.1890687
18. D. Kahneman, D. T. Miller, Norm theory: Comparing reality to its alternatives. *Psychol. Rev.* **93**, 136–153 (1986). doi:10.1037/0033-295X.93.2.136
19. J. Jordan, E. Yoeli, D. Rand, Don't get it or don't spread it: Comparing self-interested versus prosocial motivations for COVID-19 prevention behaviors. PsyArXiv [Preprint] (2020); https://doi.org/10.31234/osf.io/yuq7x.
20. R. B. Cialdini, N. J. Goldstein, Social influence: Compliance and conformity. *Annu. Rev. Psychol.* **55**, 591–621 (2004). doi:10.1146/annurev.psych.55.090902.142015 Medline
21. M. Bates, R. Glennerster, K. Gumede, E. Duflo, The price is wrong. *Field Actions Sci. Rep.* **4**, 30 (2012).
22. A. Karing, "Social signaling and childhood immunization: A field experiment in Sierra Leone," Working paper, University of California, Berkeley (2018).
23. D. Karlan, M. McConnell, S. Mullainathan, J. Zinman, Getting to the top of mind: How reminders increase saving. *Manage. Sci.* **62**, 3393–3411 (2016). doi:10.1287/mnsc.2015.2296
24. N. J. Goldstein, R. B. Cialdini, V. Griskevicius, A room with a viewpoint: Using social norms to motivate environmental conservation in hotels. *J. Consum. Res.* **35**, 472–482 (2008). doi:10.1086/586910
25. G. Miller, A. M. Mobarak, Learning about new technologies through social networks: Experimental evidence on nontraditional stoves in Bangladesh. *Mark. Sci.* **34**, 480–499 (2014). doi:10.1287/mksc.2014.0845
26. P. Manchanda, Y. Xie, N. Youn, The role of targeted communication and contagion in product adoption. *Mark. Sci.* **27**, 961–976 (2008). doi:10.1287/mksc.1070.0354
27. C. Bicchieri, *Norms in the Wild: How to Diagnose, Measure, and Change Social Norms* (Oxford Univ. Press, 2016).
28. T. R. Bhuiyan, J. D. Hulse, S. T. Hegde, M. Akhtar, M. T. Islam, Z. H. Khan, I. I. Khan, S. Ahmed, M. M. Rashid, R. Rashid, E. S. Gurley, T. Shirin, A. I. Khan, A. S. Azman, F. Qadri, SARS-CoV-2 seroprevalence in Chattogram, Bangladesh before the Delta surge, March-June 2021. medRxiv 2021.07.16.21260611 [Preprint] (2021); https://doi.org/10.1101/2021.07.16.21260611.
29. icddr,b, "Higher covid-19 seropositivity observed among residents in Dhaka and Chattogram," 22 June 2021; www.icddrb.org/news-and-events/news?id=878.
30. Management Information System (MIS), Directorate General of Health Services (DGHS), COVID-19 dynamic dashboard for Bangladesh (2021); http://dashboard.dghs.gov.bd/webportal/pages/covid19.php [accessed 16 August 2021].
31. M. V. Murhekar, T. Bhatnagar, J. W. V. Thangaraj, V. Saravanakumar, M. S. Kumar, S. Selvaraju, K. Rade, C. P. G. Kumar, R. Sabarinathan, A. Turuk, S. Asthana, R. Balachandar, S. D. Bangar, A. K. Bansal, V. Chopra, D. Das, A. K. Deb, K. R. Devi, V. Dhikav, G. R. Dwivedi, S. M. S. Khan, M. S. Kumar, A. Laxmaiah, M. Madhukar, A. Mahapatra, C. Rangaraju, J. Turuk, R. Yadav, R. Andhalkar, K. Arunraj, D. K. Bharadwaj, P. Bharti, D. Bhattacharya, J. Bhat, A. S. Chahal, D. Chakraborty, A. Chaudhury, H. Deval, S. Dhatrak, R. Dayal, D. Elantamilan, P. Giridharan, I. Haq, R. K. Hudda, B. Jagjeevan, A. Kalliath, S. Kanungo, N. N. Krishnan, J. S. Kshatri, A. Kumar, N. Kumar, V. G. V. Kumar, G. G. J. N. Lakshmi, G. Mehta, N. K. Mishra, A. Mitra, K. Nagbhushanam, A. Nimmathota, A. R. Nirmala, A. K. Pandey, G. V. Prasad, M. A. Qurieshi, S. D. Reddy, A. Robinson, S. Sahay, R. Saxena, K. Sekar, V. K. Shukla, H. B. Singh, P. K. Singh, P. Singh, R. Singh, N. Srinivasan, D. S. Varma, A. Viramgami, V. C. Wilson, S. Yadav, S. Yadav, K. Zaman, A. Chakrabarti, A. Das, R. S. Dhaliwal, S. Dutta, R. Kant, A. M. Khan, K. Narain, S. Narasimhaiah, C. Padmapriyadarshini, K. Pandey, S. Pati, S. Patil, H. Rajkumar, T. Ramarao, Y. K. Sharma, S. Singh, S. Panda, D. C. S. Reddy, B. Bhargava, ICMR Serosurveillance Group, SARS-CoV-2 seroprevalence among the general population and healthcare workers in India, December 2020-January 2021. *Int. J. Infect. Dis.* **108**, 145–155 (2021). doi:10.1016/j.ijid.2021.05.040 Medline
32. A. Anand, J. Sandefur, A. Subramanian, "Three new estimates of India's all-cause excess mortality during the COVID-19 pandemic, Working paper no. 589, Center for Global Development, July 2021.
33. J. Abaluck, A. M. Mobarak, "Getting all Bangladeshis to wear masks," *WhiteBoard Magazine*, 1 December 2020.
34. A. Jakubowski, D. Egger, C. Nekesa, L. Lowe, M. Walker, E. Miguel, Self-reported mask wearing greatly exceeds directly observed use: Urgent need for policy intervention in Kenya. medRxiv 2021.01.27.21250487 [Preprint] (2021); https://doi.org/10.1101/2021.01.27.21250487.
35. K. K. Tithila, "Brac's efforts to mask up Bangladesh could be game-changer," *Dhaka Tribune* (Bangladesh), 15 July 2021.
36. S. Riaz, "Punjab authorities kick off 'NORM' campaign to increase mask-wearing," *Arab New* (Pakistan), 30 June 2021.
37. S. Bhattacharjee, "Covid-19 Crisis: India draws lessons from Bangladesh's mask study," *Business Standard* (India), 15 May 2021.
38. Republica, "Nepal Mask Campaign launches with the slogan 'Let's wear masks, let's save each other's lives'," *myRepública*, 16 August 2021).
39. G. J. Hollands, P. Carter, S. Answer, S. E. King, S. A. Jebb, D. Ogilvie, I. Shemilt, J. P. T. Higgins, T. M. Marteau, Altering the availability or proximity of food, alcohol, and tobacco products to change their selection and consumption. *Cochrane Database Syst. Rev.* **2019**, CD012576 (2019). doi:10.1002/14651858.CD012573.pub3 Medline
40. S. Naikoba, A. Hayward, The effectiveness of interventions aimed at increasing handwashing in healthcare workers – a systematic review. *J. Hosp. Infect.* **47**, 173–180 (2001). doi:10.1053/jhin.2000.0882 Medline
41. C. Houghton, P. Meskell, H. Delaney, M. Smalle, C. Glenton, A. Booth, X. H. S. Chan, D. Devane, L. M. Biesty, Barriers and facilitators to healthcare workers' adherence with infection prevention and control (IPC) guidelines for respiratory infectious diseases: A rapid qualitative evidence synthesis. *Cochrane Database Syst. Rev.* **4**, CD013582 (2020). doi:10.1002/14651858.CD013582 Medline
42. H. J. Seo, K.-Y. Sohng, S. O. Chang, S. K. Chaung, J. S. Won, M.-J. Choi, Interventions to improve hand hygiene compliance in emergency departments: A systematic review. *J. Hosp. Infect.* **102**, 394–406 (2019). doi:10.1016/j.jhin.2019.03.013 Medline
43. D. Biswas, M. Ahmed, K. Roguski, P. K. Ghosh, S. Parveen, F. A. Nizame, M. Z. Rahman, F. Chowdhury, M. Rahman, S. P. Luby, K. Sturm-Ramirez, A. D. Iuliano, Effectiveness of a behavior change intervention with hand sanitizer use and respiratory hygiene in reducing laboratory-confirmed influenza among schoolchildren in Bangladesh: A cluster randomized controlled trial. *Am. J. Trop. Med. Hyg.* **101**, 1446–1455 (2019). doi:10.4269/ajtmh.19-0376 Medline
44. S. L. McGuinness, S. F. Barker, J. O'Toole, A. C. Cheng, A. B. Forbes, M. Sinclair, K. Leder, Effect of hygiene interventions on acute respiratory infections in childcare, school and domestic settings in low- and middle-income countries: A systematic review. *Trop. Med. Int. Health* **23**, 816–833 (2018). doi:10.1111/tmi.13080 Medline
45. R. Guiteras, J. Levinsohn, A. M. Mobarak, Sanitation subsidies. Encouraging sanitation investment in the developing world: A cluster-randomized trial. *Science* **348**, 903–906 (2015). doi:10.1126/science.aaa0491 Medline
46. S. R. Patil, B. F. Arnold, A. L. Salvatore, B. Briceno, S. Ganguly, J. M. Colford Jr., P. J. Gertler, The effect of India's total sanitation campaign on defecation behaviors and child health in rural Madhya Pradesh: A cluster randomized controlled trial. *PLOS Med.* **11**, e1001709 (2014). doi:10.1371/journal.pmed.1001709 Medline
47. J. S. Solís Arce, S. S. Warren, N. F. Meriggi, A. Scacco, N. McMurry, M. Voors, G. Syunyaev, A. A. Malik, S. Aboutajdine, O. Adeojo, D. Anigo, A. Armand, S. Asad, M. Atyera, B. Augsburg, M. Awasthi, G. E. Ayesiga, A. Bancalari, M. Björkman Nyqvist, E. Borisova, C. M. Bosancianu, M. R. Cabra García, A. Cheema, E. Collins, F. Cuccaro, A. Z. Farooqi, T. Fatima, M. Fracchia, M. L. Galindo Soria, A. Guariso, A. Hasanain, S. Jaramillo, S. Kallon, A. Kamwesigye, A. Kharel, S. Kreps, M. Levine, R. Littman, M. Malik, G. Manirabaruta, J. L. H. Mfura, F. Momoh, A. Mucauque, I.

Mussa, J. A. Nsabimana, I. Obara, M. J. Otálora, B. W. Ouédraogo, T. B. Pare, M. R. Platas, L. Polanco, J. A. Qureshi, M. Raheem, V. Ramakrishna, I. Rendrá, T. Shah, S. E. Shaked, J. N. Shapiro, J. Svensson, A. Tariq, A. M. Tchibozo, H. A. Tiwana, B. Trivedi, C. Vernot, P. C. Vicente, L. B. Weissinger, B. Zafar, B. Zhang, D. Karlan, M. Callen, M. Teachout, M. Humphreys, A. M. Mobarak, S. B. Omer, COVID-19 vaccine acceptance and hesitancy in low- and middle-income countries. *Nat. Med.* **27**, 1385–1394 (2021). doi:10.1038/s41591-021-01454-y Medline
48. Y. Yan, J. Bayham, A. Richter, E. P. Fenichel, Risk compensation and face mask mandates during the COVID-19 pandemic. *Sci. Rep.* **11**, 3174 (2021). doi:10.1038/s41598-021-82574-w Medline
49. A. Cohen, L. Einav, Estimating risk preferences from deductible choice. *Am. Econ. Rev.* **97**, 745–788 (2007). doi:10.1257/aer.97.3.745
50. M. L. Kasting, G. K. Shapiro, Z. Rosberger, J. A. Kahn, G. D. Zimet, Tempest in a teapot: A systematic review of HPV vaccination and risk compensation research. *Hum. Vaccin. Immunother.* **12**, 1435–1450 (2016). doi:10.1080/21645515.2016.1141158 Medline
51. W. K. Viscusi, C. J. Masterman, Income elasticities and global values of a statistical life. *J. Benefit Cost Anal.* **8**, 226–250 (2017). doi:10.1017/bca.2017.12
52. GiveWell, 2021 GiveWell cost-effectiveness analysis – Version 1 (2021); www.givewell.org/how-we-work/our-criteria/cost-effectiveness/cost-effectiveness-models/changelog-2021#Version_1_Published_May_5_2021 [accessed 4 January 2021].
53. J. Hadfield, C. Megill, S. M. Bell, J. Huddleston, B. Potter, C. Callender, P. Sagulenko, T. Bedford, R. A. Neher, Nextstrain: Real-time tracking of pathogen evolution. *Bioinformatics* **34**, 4121–4123 (2018). doi:10.1093/bioinformatics/bty407 Medline
54. L. H. Kwong, R. Wilson, S. Kumar, Y. S. Crider, Y. Reyes Sanchez, D. Rempel, A. Pillarisetti, Review of the breathability and filtration efficiency of common household materials for face masks. *ACS Nano* **15**, 5904–5924 (2021). doi:10.1021/acsnano.0c10146 Medline
55. S. Duncan, P. Bodurtha, S. Naqvi, The protective performance of reusable cloth face masks, disposable procedure masks, KN95 masks and N95 respirators: Filtration and total inward leakage. *PLOS ONE* **16**, e0258191 (2021). doi:10.1371/journal.pone.0258191 Medline
56. O. O. Fadare, E. D. Okoffo, Covid-19 face masks: A potential source of microplastic fibers in the environment. *Sci. Total Environ.* **737**, 140279 (2020). doi:10.1016/j.scitotenv.2020.140279 Medline
57. Environment and Social Development Organization (ESDO), "COVID-19 pandemic pushes single use plastic waste outbreak: No management, no protection: High health and environmental risk unveil" (ESDO, 2020).
58. I. M. Steensgaard, K. Syberg, S. Rist, N. B. Hartmann, A. Boldrin, S. F. Hansen, From macro- to microplastics – Analysis of EU regulation along the life cycle of plastic bags. *Environ. Pollut.* **224**, 289–299 (2017). doi:10.1016/j.envpol.2017.02.007 Medline
59. E.-S. Jang, C.-W. Kang, Do face masks become worthless after only one use in the COVID-19 pandemic? *Infect. Chemother.* **52**, 583–591 (2020). doi:10.3947/ic.2020.52.4.583 Medline
60. M. Kremer, E. Miguel, The illusion of sustainability. *Q. J. Econ.* **122**, 1007–1065 (2007). doi:10.1162/qjec.122.3.1007
61. P. S. van Eck, W. Jager, P. S. Leeflang, Opinion leaders' role in innovation diffusion: A simulation study. *J. Prod. Innov. Manage.* **28**, 187–203 (2011). doi:10.1111/j.1540-5885.2011.00791.x
62. E. Oster, R. Thornton, Determinants of technology adoption: Peer effects in menstrual cup take-up. *J. Eur. Econ. Assoc.* **10**, 1263–1293 (2012). doi:10.1111/j.1542-4774.2012.01090.x
63. H. Allcott, Social norms and energy conservation. *J. Public Econ.* **95**, 1082–1095 (2011). doi:10.1016/j.jpubeco.2011.03.003
64. R. Guiteras, J. Levinsohn, A. M. Mobarak, "Demand estimation with strategic complementarities: Sanitation in Bangladesh," Discussion paper no. DP13498, Centre for Economic Policy Research, January 2019).
65. L. Beaman, A. BenYishay, J. Magruder, A. M. Mobarak, Can network theory-based targeting increase technology adoption? *Am. Econ. Rev.* **111**, 1918–1943 (2021). doi:10.1257/aer.20200295
66. N. Ashraf, O. Bandiera, K. Jack, No margin, no mission? A field experiment on incentives for public service delivery. *J. Public Econ.* **120**, 1–17 (2014). doi:10.1016/j.jpubeco.2014.06.014
67. R. Chetty, E. Saez, L. Sandor, What policies increase prosocial behavior? An experiment with referees at the *Journal of Public Economics*. *J. Econ. Perspect.* **28**, 169–188 (2014). doi:10.1257/jep.28.3.169
68. D. Ariely, A. Bracha, S. Meier, Doing good or doing well? Image motivation and monetary incentives in behaving prosocially. *Am. Econ. Rev.* **99**, 544–555 (2009). doi:10.1257/aer.99.1.544
69. G. Bryan, D. Karlan, S. Nelson, Commitment devices. *Annu. Rev. Econ.* **2**, 671–698 (2010). doi:10.1146/annurev.economics.102308.124324
70. J. Luoto, D. Levine, J. Albert, S. Luby, Nudging to use: Achieving safe water behaviors in Kenya and Bangladesh. *J. Dev. Econ.* **110**, 13–21 (2014). doi:10.1016/j.jdeveco.2014.02.010
71. N. Ashraf, O. Bandiera, E. Davenport, S. S. Lee, Losing prosociality in the quest for talent? Sorting, selection, and productivity in the delivery of public services. *Am. Econ. Rev.* **110**, 1355–1394 (2020). doi:10.1257/aer.20180326
72. L. Bursztyn, R. Jensen, Social image and economic behavior in the field: Identifying, understanding, and shaping social pressure. *Annu. Rev. Econ.* **9**, 131–153 (2017). doi:10.1146/annurev-economics-063016-103625
73. World Health Organization (WHO), WHO COVID-19 case definition (2020); www.who.int/publications/i/item/WHO-2019-nCoV-Surveillance_Case_Definition-2020.2 [accessed on 15 October 2020].
74. P. Guimarães, P. Portugal, A simple feasible procedure to fit models with high-dimensional fixed effects. *Stata J.* **10**, 628–649 (2010). doi:10.1177/1536867X1101000406
75. S. Gaure, "OLS with multiple high dimensional category dummies," Memorandum 14/2010, Oslo University (2011).
76. P. Guimarães, P. Portugal, A simple feasible procedure to fit models with high-dimensional fixed effects. *Stata J.* **10**, 628–649 (2011). doi:10.1177/1536867X1101000406
77. G. Zou, A modified poisson regression approach to prospective studies with binary data. *Am. J. Epidemiol.* **159**, 702–706 (2004). doi:10.1093/aje/kwh090 Medline
78. J. Abaluck, L. H. Kwong, A. Styczynski, A. Haque, M. A. Kabir, E. Bates-Jefferys, E. Crawford, J. Benjamin-Chung, S. Raihan, S. Rahman, S. Benhachmi, N. Z. Bintee, P. J. Winch, M. Hossain, H. M. Reza, A. A. Jaber, S. Gulshan Momen, A. Rahman, F. L. Banti, T. S. Huq, S. P. Luby, A. M. Mobarak, Impact of community masking on COVID-19: A cluster-randomized trial in Bangladesh. Zenodo (2021); https://doi.org/10.5281/zenodo.5703876.
79. C. Rutterford, A. Copas, S. Eldridge, Methods for sample size determination in cluster randomized trials. *Int. J. Epidemiol.* **44**, 1051–1067 (2015). doi:10.1093/ije/dyv113 Medline
80. Institute for Health Metrics and Evaluation (IHME), COVID-19 projections – Bangladesh (2021); https://covid19.healthdata.org/bangladesh?view=cumulative-deaths&tab=trend [accessed 17 August 2021].
81. WHO Bangladesh, COVID-19 Morbidity and Mortality Weekly Update, vol. 76, 9 August 2021; https://cdn.who.int/media/docs/default-source/searo/bangladesh/covid-19-who-bangladesh-situation-reports/who_covid-19-update_76_20210809.pdf?sfvrsn=9208a5a_9.

ACKNOWLEDGMENTS

Thanks to Dr. Sabrina Flora, additional director general of the Directorate General of Health Services in Bangladesh, James Snowden and Karen Levy for ongoing support and encouragement. Thanks to WHO Chief Scientist Soumya Swaminathan for her encouragement to conduct this trial. Thanks to Anir Chowdhury, policy advisor to the Bangladesh government, and Dr. Shams El Arifeen of icddr,b for establishing connections with the Bangladesh Directorate General of Health Services in the Ministry of Health and Family Welfare. Thanks to Dr. Michael Friedman and the CDC Bangladesh country office for assistance with antibody tests. Thanks to Asif Saleh, Executive Director of BRAC, Dr. Morseda Chowdhury, Tanjila Mazumder Drishti, Imran Ahmed Chowdhury, and hundreds of BRAC staff for help with implementation, especially in the scale-up phase of this project in Bangladesh. Similarly, Reema Nanavaty, Sahil Hebbar, and other staff of SEWA India, and Captain Usman and his team at the Lahore Commissioners office, the Nepal Rapid Action Taskforce (C19 RAT), Senator Carmen Sanguinetti, Mario Sanchez, and Florencia Lopez Boo (IADB) played

crucial roles in replication and scale-ups in India, Pakistan, Nepal and Uruguay. Thanks also to Judy Chevalier, Bhavani Prathap Kasina and Stacey Daves-Ohlin for their advice and assistance, Tom Schmidt for help in shipping, Peter Hull for econometrics consulting, Arnab Bhattacharya of the Tata Institute of Fundamental Research and Shailabh Kumar of Stanford University for conducting filtration efficiency testing of the study masks and evaluation of the impact of washing on surgical mask performance, Yasser Choudhury from Katex for assistance with the surgical masks, Anisur Rahman from Standard Group for assistance with the cloth masks, GreenVoice, Adam Gsellman for help with visualizations, and many employees at IPA Bangladesh for assistance throughout this project. Most importantly, our continued engagement in and scaling up of mask promotion would not be possible without the tireless efforts of the Mask-NORM team, including Neela Saldanha, Heidi McAnnally-Linz, Maha Rehman, Gautam Patel, Jose Pinilla, Janani Rajashekar, Preeti Adhikary, Sharon Barnhardt, Mehrab Ali, Urvashi Wattal, Islamul Haque, Ana Tamayo, Laura Burke, Jeffrey Mosenkis, and many other staff of Yale University and of Innovations for Poverty Action. **Funding:** This research was financially supported by a grant from GiveWell.org to Innovations for Poverty Action (grant GR-000000272). J. Benjamin-Chung was supported by the National Institute of Allergy and Infectious Diseases, National Institutes of Health (grant K01AI141616). **Research ethics approvals:** Our study protocols were reviewed and approved by the Yale University Institutional Review Board (Protocol ID: 2000028482), and by the Bangladesh Medical Research Council National Research Ethics Committee (IRB registration number: 330 26 08 2020). We also received separate administrative approval from the Bangladesh Ministry of Health and Family Welfare. The Bangladesh Directorate General of Health Services under the Ministry of Health, Aspire to Innovate (a2i), an information and data-focused organization within the Bangladesh government, North-South University in Dhaka, and the International Centre for Diarrhoeal Disease Research, Bangladesh (icddr,b) partnered in the study design and discussions and reviewed protocols. We provide ethical justification for our decisions in our online ethics appendix (https://osf.io/m2bwq/). **Author contributions:** Conceptualization: JA, LHK, AS, SPL, AMM; Methodology: JA LHK, AS, JBC, PJW, SPL, AMM; Software: EC; Validation: EC; Formal Analysis: JA, EC; Investigation: LHK, AS, MH, HMR, AAJ, SGM, AR, FLB, TSH; Resources: EBJ, SB; Data Curation: SRahman, EC, NZ; Writing: JA, LHK, AS, EC, SPL, AMM; Visualization: EC, NZ; Supervision: JA, SPL, AMM; Project Administration: JA, LHK, AH, MAK, SRaihan, SRahman; Funding Acquisition: JA, AMM, LHK, AS, SPL. **Competing interests:** The funder had no role in the study design, interpretation of results, or decision to publish. The authors declare no competing interests. **Data and materials availability:** This clinical trial has been registered at clinicaltrials.gov (identifier NCT04630054). All data and code are provided in our online repository (https://gitlab.com/emily-crawford/bd-mask-rct) (*78*). This work is licensed under a Creative Commons Attribution 4.0 International (CC BY 4.0) license, which permits unrestricted use, distribution, and reproduction in any medium, provided the original work is properly cited. To view a copy of this license, visit https://creativecommons.org/licenses/by/4.0/. This license does not apply to figures/photos/artwork or other content included in the article that is credited to a third party; obtain authorization from the rights holder before using such material.

SUPPLEMENTARY MATERIALS

science.org/doi/10.1126/science.abi9069
Figs. S1 to S6
Tables S1 to S37
Appendices
References (*79*–*81*)
MDAR Reproducibility Checklist

8 April 2021; accepted 23 November 2021
Published online 2 December 2021
10.1126/science.abi9069

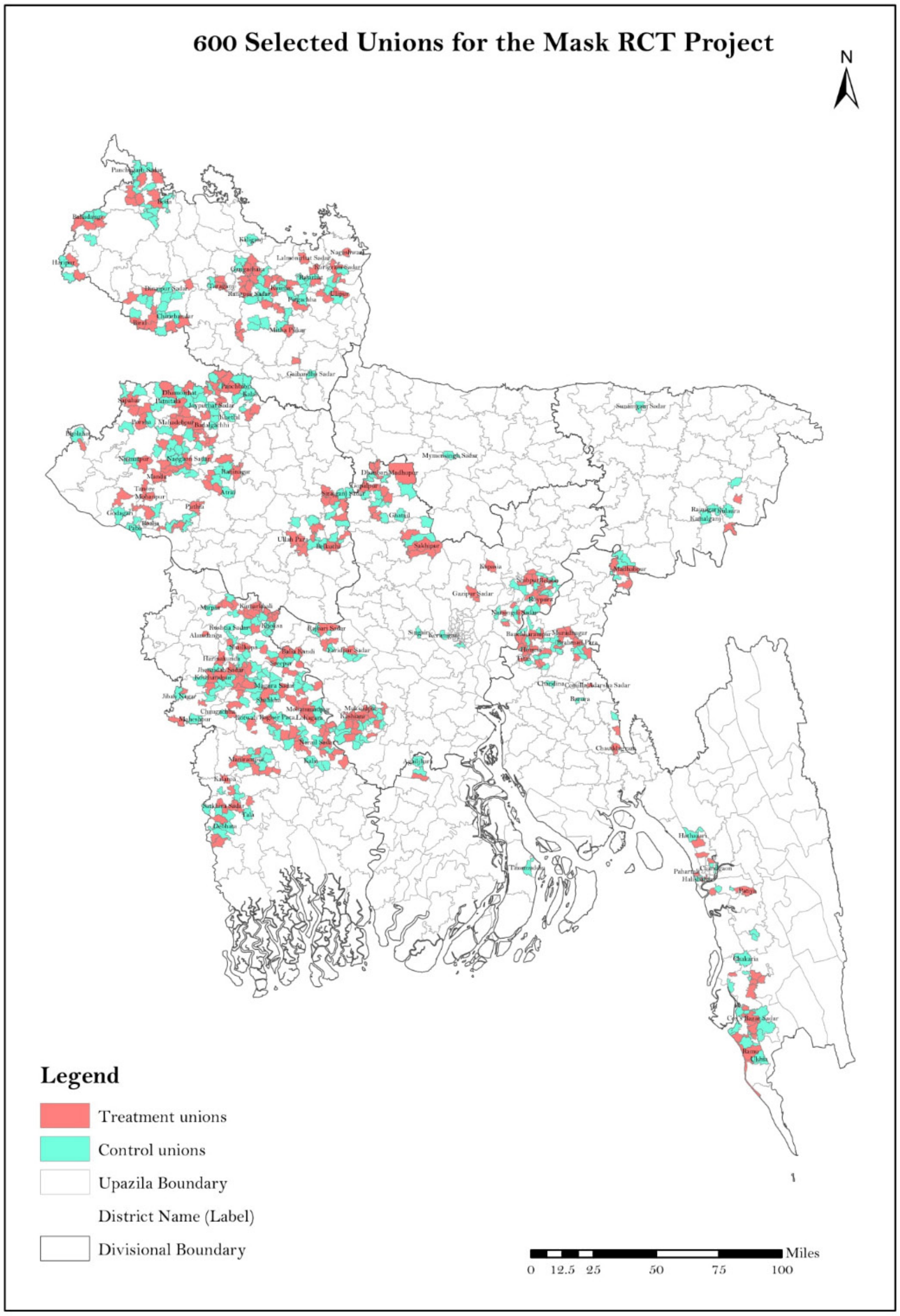

Fig. 1. Map of 600 treatment and control unions. The figure shows the location of the 600 treatment and control unions in the study.

Table 1. Mask-wearing and physical distancing, controlling for baseline variables. All regressions include an indicator for each control-intervention pair and baseline symptom rates. The analyses in the top panel control for baseline rates of proper mask wearing, and the analyses in the bottom panel control for baseline rates of physical distancing. Baseline symptom rate is defined as the rate of surveyed individuals in a village who report symptoms coinciding with the WHO definition of a probable COVID-19 case. We assume that (1) all reported symptoms were acute onset, (2) all people live or work in an area with high risk of transmission of virus and (3) all people have been a contact of a probable or confirmed case of COVID-19 or are linked to a COVID-19 cluster. "No Active Promotion" refers to any time that surveillance was conducted while promotion was not actively occurring (regardless of the week of the intervention). This excludes surveillance during the Friday Jumma Prayers in the mosque, when promoters were present and actively encouraged mask wearing. "Other Locations" include tea stalls, at the entrance of the restaurant as patrons enter, and the main road to enter the village. "Surgical Villages" refer to all treatment villages which received surgical masks as part of the intervention, and their control pairs. "Cloth Villages" refer to all treatment villages which received cloth masks as part of the intervention, and their control pairs. The surgical and cloth sub-samples include surveillance from all available locations, equivalent to the to the column labeled "Full", but run separately for each subgroup. Of the 572 villages included in the analyses sample, we exclude an additional village and its pair in the mosque and market sub-samples, and two villages and their pairs in the other location sub-sample because we did not observe them in the baseline period prior to the intervention. There are 190 treatment villages which received surgical masks as part of the intervention and 96 treatment villages which received cloth masks. Standard errors are in parentheses.

	Full	No active promotion	Mosques	Markets	Other Locations	Surgical Mask Villages	Cloth Mask Villages
			Proper Mask-Wearing				
Intervention Coefficient	0.288***<br>(0.012)	0.279***<br>(0.011)	0.370***<br>(0.016)	0.287***<br>(0.012)	0.251***<br>(0.012)	0.301***<br>(0.015)	0.256***<br>(0.019)
			Physical Distancing				
Intervention Coefficient	0.051***<br>(0.005)	0.056***<br>(0.005)	0.000<br>(0.000)	0.074***<br>(0.007)	0.068***<br>(0.006)	0.054***<br>(0.006)	0.044***<br>(0.011)
N villages	572	572	570	570	568	380	192

***Significant at the 1 percent level.
**Significant at the 5 percent level.
*Significant at the 10 percent level.

Table 2. Symptomatic seroprevalence, expressed in prevalence ratios. All regressions include an indicator for each control-intervention pair. The regressions "with baseline controls" include controls for baseline rates of proper mask wearing and baseline symptom rates. Baseline Symptom Rate is defined as the rate of surveyed individuals in a village who report symptoms coinciding with the WHO definition of a probable COVID-19 case. We assume that (1) all reported symptoms were acute onset, (2) all people live or work in an area with high risk of transmission of virus and (3) all people have been a contact of a probable or confirmed case of COVID-19 or are linked to a COVID-19 cluster. The analysis includes all people surveyed in the baseline household visits, excluding individuals that we did not collect midline or endline symptoms for, symptomatic individuals that we did not collect blood from, and individuals that we drew blood from but did not test their blood. The regressions excludes an additional 17,377 individuals in 34 villages because there are 0 people who are symptomatic-seropositive in their village pairs. To check robustness to the type of clustering, in panels 2 and 3 of Fig. 1, we show the histogram of effect sizes under "randomization inference" if we randomly reassign treatment within each pair of villages and then estimate our primary specification. We find that our estimated effect size is smaller than 7.0% of the simulated estimates with controls and 7.4% of the simulated estimates without controls (these are the corresponding p-values of the randomization inference *t*-test). Confidence Intervals are in brackets.

	Intervention Effect	Intervention Effect by Mask Type
	No Baseline Controls	
Intervention Prevalence Ratio	0.905**<br>[0.815, 0.995]	
Intervention Prevalence Ratio for Surgical Mask Villages		0.894*<br>[0.782, 1.007]
Intervention Prevalence Ratio for Cloth Mask Villages		0.925<br>[0.766, 1.083]
Average Symptomatic-Seroprevalence Rate in Paired Control Villages§	0.0076	0.0076
	With Baseline Controls	
Intervention Prevalence Ratio	0.905**<br>[0.815, 0.995]	
Intervention Prevalence Ratio for Surgical Mask Villages		0.889**<br>[0.780, 0.997]
Intervention Prevalence Ratio for Cloth Mask Villages		0.942<br>[0.781, 1.103]
N individuals	304,726	304,726
N villages	572	572

***Significant at the 1 percent level.
**Significant at the 5 percent level.
*Significant at the 10 percent level.

Table 3. WHO-defined COVID-19 symptoms, expressed in prevalence ratios. All regressions include an indicator for each control-intervention pair. The regressions "with baseline controls" include controls for baseline rates of proper mask wearing and baseline symptom rates. Baseline Symptom Rate is defined as the rate of surveyed individuals in a village who report symptoms coinciding with the WHO definition of a probable COVID-19 case. We assume that (1) all reported symptoms were acute onset, (2) all people live or work in an area with high risk of transmission of virus and (3) all people have been a contact of a probable or confirmed case of COVID-19 or are linked to a COVID-19 cluster. The analysis includes all people surveyed in the baseline household visits, excluding individuals that we did not collect midline or endline symptoms for. Confidence Intervals are in brackets.

§We report the mean rate of symptomatic seroprevalence at endline. This is not equivalent to the coefficient on the constant due to the inclusion of the pair indicators as controls.

	Intervention Effect	Intervention Effect by Mask Type
	No Baseline Controls	
Intervention Prevalence Ratio	0.885***<br>[0.834, 0.934]	
Intervention Prevalence Ratio for Surgical Mask Villages		0.865***<br>[0.803, 0.928]
Intervention Prevalence Ratio for Cloth Mask Villages		0.922*<br>[0.838, 1.005]
Average Symptomatic Rate in Paired Control Villages§	0.0860	0.0860
	With Baseline Controls	
Intervention Prevalence Ratio	0.884***<br>[0.834, 0.934]	
Intervention Prevalence Ratio for Surgical Mask Villages		0.874***<br>[0.809, 0.939]
Intervention Prevalence Ratio for Cloth Mask Villages		0.907**<br>[0.823, 0.991]
N individuals	321,948	321,948
N villages	572	572

***Significant at the 1 percent level.
**Significant at the 5 percent level.
*Significant at the 10 percent level.
§We report the mean rate of symptomatic status at endline. This is not equivalent to the coefficient on the constant due to the inclusion of the pair indicators as controls.

Table 4. Symptomatic seroprevalence by age groups and mask type, expressed in prevalence ratios. All regression include an indicator for each control-intervention pair. The regressions include controls for baseline rates of mask-wearing and baseline symptom rates. Baseline Symptom Rate is defined as the rate of surveyed individuals in a village who report symptoms coinciding with the WHO definition of a probable COVID-19 case. We assume that (1) all reported symptoms were acute onset, (2) all people live or work in an area with high risk of transmission of virus and (3) all people have been a contact of a probable or confirmed case of COVID-19 or are linked to a COVID-19 cluster.The analysis in the top panel utilizes the pre-registered sample, equivalent to Table 2; it includes all people surveyed in the baseline household visits, excluding individuals that we did not collect midline or endline symptoms for, symptomatic individuals that we did not collect blood from, and individuals that we drew blood from but did not test their blood. The analysis in the bottom panel replicates the regressions in the top panel, but imputes the seropositivity of individuals for who we did not draw blood. For symptomatic individuals we did not draw blood from, we simulate their symptomatic-seroprevalence status by using the average rate of conditional seropositivity among all symptomatic individuals. This analysis includes all people surveyed in the baseline household visits, excluding individuals that we did not collect midline or endline symptoms for. Confidence Intervals are in brackets.

	All	< 40 Y.O.	40-49 Y.O.	50-59 Y.O.	≥ 60 Y.O.
	Pre-Registered Sample: Drop Individuals Without Blood Draws				
Intervention Prevalence Ratio for Surgical Mask Villages	0.889** [0.780, 0.997]	0.967 [0.834, 1.100]	1.009 [0.817, 1.200]	0.772** [0.595, 0.949]	0.647*** [0.448, 0.845]
Intervention Prevalence Ratio for Cloth Mask Villages	0.942 [0.781, 1.103]	1.058 [0.870, 1.247]	0.713** [0.459, 0.967]	0.838 [0.524, 1.153]	1.084 [0.769, 1.399]
Avg. Symptomatic-Seroprevalence in Paired Control Villages§	0.0076	0.0055	0.0095	0.0108	0.0104
N Individuals	287,349	146,306	35,839	24,086	27,943
N Villages	538	480	384	348	360
	Imputing Symptomatic-Seroprevalence for Missing Blood Draws				
Intervention Prevalence Ratio for Surgical Mask Villages	0.873*** [0.801, 0.945]	0.917* [0.829, 1.005]	0.975 [0.862, 1.088]	0.815*** [0.688, 0.942]	0.701*** [0.577, 0.824]
Intervention Prevalence Ratio for Cloth Mask Villages	0.890** [0.787, 0.993]	0.861*** [0.758, 0.965]	0.838** [0.678, 0.998]	1.153 [0.970, 1.336]	0.792** [0.601, 0.983]
Avg. Symptomatic-Seroprevalence in Paired Control Villages§	0.0189	0.0152	0.0226	0.0229	0.0251
N Individuals	321,383	177,708	51,676	37,340	43,431
N Villages	570	566	528	504	534

***Significant at the 1 percent level.
**Significant at the 5 percent level.
*Significant at the 10 percent level.
§We report the mean rate of symptomatic seroprevalence at endline. This is not equivalent to the coefficient on the constant due to the inclusion of the pair indicators as controls.
