# Supplementary material for: Impact of community masking on COVID-19: A cluster-randomized trial in Bangladesh
Source: Science. 2022 Jan 14;375(6577):eabi9069. doi: 10.1126/science.abi9069 (PMC9036942; doi:10.1126/science.abi9069)
Supplement: Supplementary file 2 — Figs. S1 to S6 Tables S1 to S37 Appendices References (79–81) [file science.abi9069_sm.pdf]

Science
AAAS

Supplementary Materials for

Impact of community masking on COVID-19: A cluster-randomized trial in Bangladesh

Jason Abaluck *et al.*

Corresponding authors: Jason Abaluck, jason.abaluck@yale.edu; Ahmed Mushfiq Mobarak, ahmed.mobarak@yale.edu

Science **375**, eabi9069 (2022)
DOI: 10.1126/science.abi9069

The PDF file includes:

Figs. S1 to S6
Tables S1 to S37
Appendices
References

Other Supplementary Material for this manuscript includes the following:

MDAR Reproducibility Checklist

A List of Supplementary Materials

Appendix Figures and Tables

Fig. S1. Schematic of Cross-Randomizations

Fig. S2. Randomization Inference on Symptomatic Seroprevalence and Symptoms

Table S1. Enrollment and Consent (Individual-Level)

Table S2. Enrollment and Consent (Household-Level)

Table S3. Endline Blood Collection Consent Rates by Demographic Characteristics

Table S4. Balance Tests (Village-Level)

Table S5. Mask-Wearing and Physical Distancing, without Controlling for Baseline Variables

Table S6. Number of People Observed

Table S7. Symptomatic Seroprevalence

Table S8. WHO-defined COVID-19 Symptoms

Table S9. WHO-defined COVID-19 Symptoms (Robustness Check)

Table S10. Pilot Analyses of Mask Wearing

Table S11. Subgroup Analyses of Mask-Wearing

Table S12. Symptomatic Seroprevalence by 10-Year Age Groups

Table S13. WHO-Defined COVID-19 Symptoms by Age Groups

Table S14. WHO-Defined COVID-19 Symptoms by 10-Year Age Groups

Table S15. WHO-Defined COVID-19 Symptoms by Age Groups, Expressed in Prevalence Ratios

Appendices

A. List of Supplementary Materials

B. Sample Size

C. Pairwise Randomization Procedure

D. Cross-Randomization Procedure

E. Scoping and Recruitment

F. Details on Mask Materials and Design

G. Details on Surveillance

H. Antibody Testing

I. Impact of Masks on Symptoms, Seroprevalence, and Seroconversions

J. Behavioral Mechanisms

K. Statistical Analysis

L. Additional Balance Tests

M. Persistence of Mask-Wearing Behavior

N. Imputed Symptomatic Seroprevalence

O. Variation of Effects

P. Additional Preregistered Specifications

Q. Intervention Cost and Benefit Estimates

R. Polling Policy Makers

Tables and Figures Referenced in Appendices

Fig. S3. Village-Level Cross Randomizations

Fig. S4. Household-Level Cross Randomizations

Fig. S5. Persistence of Mask-Wearing

Fig. S6. Variation of Effect within Treatment Arms

Table S16. Village-Level Cross Randomizations

Table S17. Household-Level Cross-Randomizations

Table S18. Balance Tests (Individual-Level)

Table S19. Additional Balance Tests (Individual-Level)

Table S20. Additional Balance Tests (Individual-Level, After Sample Selection)

Table S21. Symptomatic Seroprevalence (With Controls and Additional Sample Selection)

Table S22. Persistence of Mask-Wearing

Table S23. Mask-Wearing and Physical Distancing by Wave, Controlling for Baseline Variables

Table S24. Symptomatic-Seroprevalence by Wave, With Baseline Controls

Table S25. Endline Blood Collection Consent Rates by Wave

Table S26. Symptomatic Seroprevalence by Age Groups, With Baseline Controls, Expressed in Prevalence Ratios

Table S27. Symptomatic Seroprevalence by Sex with Baseline Controls, Expressed in Prevalence Ratios

Table S28. WHO-Defined COVID Symptoms by Sex, Expressed in Prevalence Ratios

Table S29. Symptomatic Seroprevalence & COVID Symptoms by Mask Wearing & Physical Distancing

Table S30. IV Regressions

Table S31. Calculation of Number Needed to Treat and Cost per Life Saved

Table S32. What do you think was the increase in mask-wearing as a result of household mask distribution and mask promotion in the community?

Table S33. What do you think was the additional effect of mask promoters reminding people to wear masks?

Table S34. Do you think text message reminders to wear masks further increased mask-wearing?

Table S35. How do you think mask distribution and promotion affected physical distancing?

Table S36. Do you think incentive payments to village leaders further increased mask-wearing?

Table S37. Do you think verbal commitments and signage to wearing masks further increased mask-wearing?

Figure S1: Schematic of Cross-Randomizations

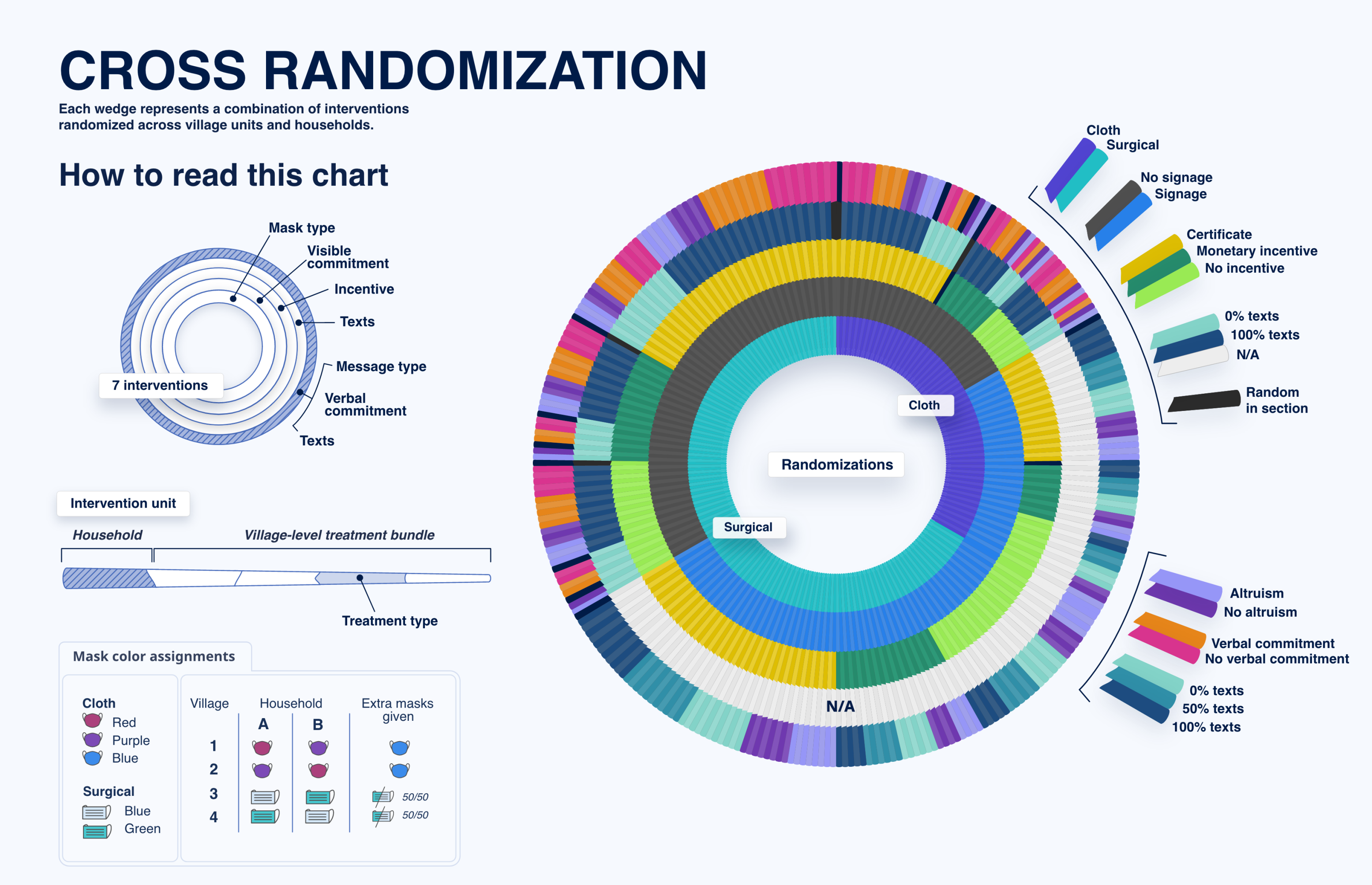

Notes: Each color represents a village-level or household-level randomization, with inner circles representing village-level randomizations and outer circles representing household level randomization. Different tones of the same hue represent the control or treatment status for each randomization. The fraction of villages receiving each randomization is proportional to the area of each concentric circle. The "Colors" box in the bottom left shows the color of masks used to denote households that were assigned to the control or treatment status of the household-level randomization.

Table S1: Enrollment and Consent (Individual-Level)

	Treatment Villages	Control Villages	Total
Number of People Consented to Baseline Household Visits	178,322	163,861	342,183
Number of People Reached for Symptom Collection in the Midline and/or Endline Visits	174,776	161,234	336,010
Number of People with WHO-defined COVID-19 Symptoms	13,307	13,853	27,160
Number of Symptomatic Endline Blood Samples Collected	5,345	5,445	10,790
Number of Symptomatic Endline Blood Samples Tested	4,714	4,798	9,512

All counts provided are at the individual level.
Table S2 shows household approached separately from households that consented to our baseline survey.

Table S2: Enrollment and Consent (Household-Level)

	Treatment Villages	Control Villages	Total
Number of HHs Approached in Baseline Household Visit	68,514	65,536	134,050
Number of HHs that Consented to Baseline Household Visits	64,851	60,202	125,053
Number of HHs Reached for Symptom Collection in the Midline and/or Endline Visits	63,489	59,163	122,652

All counts provided are at the household level.

Table S3: Endline Blood Collection Consent Rates by Demographic Characteristics

	Treatment	Control	Total
Total	40.2%	39.3%	39.7%
		By Sex	
Female	40.2%	39.3%	39.7%
Male	40.2%	39.3%	39.7%
		By Age Group	
< 40 Y.O.	40.4%	38.7%	39.5%
40-49 Y.O.	39.9%	40.0%	40.0%
50-59 Y.O.	41.0%	40.2%	40.6%
$\geq$ 60 Y.O.	39.2%	39.3%	39.3%

Consent rates are defined as the ratio of the number of individuals we successfully drew blood from to the number of eligible symptomatic individuals; those who met the WHO definition of a probable COVID-19 case.

Table S4: Balance Tests (Village-Level)

	Baseline Symptomatic Seroprevalence Rate	Baseline WHO-Defined COVID-19 Symptoms	Baseline Mask-Wearing Rate
	Summary Statistics		
Intervention Rate	0.00022	0.02687	0.12297
Control Rate	0.00028	0.02540	0.12430
	Balance Tests		
Intervention Coefficient	-0.00005<br>(0.00007)	0.00086<br>(0.00173)	0.00069<br>(0.00549)
N villages	572	572	572
F		0.18	
Joint-Test Prob > F		0.8355	

Standard errors are in parentheses.
*** Significant at the 1 percent level. ** Significant at the 5 percent level. * Significant at the 10 percent level.
The baseline rate of mask-wearing was measured through observation over a 1 week period, defined as the rate of those observed who wear a mask or face covering that covers the nose and mouth.

Table S5: Mask-Wearing and Physical Distancing, without Controlling for Baseline Variables

	Full	No Active Promotion	Mosques	Markets	Other Locations	Surgical Mask Villages	Cloth Mask Villages
Proper Mask-Wearing							
Intervention Coefficient	0.288***	0.279***	0.371***	0.288***	0.252***	0.302***	0.258***
	(0.012)	(0.012)	(0.016)	(0.012)	(0.012)	(0.014)	(0.020)
Average Mask-Wearing Rate in Paired Control Villages§	0.133	0.134	0.123	0.120	0.146	0.129	0.143
Physical Distancing							
Intervention Coefficient	0.050***	0.056***	0.000	0.073***	0.067***	0.053***	0.044***
	(0.005)	(0.005)	(0.000)	(0.007)	(0.007)	(0.006)	(0.011)
Average Distancing Rate in Paired Control Villages§	0.241	0.253	0.000	0.291	0.311	0.229	0.268
N villages	572	572	570	570	568	380	192

Standard errors are in parentheses.
*** Significant at the 1 percent level. ** Significant at the 5 percent level. * Significant at the 10 percent level.
All regressions include an indicator for each control-intervention pair.
§We report the mean rate of proper mask-wearing among the control villages during the baseline observation. This is not equivalent to the coefficient on the constant due to the inclusion of the pair indicators as controls.
'No Active Promotion" refers to any time that surveillance was conducted while promotion was not actively occurring (regardless of the week of the intervention). This excludes surveillance during the Friday Jumma Prayers in the mosque, when promoters were present and actively encouraged mask wearing.
"Other Locations" include tea stalls, at the entrance of the restaurant as patrons enter, and the main road to enter the village.
"Surgical Villages" refer to all treatment villages which received surgical masks as part of the intervention, and their control pairs. "Cloth Villages" refer to all treatment villages which received cloth masks as part of the intervention, and their control pairs. These samples include surveillance from all available locations, equivalent to the to the column labeled "Full", but run separately for each subgroup.
Of the 572 villages included in the analyses sample, we exclude an additional village and its pair in the mosque and market sub-samples, and two villages and their pairs in the other location sub-sample because we did not observe them in the baseline period prior to the intervention. There are 190 treatment villages which received surgical masks as part of the intervention and 96 treatment villages which received cloth masks.

Table S6: Number of People Observed

	Full	No Active Promotion	Mosques	Markets	Other Locations	Surgical Mask Villages	Cloth Mask Villages
No Baseline Control							
Intervention Coefficient	-31<br>(51)	-53<br>(45)	35<br>(24)	-20<br>(17)	-46**<br>(23)	-9<br>(63)	-75<br>(85)
Avg. Number People Observed in Paired Control Villages§	2820	2682	580	882	1358	2914	2635
With Baseline Control							
Intervention Coefficient	-43<br>(45)	-64<br>(40)	23<br>(20)	-18<br>(15)	-53**<br>(21)	-37<br>(58)	-45<br>(76)
N villages	572	572	570	570	568	380	192

Standard errors are in parentheses.
*** Significant at the 1 percent level. ** Significant at the 5 percent level. * Significant at the 10 percent level.
All regressions include an indicator for each control-intervention pair. The regressions ”with baseline control” include controls for the number of people observed in the baseline visit.
§We report the average number of people observed among the control villages during the baseline observation. This is not equivalent to the coefficient on the constant due to the inclusion of the pair indicators as controls.
“No Active Promotion” refers to any time that surveillance was conducted while promotion was not actively occurring (regardless of the week of the intervention). This excludes surveillance during the Friday Jumma Prayers in the mosque, when promoters were present and actively encouraged mask wearing.
”Other Locations” include tea stalls, at the entrance of the restaurant as patrons enter, and the main road to enter the village.
“Surgical Villages” refer to all treatment villages which received surgical masks as part of the intervention, and their control pairs. “Cloth Villages” refer to all treatment villages which received cloth masks as part of the intervention, and their control pairs. These samples include surveillance from all available locations, equivalent to the to the column labeled “Full”, but run separately for each subgroup.
Of the 572 villages included in the analyses sample, we exclude an additional village and its pair in the mosque and market sub-samples, and two villages and their pairs in the other location sub-sample because we did not observe them in the baseline period prior to the intervention. There are 190 treatment villages which received surgical masks as part of the intervention and 96 treatment villages which received cloth masks.

Table S7: Symptomatic Seroprevalence

	Intervention Effect	Intervention Effect by Mask Type
No Baseline Controls		
Intervention Coefficient	-0.0007**	
	(0.0003)	
Intervention Coefficient for Surgical Mask Villages		-0.0008*
		(0.0004)
Intervention Coefficient for Cloth Mask Villages		-0.0005
		(0.0005)
Average Symptomatic Seroprevalence Rate in Paired Control Villages§	0.0076	0.0076
With Baseline Controls		
Intervention Coefficient	-0.0007**	
	(0.0003)	
Intervention Coefficient for Surgical Mask Villages		-0.0009**
		(0.0004)
Intervention Coefficient for Cloth Mask Villages		-0.0003
		(0.0005)
N individuals	304,726	304,726
N villages	572	572

Standard errors are in parentheses.
*** Significant at the 1 percent level. ** Significant at the 5 percent level. * Significant at the 10 percent level.
All regressions include an indicator for each control-intervention pair. The regressions "with baseline controls" include controls for baseline rates of proper mask wearing and baseline symptom rates.
Baseline Symptom Rate is defined as the rate of surveyed individuals in a village who report symptoms coinciding with the WHO definition of a probable COVID-19 case. We assume that (1) all reported symptoms were acute onset, (2) all people live or work in an area with high risk of transmission of virus and (3) all people have been a contact of a probable or confirmed case of COVID-19 or are linked to a COVID-19 cluster. newline §We report the mean rate of symptomatic seroprevalence at endline. This is not equivalent to the coefficient on the constant due to the inclusion of the pair indicators as controls.
The analysis includes all people surveyed in the baseline household visits, excluding individuals that we did not collect midline or endline symptoms for, symptomatic individuals that we did not collect blood from, and individuals that we drew blood from but did not test their blood.

Figure S2: Randomization Inference on Symptomatic Seroprevalence and Symptoms

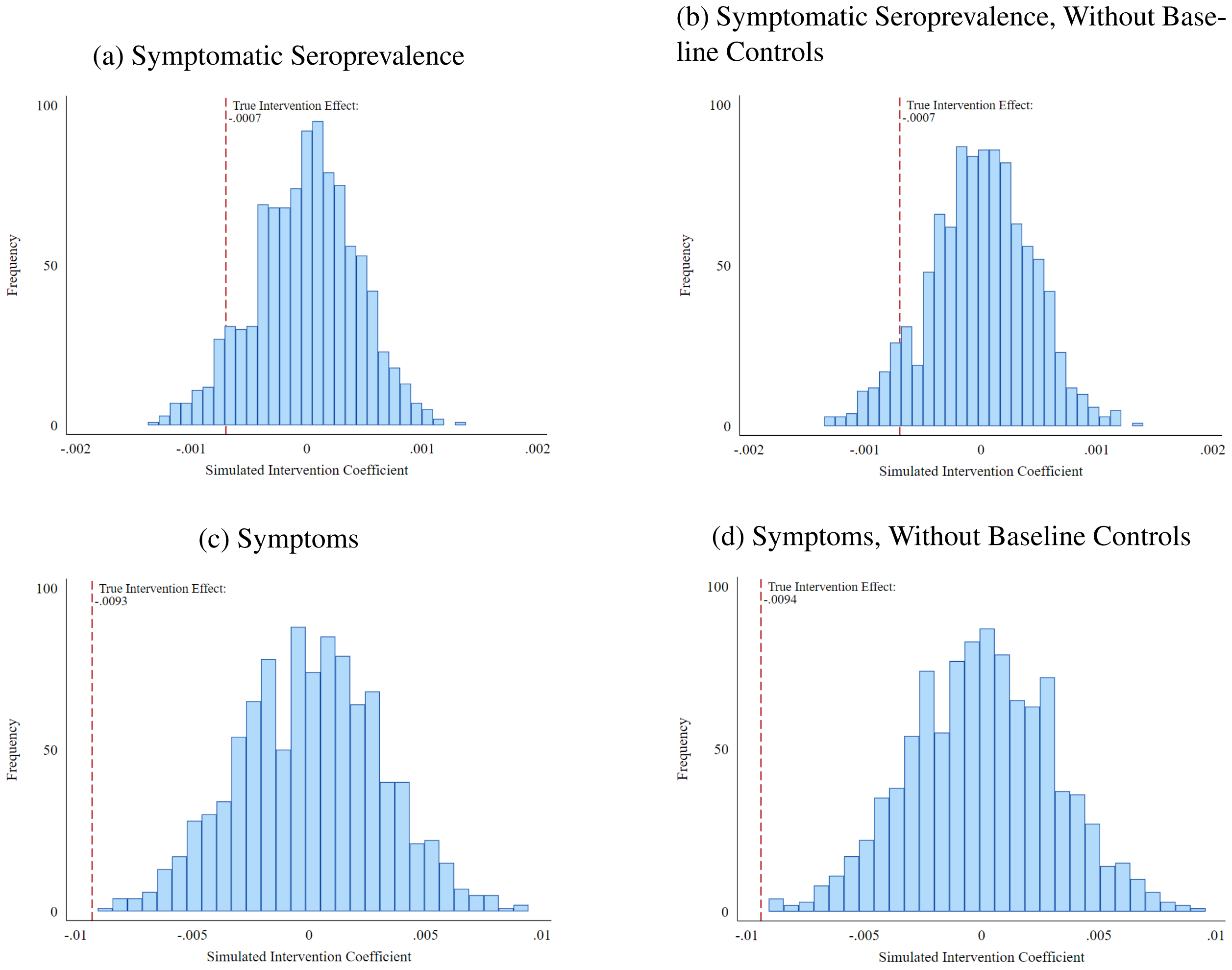

The histograms are generated by plotting the frequency the coefficient on the intervention under 1,000 imputations of randomly assigning the treatment/control status within each village-pair. The regressions used to generate the intervention coefficient in panel (a) and (b) are equivalent to those in Table S7, top and bottom panel, respectively. The regressions used in panel (c) and (d) are equivalent to those in Table S8, top and bottom panel, respectively.
The one-sided *p*-values for each panel is as follows:
(a) 0.070
(b) 0.074
(c) 0.000
(d) 0.000

Table S8: WHO-defined COVID-19 Symptoms

	Intervention Effect	Intervention Effect by Mask Type
	No Baseline Controls	
Intervention Coefficient	-0.0094*** (0.0022)	
Intervention Coefficient for Surgical Mask Villages		-0.0103*** (0.0028)
Intervention Coefficient for Cloth Mask Villages		-0.0074** (0.0034)
Average Symptomatic Rate in Paired Control Villages§	0.0860	0.0860
	With Baseline Controls	
Intervention Coefficient	-0.0093*** (0.0021)	
Intervention Coefficient for Surgical Mask Villages		-0.0111*** (0.0028)
Intervention Coefficient for Cloth Mask Villages		-0.0058* (0.0034)
N individuals	321,948	321,948
N villages	572	572

Standard errors are in parentheses.
*** Significant at the 1 percent level. ** Significant at the 5 percent level. * Significant at the 10 percent level.
All regressions include an indicator for each control-intervention pair. The regressions "with baseline controls" include controls for baseline rates of proper mask wearing and baseline symptom rates.
Baseline Symptom Rate is defined as the rate of surveyed individuals in a village who report symptoms coinciding with the WHO definition of a probable COVID-19 case. We assume that (1) all reported symptoms were acute onset, (2) all people live or work in an area with high risk of transmission of virus and (3) all people have been a contact of a probable or confirmed case of COVID-19 or are linked to a COVID-19 cluster.
§We report the mean rate of symptomatic seroprevalence at endline. This is not equivalent to the coefficient on the constant due to the inclusion of the pair indicators as controls.
The analysis includes all people surveyed in the baseline household visits, excluding individuals that we did not collect midline or endline symptoms for.

Table S9: WHO-defined COVID-19 Symptoms (Robustness Check)

	Intervention Effect	Intervention Effect by Mask Type
	No Baseline Controls	
Intervention Coefficient	-0.0031***	
	(0.0011)	
Intervention Coefficient for Surgical Mask Villages		-0.0048***
		(0.0015)
Intervention Coefficient for Cloth Mask Villages		0.0002
		(0.0017)
Average Symptomatic Rate in Paired Control Villages§	0.0329	0.0329
	With Baseline Controls	
Intervention Coefficient	-0.0030***	
	(0.0011)	
Intervention Coefficient for Surgical Mask Villages		-0.0051***
		(0.0015)
Intervention Coefficient for Cloth Mask Villages		0.0010
		(0.0017)
N individuals	304,726	304,726
N villages	572	572

Standard errors are in parentheses.
*** Significant at the 1 percent level. ** Significant at the 5 percent level. * Significant at the 10 percent level.
All regressions include an indicator for each control-intervention pair. The regressions "with baseline controls" include controls for baseline rates of proper mask wearing and baseline symptom rates.
Baseline Symptom Rate is defined as the rate of surveyed individuals in a village who report symptoms coinciding with the WHO definition of a probable COVID-19 case. We assume that (1) all reported symptoms were acute onset, (2) all people live or work in an area with high risk of transmission of virus and (3) all people have been a contact of a probable or confirmed case of COVID-19 or are linked to a COVID-19 cluster.
§We report the mean rate of symptomatic seroprevalence at endline. This is not equivalent to the coefficient on the constant due to the inclusion of the pair indicators as controls.
The analysis includes all people surveyed in the baseline household visits, excluding individuals that we did not collect midline or endline symptoms for, symptomatic individuals that we did not collect blood from, and individuals that we drew blood from but did not test their blood.

Table S10: Pilot Analyses of Mask Wearing

	Main Intervention	Pilot 1	Pilot 2	Pilot 1	Pilot 2
No Baseline Controls					
Intervention Coefficient	0.288*** (0.012)	0.109 [-0.165, 0.306]	0.284 [0.105, 0.410]		
Difference from Main Intervention				-0.181** (0.092)	-0.005 (0.058)
Average Control Mask Wearing Rate§	0.133	0.129	0.095		
With Baseline Controls					
Intervention Effect	0.288*** (0.012)	0.096 [-0.120, 0.302]	0.341 [0.099, 0.489]		
Difference from Main Intervention				-0.189** (0.073)	0.022 (0.053)
N villages	572	10	10	592	592

Standard errors are in parentheses. Confidence intervals are in brackets, computed using wild bootstrap.
*** Significant at the 1 percent level. ** Significant at the 5 percent level. * Significant at the 10 percent level.
The regressions ”with baseline controls” include controls for baseline rates of mask-wearing.
The first column reports the results of our main intervention; equivalent to the results in Table 1, using full surveillance data.
§We report the mean rate of mask-wearing among the control villages during the baseline observation. This is not equivalent to the coefficient on the constant due to the inclusion of the pair indicators as controls.

Table S11: Subgroup Analyses of Mask-Wearing

	Female Only	Male Only	Above Median	Below Median
No Baseline Controls				
Intervention Coefficient	0.225***	0.271***	0.247***	0.346***
	(0.013)	(0.013)	(0.018)	(0.022)
Average Control Mask-Wearing Rate§	0.313	0.116	0.175	0.087
With Baseline Controls				
Intervention Coefficient	0.225***	0.271***	0.247***	0.350***
	(0.013)	(0.013)	(0.019)	(0.022)
N villages	566	566	200	200

Standard errors are in parentheses.
*** Significant at the 1 percent level. ** Significant at the 5 percent level. * Significant at the 10 percent level.
All regressions include an indicator for each control-intervention pair. The baseline control regressions include controls for baseline rates of mask-wearing and baseline symptom rates. For the gender subgroup analyses, the baseline symptom rate and baseline mask-wearing rate was defined across all individuals, not just those among females and males, respectively.
Baseline Symptom Rate is defined as the rate of surveyed individuals in a village who report symptoms coinciding with the WHO definition of a probable COVID-19 case. We assume that (1) all reported symptoms were acute onset, (2) all people live or work in an area with high risk of transmission of virus and (3) all people have been a contact of a probable or confirmed case of COVID-19 or are linked to a COVID-19 cluster. §We report the mean rate of proper mask-wearing among the control villages during the baseline observation. This is not equivalent to the coefficient on the constant due to the inclusion of the pair indicators as controls.
The sex-specific subgroup is run on all locations except mosques because no females were observed at mosques. The sex-specific samples excludes 6 villages because of lack of data. The above-median and below-median samples includes 85 singleton observations which were dropped.

Table S12: Symptomatic Seroprevalence by 10-Year Age Groups

	All	18-29 Y.O.	30-39 Y.O.	40-49 Y.O.	50-59 Y.O.	60-69 Y.O.	$\geq$ 70 Y.O.
			No Baseline Controls				
Intervention Coefficient	-0.0007**	-0.0004	0.0007	-0.0008	-0.0021***	-0.0020**	-0.0018
	(0.0003)	(0.0003)	(0.0005)	(0.0007)	(0.0008)	(0.0010)	(0.0012)
Avg. Symptomatic Seroprevalence in Paired Control Vill.§	0.0076	0.0066	0.0100	0.0136	0.0175	0.0203	0.0270
			With Baseline Controls				
Intervention Coefficient	-0.0007**	-0.0004	0.0007	-0.0008	-0.0021***	-0.0019**	-0.0019
	(0.0003)	(0.0003)	(0.0005)	(0.0007)	(0.0008)	(0.0010)	(0.0012)
N Individuals	304,726	101,137	69,717	51,727	38,996	27,625	15,524
N Villages	572	572	572	572	572	572	572

Standard errors are in parentheses.
*** Significant at the 1 percent level. ** Significant at the 5 percent level. * Significant at the 10 percent level.
All regressions include an indicator for each control-intervention pair. The regressions "with baseline controls" include controls for baseline rates of proper mask wearing and baseline symptom rates.
Baseline Symptom Rate is defined as the rate of surveyed individuals in a village who report symptoms coinciding with the WHO definition of a probable COVID-19 case. We assume that (1) all reported symptoms were acute onset, (2) all people live or work in an area with high risk of transmission of virus and (3) all people have been a contact of a probable or confirmed case of COVID-19 or are linked to a COVID-19 cluster.
§We report the mean rate of symptomatic seroprevalence at endline. This is not equivalent to the coefficient on the constant due to the inclusion of the pair indicators as controls.
The analysis includes all people surveyed in the baseline household visits, excluding individuals that we did not collect midline or endline symptoms for, symptomatic individuals that we did not collect blood from, and individuals that we drew blood from but did not test their blood.

Table S13: WHO-Defined COVID-19 Symptoms by Age Groups

	All	< 40 Y.O.	40-49 Y.O.	50-69 Y.O.	$\geq$ 60 Y.O.
		No Baseline Controls			
Intervention Coefficient<br>for Surgical Mask Villages	-0.0103***<br>(0.0028)	-0.0085***<br>(0.0028)	-0.0091***<br>(0.0035)	-0.0122***<br>(0.0039)	-0.0172***<br>(0.0043)
Intervention Coefficient<br>for Cloth Mask Villages	-0.0074**<br>(0.0034)	-0.0057**<br>(0.0027)	-0.0010<br>(0.0051)	-0.0161***<br>(0.0059)	-0.0112*<br>(0.0058)
Average Symptomatic Rate<br>in Paired Control Villages§	0.0860	0.0717	0.0983	0.1060	0.1080
		With Baseline Controls			
Intervention Coefficient<br>for Surgical Mask Villages	-0.0111***<br>(0.0028)	-0.0093***<br>(0.0028)	-0.0097***<br>(0.0035)	-0.0127***<br>(0.0038)	-0.0180***<br>(0.0042)
Intervention Coefficient<br>for Cloth Mask Villages	-0.0058*<br>(0.0034)	-0.0045<br>(0.0027)	0.0010<br>(0.0050)	-0.0139**<br>(0.0058)	-0.0087<br>(0.0058)
N Individuals	321,948	178,881	55,182	41,683	46,202
N Villages	572	572	572	572	572

Standard errors are in parentheses.
*** Significant at the 1 percent level. ** Significant at the 5 percent level. * Significant at the 10 percent level.
All regressions include an indicator for each control-intervention pair. The regressions “with baseline controls” include controls for baseline rates of proper mask wearing and baseline symptom rates.
Baseline Symptom Rate is defined as the rate of surveyed individuals in a village who report symptoms coinciding with the WHO definition of a probable COVID-19 case. We assume that (1) all reported symptoms were acute onset, (2) all people live or work in an area with high risk of transmission of virus and (3) all people have been a contact of a probable or confirmed case of COVID-19 or are linked to a COVID-19 cluster.
§We report the mean rate of symptomatic seroprevalence at endline. This is not equivalent to the coefficient on the constant due to the inclusion of the pair indicators as controls.
The analysis includes all people surveyed in the baseline household visits, excluding individuals that we did not collect midline or endline symptoms for.

Table S14: WHO-Defined COVID-19 Symptoms by 10-Year Age Groups

	All	18-29 Y.O.	30-39 Y.O.	40-49 Y.O.	50-59 Y.O.	60-69 Y.O.	$\geq$ 70 Y.O.
				No Baseline Controls			
Intervention Coefficient	-0.0094***	-0.0079***	-0.0068**	-0.0064**	-0.0135***	-0.0122***	-0.0192***
	(0.0022)	(0.0020)	(0.0027)	(0.0029)	(0.0033)	(0.0037)	(0.0045)
Avg Symptomatic Rate in Paired Control Vill.§	0.0860	0.0607	0.0872	0.0983	0.1064	0.1083	0.1123
				With Baseline Controls			
Intervention Coefficient	-0.0093***	-0.0080***	-0.0067**	-0.0061**	-0.0131***	-0.0119***	-0.0188***
	(0.0021)	(0.0020)	(0.0026)	(0.0029)	(0.0032)	(0.0036)	(0.0045)
N Individuals	321,948	105,163	73,718	55,182	41,683	29,616	16,586
N Villages	572	572	572	572	572	572	572

Standard errors are in parentheses.
*** Significant at the 1 percent level. ** Significant at the 5 percent level. * Significant at the 10 percent level.
All regressions include an indicator for each control-intervention pair. The regressions "with baseline controls" include controls for baseline rates of proper mask wearing and baseline symptom rates.
Baseline Symptom Rate is defined as the rate of surveyed individuals in a village who report symptoms coinciding with the WHO definition of a probable COVID-19 case. We assume that (1) all reported symptoms were acute onset, (2) all people live or work in an area with high risk of transmission of virus and (3) all people have been a contact of a probable or confirmed case of COVID-19 or are linked to a COVID-19 cluster.
§We report the mean rate of symptomatic seroprevalence at endline. This is not equivalent to the coefficient on the constant due to the inclusion of the pair indicators as controls.
The analysis includes all people surveyed in the baseline household visits, excluding individuals that we did not collect midline or endline symptoms for.

Table S15: WHO-Defined COVID-19 Symptoms by Age Groups, Expressed in Prevalence Ratios

	All	< 40 Y.O.	40-49 Y.O.	50-59 Y.O.	≥ 60 Y.O.
			No Baseline Controls		
Intervention Coefficient<br>for Surgical Mask Villages	0.874***<br>[0.809, 0.939]	0.876***<br>[0.800, 0.953]	0.903***<br>[0.831, 0.975]	0.879***<br>[0.805, 0.953]	0.829***<br>[0.750, 0.908]
Intervention Coefficient<br>for Cloth Mask Villages	0.907**<br>[0.823, 0.991]	0.913**<br>[0.835, 0.991]	0.989<br>[0.886, 1.093]	0.832***<br>[0.714, 0.950]	0.888*<br>[0.775, 1.002]
Average Symptomatic-Seroprevalence<br>in Paired Control Villages§	0.0860	0.0717	0.0983	0.1060	0.1080
			With Baseline Controls		
Intervention Coefficient<br>for Surgical Mask Villages	0.865***<br>[0.803, 0.928]	0.866***<br>[0.792, 0.941]	0.897***<br>[0.827, 0.967]	0.870***<br>[0.797, 0.943]	0.823***<br>[0.748, 0.899]
Intervention Coefficient<br>for Cloth Mask Villages	0.922*<br>[0.838, 1.005]	0.921*<br>[0.842, 1.001]	1.007<br>[0.905, 1.108]	0.853**<br>[0.737, 0.970]	0.907<br>[0.795, 1.020]
N Individuals	321,948	178,881	55,182	41,569	46,071
N Villages	572	572	572	570	570

Confidence Intervals are in brackets.
*** Significant at the 1 percent level. ** Significant at the 5 percent level. * Significant at the 10 percent level.
All regressions include an indicator for each control-intervention pair. The regressions "with baseline controls" include controls for baseline rates of proper mask wearing and baseline symptom rates.
Baseline Symptom Rate is defined as the rate of surveyed individuals in a village who report symptoms coinciding with the WHO definition of a probable COVID-19 case. We assume that (1) all reported symptoms were acute onset, (2) all people live or work in an area with high risk of transmission of virus and (3) all people have been a contact of a probable or confirmed case of COVID-19 or are linked to a COVID-19 cluster.
§We report the mean rate of symptomatic seroprevalence at endline. This is not equivalent to the coefficient on the constant due to the inclusion of the pair indicators as controls.
The analysis includes all people surveyed in the baseline household visits, excluding individuals that we did not collect midline or endline symptoms for.

B Sample Size

To determine the necessary sample size for a cluster randomized trial, we used equation 5 for binary outcomes from Rutterford, et al (*79*). We rearranged the equation to solve for delta, the clinically relevant difference in reduction of symptomatic seropositivity. This allowed us to determine the optimum power we could achieve within budget and logistical constraints. By enrolling 600 villages with an anticipated number of 250 households per village and two eligible persons per household, we estimated our per-arm sample size would be 150,000 adults. We assumed P1=P2 and conservatively estimated the proportion of seropositivity at endline to be 9% with 4% attributed to the study period. We estimated an intercluster correlation coefficient of 0.02. This gave us a delta of 8.29E-03. Dividing by P gave us a minimum detectable effect of 9.2%. To determine the number of blood tests needed, we estimated that 12% of people enrolled would develop COVID-like symptoms over the study period and that one-third these individuals would have a SARS-CoV-2 infection. This gave us a target of 36,000 blood tests.

C Pairwise Randomization Procedure

To develop the sample frame, Innovations for Poverty Action (IPA) Bangladesh selected 1,000 rural and peri-urban unions out of 4,500 unions in Bangladesh. We excluded Dhaka district, because of high initial seroprevalence, and three hill districts, because of the logistical difficulties in accessing the region. We also dropped remote coastal districts where population density is low. The final sampling frame of 1000 unions were located in 40 different districts (*zillas*) (out of 64) and 144 sub-districts (*upazilas*) (out of 485).

We used a pairwise randomization to select 300 intervention and 300 control unions within the same sub-districts. This randomization procedure was designed to pair unions that were similar in terms of (limited) COVID-19 case data, population size, and population density. Each union consists of roughly 80,000 people, or around 80 villages. Surveyors blind to treatment assignment followed a scoping protocol (Appendix E) to identify the union's largest market and co-located

village. Field staff sought consent for a baseline survey in every household in every selected village; in intervention villages every adult in consenting households was given a mask. Some unions are very small so to avoid spillover effects, so we selected only on village per union and we ensured that selected villages were at least 2 km apart. Treatment and control unions were scattered throughout the country (Figure 1).

Villages were assigned to strata as follows:

1. We began with 1,000 villages in 1,000 separate unions to ensure sufficient geographic distance to prevent spillovers (Bangladesh is divided into 4,562 unions).

2. We collected these unions into "units", defined as the intersection of upazila x (above/below) median population x case trajectory, where above/below median population was a 0-1 indicator for whether the union had above-median population for that upazila and case trajectory takes the values -1, 0, 1 depending on whether the cases per 1,000 are decreasing, flat or increasing. We assessed cases per person using data provided to us from the Bangladeshi government for the periods June 27th-July 10th and July 11th-July 24th, 2020.

3. If a unit contained an odd number of unions, we randomly dropped one union.

4. We then sorted unions by "cases per person" based on data from July 11-24, 2020 and created pairs using adjacent unions in this sort order. We randomly kept 300 such pairs.

5. We randomly assigned one union in each pair to be the intervention union.

6. We then tested for balance with respect to cases, cases per population, and density.

7. Finally, we repeated this entire procedure 50 times, selecting the seed that minimized the maximum of the absolute value of the t-stat of the balance tests with respect to case trajectory and cases per person.

D Cross-Randomization Procedure

Villages were assigned to village-level cross-randomizations as follows:

1. We began with the 300 union-pairs (600 villages total) identified in the pairwise randomization procedure, and limited to only the villages in the intervention group.
2. Using a random number generator, we ordered the villages, and assigned the first 1/3 of the intervention villages to be distributed cloth masks and 2/3 to be distributed surgical masks.
3. Within the mask-type randomization, we randomly reordered the unions, then assigned the first 1/2 of villages to hang signage on their door as a visual commitment to mask-wearing, and 1/2 of villages to not have signage on their door.
4. Within the previous two randomizations, we randomly assigned 1/4 of villages to receive no incentive, 1/4 to receive a monetary award, and 1/2 to receive a certificate incentive. If there was an odd-number of villages within this randomization, then we broke the difference by rounding the number of villages in the randomization to the nearest whole number.
5. In villages without signage, we randomly ordered the villages and assigned the first 2/3 to receive texts encouraging mask-wearing, and the remaining 1/3 receive no such messages. If the number of villages was not divisible by thirds, then we broke the difference by rounding the number of villages to the nearest whole number.

Unions were assigned to household-level cross-randomizations using the following procedure. Note that each village was assigned to one and only one household-level randomization.

1. In villages with the signage randomization, we assigned 2/3 of villages to receive messages emphasizing the self-protection benefits of masks, and the remaining 1/3 to receive altruistic messages about the benefits of mask-wearing in addition to the self-protection messages. If the number of villages was not divisible by thirds, we broke the difference by rounding to the nearest whole number.

2. In villages without the signage randomization, we assigned 2/3 of villages to receive messages emphasizing the self-protection benefits of masks, and the remaining 1/3 to receive messages emphasizing the altruistic reasons to wear masks in addition to the self-protection messages.

3. In the villages without the signage randomization and no household-level altruism randomization, we asked some households to make a verbal commitment to be a mask-wearing household while the remaining were not asked to make a commitment.

4. In villages with the signage randomization and no household-level altruism randomization (and by definition, no village-level text message randomization), we assigned 1/4 of villages to receive no household-level text-message randomization, 1/2 of villages to have 50% of their households receive text-message reminders, and the remaining 1/4 of villages to have 100% of their households receive texts.

E Scoping and Recruitment

All households in selected villages were eligible for participation in the study. At each household, field staff sought consent to participate in respiratory symptom surveys from the adult who answered the door. The scoping staff that mapped enrolled villages were blind to study arm assignment. However, the implementation staff that consented households was not blind to study arm assignment. 93.3% of households consented to participate in the study and completed a baseline symptom survey. Of the households that were surveyed in the baseline household visit, 83.2% of households provided a response to the week 5 symptom survey. 94.4% of households provided a response to the week 9 symptom survey. 98.1% of households provided a response to the week 5 or week 9 symptom survey. There were no statistically significant differences between response rates in the treatment and control groups.

Individuals who reported symptoms any time during the 8-week study period were sought out for collection of a blood sample; blood sample collection was conducted only after additional

informed, written consent was provided. 39.7% of symptomatic participants agreed to blood collection. Blood consent rates are not significantly different in the treatment and control group and are comparable across all demographic groups, we cannot rule out that the composition of consenters differed between the treatment and control groups. If we assume that consenters and non-consenters have similar seroprevalence rates, then we would expect true symptomatic seroprevalence to be perhaps 2.5 times than the rates we report.

Surveillance staff were instructed to record details about the mask-wearing behavior of every person they saw while stationed at public places throughout the community: in mosques, at (predominantly open-air) markets, at outdoor tea stalls, on the main road, and outside restaurants. In other words, they conducted a census of all individuals within their field of view during surveillance activities. Surveillance staff were provided an example schedule for surveillance that suggested visiting 9 locations over the course of the day, spending one hour at each location. This included 2 hour outside of a restaurant or at a tea stall, 2 hours on the main road near the entrance to the village and transportation stations, 3 hours at a mosque, and 1 hour at a market. However, staff were free to vary the timing and location of their surveillance activities to maximum surveillance in crowded locations or locations with relatively higher numbers of people.

This observed sample is representative of the rural Bangladeshi population that is present in crowded public places during the day; this population is largely men, who have more social contacts outside the home than women. This is reflected in our surveillance in at mosques, markets, tea stall, restaurants, and on the main road, in which men constituted 88.2% of all observed adults in these areas. (Men constituted 100% of all observed adults at mosques and 87-89% of all observed adults in each of the other locations.)

There was no difference in the number of people observed in public areas between treatment and control groups. The distinct appearance of project-associated masks and elevated mask-wearing in treatment villages made it impossible to blind surveillance staff to study arm assignment. However, study staff were not informed about the exact purpose of the study.

F Details on Mask Materials and Design

In focus groups conducted prior to the study, participants said they preferred cloth over surgical masks because they perceived surgical masks to be single-use only and cloth masks to be more durable. Focus group participants also provided feedback on different cloth masks designs and sizes. Both types of masks were manufactured in Bangladesh. The cloth masks were produced by Bangladeshi garment factories within 6 weeks after ordering.

The cloth mask had an exterior layer of 100% non-woven polypropylene (70 grams/square meter [gsm]), two interior layers of 60% cotton / 40% polyester interlocking knit (190 gsm), an elastic loop that goes around the head above and below the ears, and a nose bridge. The surgical mask had three layers of 100% non-woven polypropylene, elastic ear loops, and a nose bridge. The filtration efficiency was 37% (standard deviation [SD] = 6%) for the cloth masks, and 95% (SD = 1%) for the surgical masks. The filtration efficiency test was conducted using a Fluke 985 particle counter that has a volumetric sampling rate of 2.83 liters per minute. The measurement was taken of particles 0.3–0.5 µm in diameter flowing through the material with a face velocity of 8.5 cm/s. In our internal testing, we found that cloth masks with an external layer made of Pellon 931 polyester fusible interface ironed onto interlocking knit with a middle layer of interlocking knit could achieve a 60% filtration efficiency. Upon discussions with the manufacturers, we learned that those materials could not be procured. Using materials that were available, the highest filtration efficiency possible was 37%.

G Details on Surveillance

The mask distribution and promotion was conducted by the Bangladeshi NGO GreenVoice, a grassroots organization with a network of volunteers across the country. Household surveys and surveillance were performed independently by Innovations for Poverty Action (IPA). The same staff member conducted surveillance at paired intervention and control villages at baseline and then once per week on weeks 1, 2, 4, 6, 8, and 10 after the intervention. The 10-week observation

was conducted two weeks after all intervention activities had ceased. We also collected longer-term data on mask-wearing behavior 20-27 weeks after the launch of interventions. Each village was observed on two alternating days of the week. Across all villages, observations took place on all seven days of the week, with observation in 150 villages occurring on Friday to over-sample days when mosques were most crowded. Observations generally took place from 9 am to 7 pm. In 10 unions we conducted audits to assess the validity of surveillance data by pairing one monitoring officer with surveillance staff; in all cases the difference in their results was <10%, our pre-determined threshold.

Surveillance staff observed a single individual and recorded that person as practicing physical distancing if s/he was at least one arm's length away from all other people. This is consistent with the WHO guideline that defines physical distancing as one meter of separation https://www.who.int/westernpacific/emergencies/covid-19/information/physical-distancing. Accessed January, 30 2021. Note that compliance with WHO guidelines does not require physical distancing; for example, members of the same household need not remain physically distant (and presumably would not change their distancing behavior as a result of our intervention).

After 5 weeks of surveillance in wave 1, it was clarified that surveillance staff should only record mask-wearing behavior of people who appear to be 18 years or older. Prior to this, some surveyors included children (especially older children) in their counts. Since the same staff member conducted surveillance in paired intervention and control villages, this change affected the treatment and control groups equally.

H Antibody Testing

Serum samples were diluted 1:100 with sample dilution buffer. 50 microliters of diluted specimens were added to the SCoV-2 antigen-coated microtiter strip plates. After one hour of incubation at 37°C, the plate was washed six times with wash buffer, and conjugate solution was added to each well. The plate was incubated for another 30 minutes at 37°C and washed six times with wash

buffer. 75 microliters of liquid TMB substrate were added to all wells followed by 20 minutes of incubation in the dark at room temperature before the reaction was stopped. The absorbance was read on a microplate reader at 450nm (GloMax® Microplate Reader, Promega Corporation, Madison, WI). After calibration according to positive, negative, and cut-off controls, the immunological status ratio (ISR) was calculated as the ratio of optical density divided by the cut-off value. Samples were considered positive if the ISR value was determined to be at least 1.1. Samples with an ISR value 0.9 or below were considered negative. Samples with equivocal ISR values were retested in duplicate, and resulting ISR values were averaged. Individuals were coded as symptomatic seropositive if they reported symptoms consistent with the WHO COVID-19 case definition, their blood was collected, and the antibody test was positive.

I Impact of Masks on Symptoms, Seroprevalence, and Seroconversions

Our primary outcome measures symptomatic seroprevalence: this is the fraction of individuals who are symptomatic during our intervention period and seropositive at endline. Some of these individuals may have antibodies from infections occurring prior to our intervention. If so, the impact of our intervention on symptomatic seroprevalence may understate the impact on symptomatic seroconversions occurring during our intervention (i.e. the fraction of symptomatic infections prevented by masks). In this section, we discuss the relationship between these two quantities.

Let SC, the symptomatic seroconversion rate, denote the probability that an individual is SARS-CoV-2 antibody-positive during our intervention and symptomatic. Then the symptomatic seroprevalence is $SS = SC + P_{prior}$, where P_{prior} denotes the probability that an individual was infected prior to our intervention *and* is symptomatic during our intervention for some non-COVID reason.

The change in seroconversions between the treatment and control group is given by $\Delta SC = SC(1) - SC(0)$ where the notation $SC(T_i)$ denotes the potential outcome of seroconversions as a function of treatment status. Our goal is to estimate $\Delta SC/SC(0)$, the percentage change in sero-

conversions as a result of our intervention.

We observe $\Delta SS = \Delta SC + \Delta P_{prior}$. Additionally, we observe $SS(0) = SC(0) + P_{prior}(0)$. Suppose that masks prevent a fraction α of non-COVID symptoms. Then, $P_{prior}(1) = (1-\alpha)P_{prior}(0)$ and $\Delta P_{prior} = -\alpha P_{prior}(0)$. Then we have:

$$\frac{\Delta SS}{SS(0)} = \frac{\Delta SC - \alpha P_{prior}(0)}{SC(0) + P_{prior}(0)} \tag{1}$$

Rearranging (and substituting $SC(0) = SS(0) - P_{prior}(0)$), we obtain:

$$\frac{\Delta SC}{SC(0)} = \frac{\Delta SS}{SS(0)} + \frac{P_{prior}(0)(\alpha + \frac{\Delta SS}{SS(0)})}{SS(0) - P_{prior}(0)} \tag{2}$$

Note that if we assume that symptomatic seroconversions fall by exactly the same fraction as other symptomatic conditions, then we also have $SC(1) = (1-\alpha)SC(0)$, and solving equation 2 gives $\frac{\Delta SS}{SS(0)} = -\alpha = \frac{\Delta SC}{SC(0)}$. In other words, the percentage change in seroconversions equals the percentage change in seroprevalence provided either that $P_{prior} = 0$ *or* if the intervention works only by alleviating symptoms (and does so equally for COVID-19 and non-COVID diseases).

More generally, if the intervention both alleviates symptoms and reduces infections, then the relative impact on symptomatic seroconversions and symptomatic seroprevalence will depend on whether masks are more effective at preventing COVID-19 or other respiratory diseases (with a larger proportional reduction in symptomatic seroconversions in the former case). The magnitude of the difference between symptomatic seroconversions and symptomatic seropositives will depend on the fraction of symptomatic seropositives which are pre-existing at baseline.

J Behavioral Mechanisms

Our intervention combines multiple distinct elements: we provide people with free masks; we provide information about why mask-wearing is important; we conduct mask promotion in the form of monitors encouraging people to wear masks and stopping non-mask-wearing individuals

on roads and public places to remind them about the importance of masks; we partner with local public officials to encourage mask-wearing at mosques and markets; and in some villages, we provide a variety of reminders and commitment devices as well as incentives for village leaders. In this section, we attempt to decompose which elements were most critical to increase mask use. We first report results from several cross-randomizations, and then we report non-randomized evidence based on changes over time as our intervention details changed between the rounds of piloting, launch of the full project, and thereafter.

J.1 Village-level Cross-randomizations

Results from the same regression specification as our primary analysis, adding indicators for each village-level cross-randomization are reported in Figure S3 and Table S16. *None* of the village-level cross-randomizations had any statistically significant impact on mask-wearing behavior, beyond our basic intervention package. These null effects are fairly precise (with standard errors ranging from 2.5-3.9 percentage points). Text message reminders, incentives for village-leaders, or explicit commitment signals explain little of the mask increase we document.

Figure S3: Village-Level Cross Randomizations

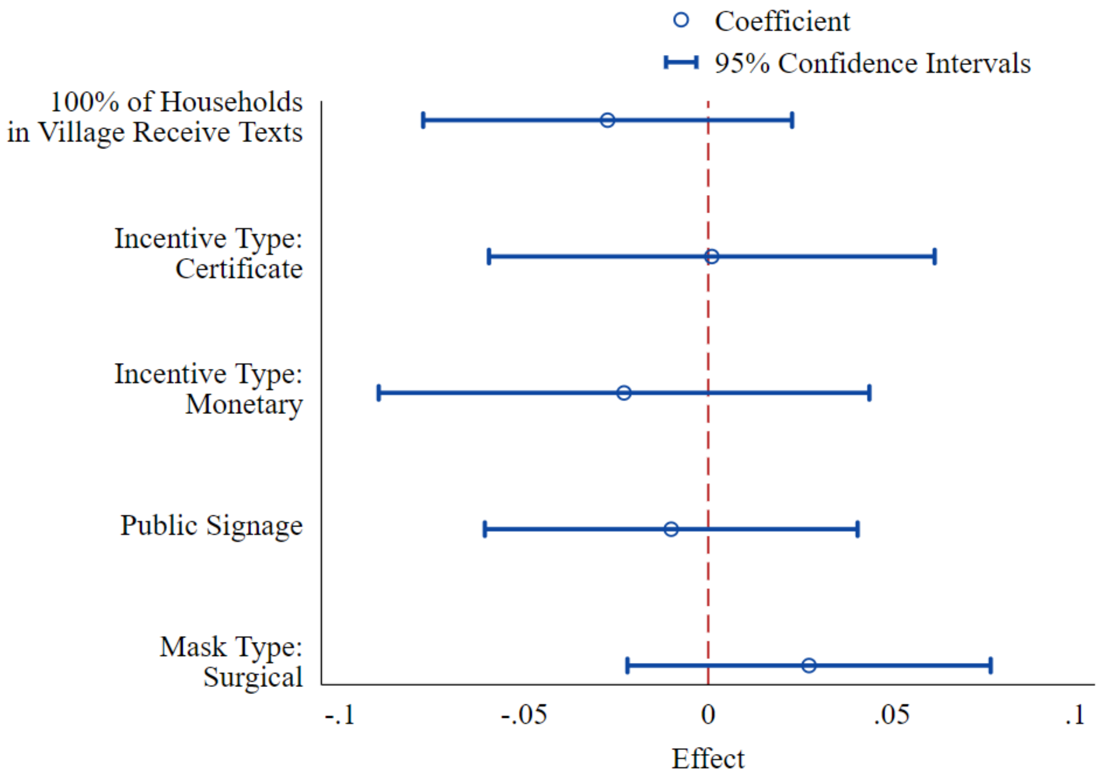

The figures corresponds to the regressions in S16, upper panel, among the full surveillance data.
Villages were assigned to the treatment or control arms of one of the following four village-level randomizations:
Texts: 0% or 100% of households in a village receive text reminders on the importance of mask-wearing;
Incentives: Villages either received no incentive, a certificate, or a monetary reward for meeting a mask-wearing threshold,
Public Signage: All or none of the households in a village are asked to publicly declare they are a mask-wearing households;
Mask Type: Villages receive either a cloth or surgical mask.
For a more detailed description of the village-level cross randomizations, see Section .

J.2 Household-level Cross-randomizations

We analyzed the effects of household-specific randomized treatments (e.g., verbal commitments or not) by regressing the probability of wearing a mask color corresponding to the treatment on indicators for each household-level randomization, as well as controls for color and surgical masks (recall that the mask-color corresponding to treatment varied across villages).

Results of the household-level cross-randomizations are reported in Figure S4 and Table S17. The coefficients indicate the impact of each cross-randomization relative to the core intervention (identified since some villages had no household randomization other than mask color). Once again, we saw no significant effects of any of the household-level cross-randomizations: compared to self-protection messaging alone, altruistic messaging had no greater impact on mask-wearing, and twice-weekly text messages and a verbal commitment had no significant effects.

We did see an impact of mask color on mask adoption. In villages where surgical masks were distributed, blue surgical masks were 2.7 percentage points more likely than green surgical masks to be observed. In villages where cloth masks were distributed, purple masks were 2.2 percentage points less likely than red masks to be observed.

Table S16: Village-Level Cross Randomizations

Coefficient	Full	No Active Promotion	Mosques	Markets	Other Locations
		No Baseline Controls			
Mask Type (Surgical)	0.027	0.027	0.062*	0.017	0.018
	(0.025)	(0.025)	(0.035)	(0.026)	(0.025)
Commitment w/ Signage	-0.010	-0.007	-0.019	-0.008	-0.009
	(0.026)	(0.026)	(0.034)	(0.027)	(0.026)
Incentive Type					
Monetary	-0.023	-0.026	0.013	-0.034	-0.026
	(0.034)	(0.034)	(0.045)	(0.034)	(0.035)
Certificate	0.001	-0.002	0.021	0.003	-0.009
	(0.031)	(0.031)	(0.038)	(0.031)	(0.031)
100% Text	-0.027	-0.023	-0.041	-0.024	-0.016
	(0.026)	(0.025)	(0.033)	(0.026)	(0.026)
		With Baseline Controls			
Mask Type (Surgical)	0.029	0.029	0.063*	0.018	0.022
	(0.025)	(0.025)	(0.034)	(0.026)	(0.025)
Commitment w/ Signage	-0.007	-0.003	-0.021	-0.004	-0.005
	(0.026)	(0.025)	(0.033)	(0.026)	(0.026)
Incentive Type					
Monetary	-0.021	-0.024	0.013	-0.031	-0.026
	(0.033)	(0.033)	(0.043)	(0.033)	(0.035)
Certificate	0.006	0.003	0.026	0.008	-0.006
	(0.031)	(0.030)	(0.039)	(0.030)	(0.031)
100% Text	-0.026	-0.022	-0.038	-0.024	-0.017
	(0.025)	(0.025)	(0.033)	(0.026)	(0.026)
N villages	286	286	286	286	286

Standard errors are in parentheses.
*** Significant at the 1 percent level. ** Significant at the 5 percent level. * Significant at the 10 percent level.
All regressions include an indicator for each control-intervention pair. The regressions ”with baseline control” include controls for the number of people observed in the baseline visit.
Baseline symptom rate is defined as the rate of surveyed individuals in a village who report symptoms coinciding with the WHO definition of a probable COVID-19 case. We assume that (1) all reported symptoms were acute onset, (2) all people live or work in an area with high risk of transmission of virus and (3) all people have been a contact of a probable or confirmed case of COVID-19 or are linked to a COVID-19 cluster.
‘No Active Promotion” refers to any time that surveillance was conducted while promotion was not actively occurring (regardless of the week of the intervention). This excludes surveillance during the Friday Jumma Prayers in the mosque, when promoters were present and actively encouraged mask wearing.
”Other Locations” include tea stalls, at the entrance of the restaurant as patrons enter, and the main road to enter the village.

Figure S4: Household-Level Cross Randomizations

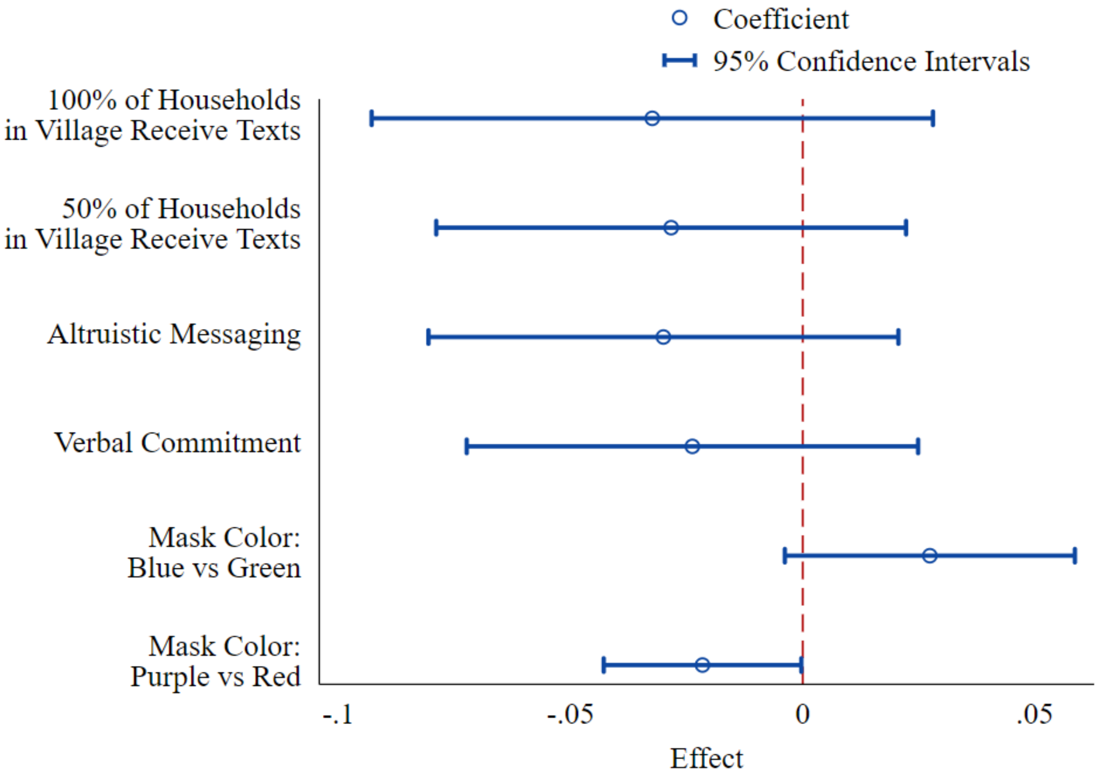

The figure corresponds to the regression presented in Table S17.
Villages were assigned to the treatment or control arms of one of the following four village-level randomizations:
Texts: 0%, 50% of 100% of households in a village receive text reminders on the importance of mask-wearing;
Messaging: Households receive messaging emphasizing the altruistic or self-protective benefits of mask-wearing;
Verbal Commitment: Households were asked to verbally commit to mask-wearing;
Mask Colors: Surgical masks distributed to households were blue or green. Cloth masks distributed to households were purple or red.
For a more detailed description of the household-level cross-randomizations, see Section .

Table S17: Household-Level Cross-Randomizations

Coefficient	Full
Household-Level Text Randomization	
50% of Households in Village	-0.032
	(0.031)
100% of Households in Village	-0.028
	(0.026)
Altruistic Messages	-0.030
	(0.026)
Verbal Commitment	-0.024
	(0.025)
Mask Color	
Blue vs Green	0.027*
	(0.016)
Purple vs Red	-0.022**
	(0.011)
N villages	286

Standard errors are in parentheses.
*** Significant at the 1 percent level. ** Significant at the 5 percent level. * Significant at the 10 percent level.
The regression includes a control for the mask type to separate the effect of mask colors.
Surgical masks distributed to households were blue or green. Cloth masks distributed to households were purple or red.

J.3 Mask Promotion

As noted above, we ran two pilots prior to launching the full project. Both pilots were conducted in Naogaon and Joypurhat districts, but in different unions. While the unions were not selected at random, there was no systematic difference in the selection process between the two pilots. In both cases, unions were selected based on convenience and proximity to existing Greenvoice personnel.

Both pilots included elements 1, 2, 3, and 5 enumerated in Section : masks were distributed at households, markets, and mosques, and there was role-modeling and advocacy by local leaders, including Imams. The second pilot added to these elements explicit mask promotion: mask promoters patrolled public areas a few times a week and asked those not wearing masks to put on a mask. The full intervention also included mask promotion.

The comparison between the two pilots is thus instructive about the impact of active mask promotion. This comparison is shown in Table S10. The difference is striking. The first pilot increased mask-use by 10.9 percentage points (insignificantly different from zero). The second pilot, which included mask promotion, increased mask-use by 28.4 percentage points, comparable to the 29.0 percentage points we see several months later in our full intervention. The presence of mask promotion appears to be crucial for the success of our intervention.

K Statistical Analysis

This section describes details of our statistical analyses.

Mask-Wearing We created a data set with an observation for each village j. We defined proper mask use as anyone wearing either a project mask or an alternative face-covering that covered their mouth and nose. We considered two definitions of the proportion of observed individuals wearing masks (p_j). In our primary specification, we defined p_j using all observed adults. In a secondary specification, we considered adults observed only in locations where we there was not simultaneous mask distribution. The purpose of this second specification was to investigate separately whether

the intervention increased mask-wearing in places where we did not have promoters on site.

Our goal was to estimate the impact of the intervention on the probability of mask-wearing, defined as $\psi_1 = E_x[E(p_j|T_j = 1, x_j) - E(p_j|T_j = 0, x_j)]$ where T_j is an indicator for whether a village was treated and x_j is a vector of the village-level covariates, including the prevalence of baseline mask-wearing in each village (constructed analogously to p_j), baseline respiratory symptom rates, and indicators for each pair of villages from our pairwise stratification method.

We estimated this equation at the village-level with an ordinary least squares regression, using analytic weights proportional to the number of observed individuals (the denominator of p_j) and heteroskedastic-robust standard errors. In this specification, the dependent variable is p_j, the independent variable of interest was T_j, and controls were included for the x_j covariates.

Physical Distancing Using analogous methods, we estimated the impact of the intervention on the probability that wearing a mask influenced physical distancing (being within one arm's length of any other person at the time of observation).

K.1 Estimating Effects of Village-level Cross-randomizations

We analyze all four village level cross-randomizations jointly via a linear regression:

$$E(p_j|T_j, x_j, D_k) = \beta T_j + \sum_k D_k \delta_k + x_j \gamma \tag{3}$$

where $D_k = 1$ if the village has been assigned to the intervention group of the village-level cross-randomization denoted by letter k, and 0 otherwise. This specification is otherwise identical to our estimating equation for the impact of intervention on mask-wearing, with the addition of the D_k terms.

K.2 Estimating Effects of Household-level Cross-randomizations

To evaluate the effect of household-level cross-randomizations, we constructed a regression with an observation for each *village* where we ask whether masks of the color representing the treatment were more commonplace than masks of the color representing the control. In each village, we computed Δ_j, the difference in the fraction of individuals wearing treatment mask colors vs. control mask colors. We alternated across villages which color corresponds to intervention, so we can control directly for whether specific colors are more popular (denote these by d_{jc}; $d_{jc} = 1$ if treated masks in village j are color c). We index the various household randomizations by m. Our estimate for each household randomization will be α_{0m}, given by:

$$E(\Delta_j | d_{jc}) = \alpha_{0m} + \sum_c \alpha_c d_{jc} + surgical_j \tag{4}$$

α_{0m} tells us how much more likely individuals are to wear masks of the treated color than masks of the control color. $surgical_j$ is, as its name implies, a dummy for whether surgical masks were distributed in village j. We estimate this equation at the village-level by ordinary least squares, using analytic weights proportional to the number of observed individuals (the denominator of Δ_j) and heteroskedasticity-robust standard errors.

L Additional Balance Tests

While our stratification procedure should have achieved balance with respect to variables observed at the time of randomization, given the many possible opportunities for errors in implementation, we nonetheless confirm in this Appendix L that our control and treatment villages resemble each other at baseline with respect to key variables of interest. This assessment was not preregistered. We find that the control and treatment groups are balanced with respect to our primary outcomes of interest: mask-wearing, symptoms and symptomatic seropositivity. In this Appendix we investigate several other covariates and find a few small imbalances, and conduct robustness checks.

For example, we find more 18-30 year olds in the treatment group, perhaps because households reported teenagers as 18 in order to receive more masks; our results are robust to dropping this age range.

In Table S4 we present balance test results for our mask-wearing specification (at the village level). In our main specification, this is a regression of mask-wearing on a constant, an intervention indicator, and indicators for each control-intervention pair with analytic weights proportional to the number of adults recorded in the baseline household survey as well as heteroskedasticity robust standard errors. For the balance tests, we replace the dependent variable with several variables measured at baseline: symptomatic seroprevalence, WHO-Defined COVID-19 symptoms, and baseline mask-wearing rate. We find that all of these variables appear balanced.

In Table S18, we report results from analogous balance tests based on the specification used for our primary biological outcome. We replace the dependent variable (symptomatic seroprevalence) with baseline covariates of interest to assess balance. We also report a bottom-line F-test which again fails to reject balance.

Table S18: Balance Tests (Individual-Level)

	Baseline Symptomatic Seroprevalence	Baseline WHO-Defined COVID-19 Symptoms	Baseline Mask-Wearing Rate
Summary Statistics			
Intervention Rate	0.00020	0.02468	0.11829
Control Rate	0.00022	0.02342	0.11990
Balance Tests			
Intervention Coefficient	-0.00001<br>(0.00004)	0.00081<br>(0.00113)	0.00093<br>(0.00391)
N individuals	304,726	304,726	304,726
N villages	572	572	572
F		0.76	
Joint-Test Prob > F		0.8596	

Standard errors are in parentheses.
*** Significant at the 1 percent level. ** Significant at the 5 percent level. * Significant at the 10 percent level.
The baseline rate of mask-wearing was measured through observation over a 1-week period, defined as the rate of those observed who wear a mask or face covering that covers the nose and mouth.
The analysis includes all people surveyed in the baseline household visits, excluding individuals that we did not collect midline or endline symptoms for, symptomatic individuals that we did not collect blood from, and individuals that we drew blood from but did not test their blood.

We also ran balance tests with respect to several other covariates and detected a few balance failures. While small in magnitude, we investigate these further in order to understand the severity of the underlying problem.

Table S19 highlights these balance failures. Specifically, we find imbalances with respect to household count, age and household size. On average, treatment villages have 16 more households, the treatment villages have 0.4 percentage points more people younger than 30, and treatment households have 0.02 more members. While small in magnitude, these imbalances are unlikely to have arisen by chance given the size of our sample.

We believe the imbalances with respect to age and household size likely arose because households in the treatment group were more likely to report teenagers as being over 18 in order to receive additional masks. We believe the imbalance with respect to the number of households likely occurred for a similar reason, with implementers in the treatment group including more "borderline" households as part of the village in order to distribute masks to those households.

To check for these mechanisms, we drop from the sample individuals under 30 and villages with over 350 households – the latter only very coarsely targets "extra" households that lie on the border of villages. After imposing these restrictions, we find in Table S20 that the imbalances with respect to age and household size disappear entirely (this also occurs with the age restriction alone), and the imbalance with respect to household count shrinks by 25% but remains significant. In Table S21, we repeat our primary specification in this restricted sample with better balance and find that our results are qualitatively unchanged.

Table S19: Additional Balance Tests (Individual-Level)

	Household Count	Proportion Female	Proportion Below 30	Average Household Size
	Summary Statistics			
Intervention Group	230	0.5127	0.3348	2.6506
Control Group	213	0.5114	0.3288	2.6158
	Balance Tests			
Intervention Coefficient	16***	0.0017	0.0044**	0.0327***
	(3)	(0.0011)	(0.0018)	(0.0094)
N individuals	304,726	304,726	304,726	304,726
N villages	572	572	572	572
F		55.62		
Joint-Test Prob $>$ *F*		0.0000		

Standard errors are in parentheses.
*** Significant at the 1 percent level. ** Significant at the 5 percent level. * Significant at the 10 percent level.

Table S20: Additional Balance Tests (Individual-Level, After Sample Selection)

	Household Count	Proportion Female	Proportion Below 40	Average Household Size
Removing All People Below 30 & All Villages With More than 350 Households				
Intervention Coefficient	12***	0.0034**	-0.0008	0.0086
	(2)	(0.0013)	(0.0020)	(0.0057)
N individuals	197,615	197,615	197,615	197,615
N villages	563	563	563	563
F		33.63		
Joint-Test Prob > F		0.0000		

Standard errors are in parentheses.
*** Significant at the 1 percent level. ** Significant at the 5 percent level. * Significant at the 10 percent level.
The sample excludes an additional 107,111 individuals up to the age of 30, and 9 villages that have more than 350 households.

Table S21: Symptomatic Seroprevalence (With Controls and Additional Sample Selection)

	Intervention Effect	Intervention Effect by Mask Type
Controlling for Number of Households and Sex		
Intervention Coefficient	-0.0006* (0.0003)	
Intervention Coefficient for Surgical Mask Villages		-0.0008* (0.0004)
Intervention Coefficient for Cloth Mask Villages		-0.0002 (0.0005)
Average Symptomatic Seroprevalence Rate in Paired Control Villages§	0.0076	0.0076
N individuals	304,726	304,726
N villages	572	572
After Additional Sample Selection		
Intervention Coefficient	-0.0008* (0.0004)	
Intervention Coefficient for Surgical Mask Villages		-0.0011* (0.0006)
Intervention Coefficient for Cloth Mask Villages		-0.0001 (0.0007)
Average Symptomatic-Seroprevalence Rate in Paired Control Villages§	0.0091	0.0091
N individuals	197,615	197,615
N villages	563	563

Standard errors are in parentheses.
*** Significant at the 1 percent level. ** Significant at the 5 percent level. * Significant at the 10 percent level.
All regressions include an indicator for each control-intervention pair.
The regression in the top panel includes controls for baseline rates of mask wearing, baseline symptom rates, number of households in a village, and sex.
The regression in the bottom panel controls for baseline rates of mask wearing and baseline symptom rates.
§We report the mean rate of symptomatic-seroprevalence at endline. This is not equivalent to the coefficient on the constant due to the inclusion of the pair indicators as controls.
The analysis includes all people surveyed in the baseline household visits, excluding individuals that we did not collect midline or endline symptoms for, symptomatic individuals that we did not collect blood from, and individuals that we drew blood from but did not test their blood.
The bottom panel runs sample excludes an additional 107,111 individuals up to the age of 30 and 9 villages that have more than 350 households.

M Persistence of Mask-Wearing Behavior

In Table S22, we report estimates of our primary specification separately by week of surveillance. Week 10 is especially interesting, as it was two weeks after intervention activities ceased. This analysis was not preregistered.

We find no evidence that the impact of the intervention attenuates over the 10 weeks. In the 414 villages for which we have 10 weeks of surveillance, the point estimates are slightly smaller in week 10 (a 23.3 percentage point increase) than week 1 (30.4 percentage points), although this difference is not statistically significant. This is consistent with social norms around mask-wearing taking hold, where adoption by some in the community has a demonstration effect that encourages subsequent adoption by others. If mask-wearing was driven by a "novelty factor" associated with our mask promotion campaign, we would have instead expected some attenuation over the course of the 8 weeks of intervention. The point estimates of the impact of intervention by week for the panel of 414 villages for which we have data in all weeks are plotted in Figure S5.

We additionally conducted a follow-up surveillance 5 months after the start of the intervention (20-27 weeks, depending on the wave). Mask-wearing had declined to 14.1% in the control group and 22.4% in the intervention group (a regression adjusted difference of 0.10 [0.08,0.13]).

Table S22: Persistence of Mask-Wearing

	Week from Baseline Observation						
	1	2	4	6	8	10	Followup
				Consistent Panel			
Intervention Coefficient	0.304*** (0.016)	0.284*** (0.016)	0.290*** (0.016)	0.286*** (0.016)	0.261*** (0.016)	0.233*** (0.017)	0.102*** (0.011)
N villages	414	414	414	414	414	414	414
				All Villages			
Intervention Coefficient	0.300*** (0.014)	0.285*** (0.014)	0.291*** (0.014)	0.298*** (0.015)	0.261*** (0.014)	0.230*** (0.015)	0.094*** (0.010)
N villages	542	558	548	550	528	508	546

Standard errors are in parentheses.
*** Significant at the 1 percent level. ** Significant at the 5 percent level. * Significant at the 10 percent level.
All regressions include an indicator for each control-intervention pair, baseline rates of mask-wearing and baseline symptom rates.
Baseline symptom rate is defined as the rate of surveyed individuals in a village who report symptoms coinciding with the WHO definition of a probable COVID-19 case. We assume that (1) all reported symptoms were acute onset, (2) all people live or work in an area with high risk of transmission of virus and (3) all people have been a contact of a probable or confirmed case of COVID-19 or are linked to a COVID-19 cluster.
"Followup" surveillance occurred between June 4th and June 8th 2021, which is anywhere from 20 to 27 weeks after baseline for each village.
This analysis estimates separate intervention effects 1, 2, 4, 6, 8, 10 weeks, and 20-27 weeks after baseline observation. The top panel runs the regressions only among a consistent panel of 414 villages that have all 10 weeks and the subsequent followup observation. The results of the analysis are displayed graphically in Figure S5.
The bottom panel is run among all villages which have surveillance data for that period of observation, as well as the baseline period.
The 10th week of observation and the followup observation occur after all active promotion of mask-wearing has ceased.

Figure S5: Persistence of Mask-Wearing

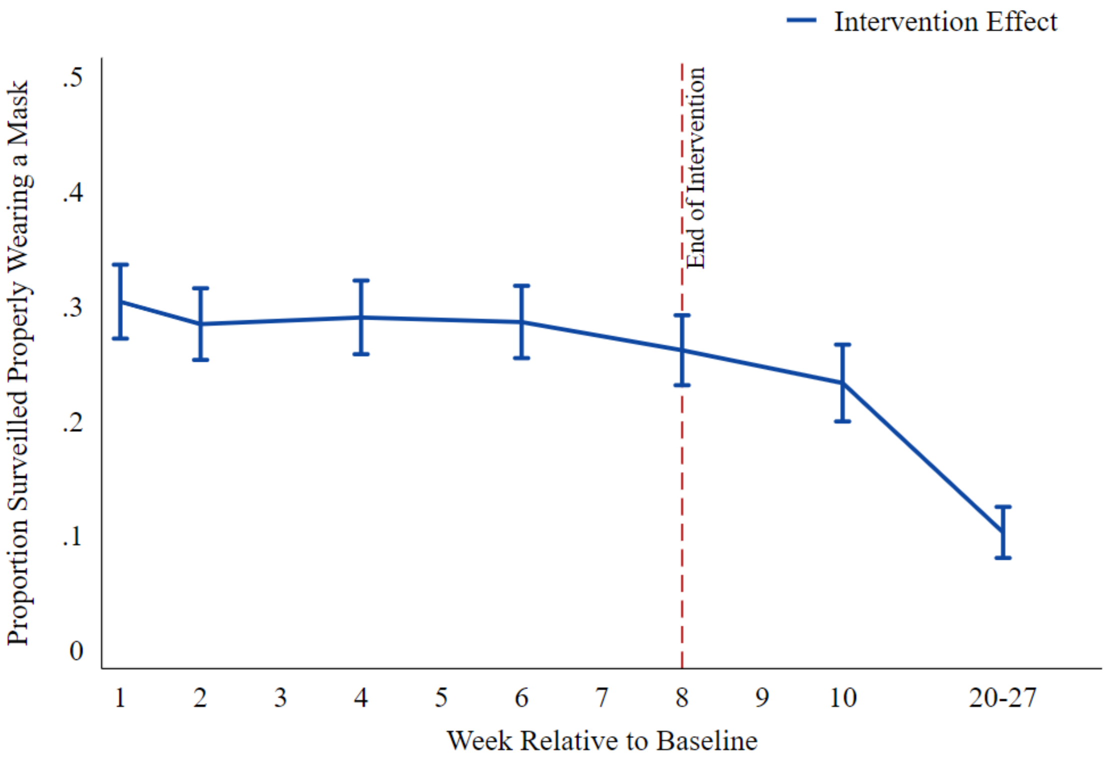

The figure corresponds to the regressions presented in Table S22, top panel. We present the effect of the intervention separately across weeks 1, 2, 4, 6, 8, 10, and 20-27 weeks after the baseline observation with 95% confidence intervals. The 20-27 week observation was collected during our "Followup" surveillance between June 4th and June 8th 2021, which is anywhere from 20 to 27 weeks after baseline for each village.

The analysis is run across a panel of 414 villages with observation through the entirety of the study. The 10th week of observation and the followup observation occur after all active promotion of mask-wearing has ceased.

N Imputed Symptomatic Seroprevalence

In this section, we analyze the results of our intervention by wave, as well as assessing the sensitivity of our analysis to alternative methods of imputing missing values. These analyses turn out to be related, as there is one wave with imbalanced consent rates, meaning that dropping non-consenters distorts the treatment and control comparison.

In Table S23, we analyze the impact of our intervention on mask-wearing and physical distancing by waves. In all waves, mask-wearing increased by between 24.2 and 35.7 percentage points, and physical distancing increased by between 4.2 and 7.7 percentage points.

When we analyze our second stage results by wave, reported in the top panel of Table S24, we find that most waves have comparable effect sizes with two exceptions: in wave 7 we find an especially large impact of masks on symptomatic seropositivity and in wave 2 we find an opposite signed impact on symptomatic seropositivity.

Probing further, we believe the opposite signed result in wave 2 is due to imbalanced consent rates in that wave. In Table S25, we show consent rates for control and treatment groups by waves. Since we drop individuals who do not consent in our primary specification, lower consent rates appear as lower rates of symptomatic seropositivity. In Table S1, we found that consent rates were balanced across treatment and control groups, as well as by age and gender. In Table S25, we find that consent rates are generally comparable across waves, although they appear notably lower in the control group of wave 2 relative to the treatment group. Consent rates are somewhat lower in the control group of wave 5 and the treatment group of wave 7, although differences are not as stark.

To check whether our results are driven by differential consent, we consider an alternative method of dealing with missing data. Instead of dropping symptomatic individuals who did not consent to blood collection, we impute for those individuals the mean (conditional) seropositivity observed among all individuals in the data.

These results are shown in the bottom panel of Table S24. Several points are worth noting. First, the point estimate for the main effect of masks on seropositivity becomes substantially larger.

There is a mechanical effect due to the fact that rates of symptomatic seropositivity in the data now increase by a factor of 2.5 since we previously dropped the 60% of symptomatic individuals who did not consent to blood samples. Scaling our original estimate by this factor would give an effect size of -0.0018. The effect size with seropositivity imputed is slightly larger, at -0.0022, and much more precisely estimated than the main effect in our data. Additionally, with this imputation method, the anomalous Wave 2 result disappears.

Table S26 shows our main results broken out by age, with the top panel using our pre-registered sample and the bottom panel showing the same results with the imputation method. The results are quite similar in both cases, with larger effects at older ages.

In Table 4 in the main text, we further disaggregate the results by cloth and surgical masks. Two points are notable in these results. First, when we drop non-consenters (in the original specification), cloth masks appear to impact symptomatic seropositivity only in the 40-50 age group (and have an insignificant effect pooling all ages). However, if we instead impute seropositivity at the average value for non-consenters, cloth masks appear most effective at older ages, and about as effective as surgical masks.

Tables S28 and S6 report analogous results with respect to gender for symptoms and symptomatic seropositivity. We see a similar pattern to the age results: we see similar effects for both genders for symptoms and symptomatic seroposivity when we impute seropositivity at the average value for non-consenters. If we instead drop non-consenters, the symptomatic seropositivity estimates for men become less precise and are no longer significantly different from zero.

Table S23: Mask-Wearing and Physical Distancing by Wave, Controlling for Baseline Variables

	Full	Wave 1	Wave 2	Wave 3	Wave 4	Wave 5	Wave 6	Wave 7
				Proper Mask-Wearing				
Intervention Coefficient	0.288***	0.282***	0.350***	0.258***	0.242***	0.310***	0.257***	0.357***
	(0.012)	(0.053)	(0.044)	(0.021)	(0.028)	(0.025)	(0.025)	(0.033)
				Physical Distancing				
Intervention Coefficient	0.051***	0.077***	0.056***	0.060***	0.045***	0.042***	0.048***	0.068***
	(0.005)	(0.024)	(0.015)	(0.017)	(0.010)	(0.014)	(0.013)	(0.016)
N villages	572	28	52	78	102	118	110	84

Standard errors are in parentheses.
*** Significant at the 1 percent level. ** Significant at the 5 percent level. * Significant at the 10 percent level.
All regressions include an indicator for each control-intervention pair. The regressions include controls for baseline rates of physical distancing and baseline symptom rates.
Baseline symptom rate is defined as the rate of surveyed individuals in a village who report symptoms coinciding with the WHO definition of a probable COVID-19 case. We assume that (1) all reported symptoms were acute onset, (2) all people live or work in an area with high risk of transmission of virus and (3) all people have been a contact of a probable or confirmed case of COVID-19 or are linked to a COVID-19 cluster.

Table S24: Symptomatic-Seroprevalence by Wave, With Baseline Controls

	All	Wave 1	Wave 2	Wave 3	Wave 4	Wave 5	Wave 6	Wave 7
Pre-Registered Sample: Drop Individuals Without Blood Draws								
Intervention Coefficient	-0.0007**	-0.0008	0.0031***	-0.0011	-0.0009	-0.0004	-0.0006	-0.0031***
	(0.0003)	(0.0011)	(0.0009)	(0.0009)	(0.0008)	(0.0006)	(0.0008)	(0.0008)
Avg. Symptomatic-Seroprevalence in Paired Control Villages§	0.0076	0.0061	0.0036	0.0067	0.0091	0.0067	0.0085	0.0095
N Individuals	304,726	14,606	27,132	43,602	56,607	62,419	57,397	42,963
N Villages	572	572	572	572	572	572	572	572
Imputing Symptomatic-Seroprevalence for Missing Blood Draws								
Intervention Coefficient	-0.0022***	0.0001	0.0007	-0.0010	-0.0027	-0.0013	-0.0028**	-0.0054***
	(0.0005)	(0.0014)	(0.0010)	(0.0014)	(0.0017)	(0.0012)	(0.0011)	(0.0011)
Avg. Symptomatic-Seroprevalence in Paired Control Villages§	0.0189	0.0104	0.0113	0.0143	0.0221	0.0192	0.0221	0.0217
N Individuals	321,948	14,995	28,168	45,358	60,237	66,303	61,185	45,702
N Villages	572	572	572	572	572	572	572	572

Standard errors are in parentheses.
*** Significant at the 1 percent level. ** Significant at the 5 percent level. * Significant at the 10 percent level.
All regressions include an indicator for each control-intervention pair. The regressions include controls for baseline rates of proper mask wearing and baseline symptom rates.
Baseline Symptom Rate is defined as the rate of surveyed individuals in a village who report symptoms coinciding with the WHO definition of a probable COVID-19 case. We assume that (1) all reported symptoms were acute onset, (2) all people live or work in an area with high risk of transmission of virus and (3) all people have been a contact of a probable or confirmed case of COVID-19 or are linked to a COVID-19 cluster.
§We report the mean rate of symptomatic seroprevalence at endline. This is not equivalent to the coefficient on the constant due to the inclusion of the pair indicators as controls.
The analysis in the top panel utilizes the pre-registered sample, equivalent to Table S7; it includes all people surveyed in the baseline household visits, excluding individuals that we did not collect midline or endline symptoms for, symptomatic individuals that we did not collect blood from, and individuals that we drew blood from but did not test their blood.
The analysis in the bottom panel replicates the regression in the top panel, but imputes the seropositivity of individuals for who we did not draw blood. For symptomatic individuals we did not draw blood from, we simulate their symptomatic-seroprevalence status by using the average rate of conditional seropositivity among all symptomatic individuals. This analysis includes all people surveyed in the baseline household visits, excluding individuals that we did not collect midline or endline symptoms for.

Table S25: Endline Blood Collection Consent Rates by Wave

	Number of Individuals		Rate of COVID Symptoms		Blood Draw Consent Rate	
	Treatment	Control	Treatment	Control	Treatment	Control
Wave 1	10,412	8,810	4.0%	4.1%	51.0%	49.7%
Wave 2	18,378	14,862	4.8%	5.2%	44.2%	33.9%
Wave 3	25,042	23,700	6.4%	6.3%	46.3%	44.7%
Wave 4	31,999	28,598	8.8%	10.4%	40.9%	42.0%
Wave 5	35,216	33,777	8.3%	8.4%	35.9%	31.8%
Wave 6	33,424	31,954	8.8%	10.1%	38.5%	37.8%
Wave 7	23,851	22,160	7.2%	9.9%	38.8%	44.0%

“Rate of COVID symptoms” reports the proportion of individuals that report WHO-defined COVID symptoms in the midline or endline surveys.
“Blood Draw Consent Rate” reports the proportion of individuals that consented to a blood draw in the endline, conditional on being symptomatic.

Table S26: Symptomatic Seroprevalence by Age Groups, With Baseline Controls, Expressed in Prevalence Ratios

	All	< 40 Y.O.	40-49 Y.O.	50-59 Y.O.	≥ 60 Y.O.
Pre-Registered Sample: Drop Individuals Without Blood Draws					
Intervention Prevalence Ratio	0.905** [0.815, 0.995]	0.995 [0.886, 1.104]	0.917 [0.763, 1.070]	0.791*** [0.634, 0.948]	0.779** [0.610, 0.947]
Avg. Symptomatic-Seroprevalence in Paired Control Villages§	0.0076	0.0055	0.0095	0.0108	0.0104
N Individuals	287,349	146,306	35,839	24,086	27,943
N Villages	538	480	384	348	360
Imputing Symptomatic-Seroprevalence for Missing Blood Draws					
Intervention Prevalence Ratio	0.879*** [0.820, 0.938]	0.899*** [0.831, 0.967]	0.929 [0.836, 1.022]	0.924 [0.820, 1.028]	0.731*** [0.627, 0.834]
Avg. Symptomatic-Seroprevalence in Paired Control Villages§	0.0189	0.0152	0.0226	0.0229	0.0251
N Individuals	321,383	177,708	51,676	37,340	43,431
N Villages	570	566	528	504	534

Standard errors are in parentheses.
*** Significant at the 1 percent level. ** Significant at the 5 percent level. * Significant at the 10 percent level.
All regressions include an indicator for each control-intervention pair. The regressions also include controls for baseline rates of proper mask wearing and baseline symptom rates.
Baseline Symptom Rate is defined as the rate of surveyed individuals in a village who report symptoms coinciding with the WHO definition of a probable COVID-19 case. We assume that (1) all reported symptoms were acute onset, (2) all people live or work in an area with high risk of transmission of virus and (3) all people have been a contact of a probable or confirmed case of COVID-19 or are linked to a COVID-19 cluster.
§We report the mean rate of symptomatic seroprevalence at endline. This is not equivalent to the coefficient on the constant due to the inclusion of the pair indicators as controls.
The analysis in the top panel utilizes the pre-registered sample, equivalent to Table 2; it includes all people surveyed in the baseline household visits, excluding individuals that we did not collect midline or endline symptoms for, symptomatic individuals that we did not collect blood from, and individuals that we drew blood from but did not test their blood.
The analysis in the bottom panel replicates the regression in the top panel, but imputes the seropositivity of individuals for who we did not draw blood. For symptomatic individuals we did not draw blood from, we simulate their symptomatic-seroprevalence status by using the average rate of conditional seropositivity among all symptomatic individuals. This analysis includes all people surveyed in the baseline household visits, excluding individuals that we did not collect midline or endline symptoms for.

Table S27: Symptomatic Seroprevalence by Sex with Baseline Controls, Expressed in Prevalence Ratios

	All	Male	Female
Pre-Registered Sample: Drop Individuals Without Blood Draws			
Intervention Prevalence Ratio	0.905**	0.947	0.869**
	[0.815, 0.995]	[0.837, 1.058]	[0.755, 0.984]
Intervention Prevalence Ratio for Surgical Villages	0.889**	0.905	0.870*
	[0.780, 0.997]	[0.779, 1.031]	[0.724, 1.016]
Intervention Prevalence Ratio for Cloth Villages	0.942	1.049	0.869
	[0.781, 1.103]	[0.829, 1.268]	[0.694, 1.044]
Average Symptomatic Seroprevalence in Paired Control Villages§	0.0076	0.0068	0.0083
N Individuals	287,349	129,308	133,898
N Villages	538	496	486
Imputing Symptomatic-Seroprevalence for Missing Blood Draws			
Intervention Coefficient	0.879***	0.881***	0.876***
	[0.820, 0.938]	[0.811, 0.952]	[0.801, 0.951]
Intervention Coefficient for Surgical Villages	0.873***	0.847***	0.894**
	[0.801, 0.945]	[0.766, 0.929]	[0.797, 0.990]
Intervention Coefficient for Cloth Villages	0.890**	0.953	0.842***
	[0.787, 0.993]	[0.819, 1.087]	[0.725, 0.959]
Average Symptomatic Seroprevalence in Paired Control Villages§	0.0189	0.0178	0.0200
N Individuals	321,383	156,302	164,004
N Villages	570	568	566

Confidence Intervals are in brackets.
*** Significant at the 1 percent level. ** Significant at the 5 percent level. * Significant at the 10 percent level.
All regressions include an indicator for each control-intervention pair. The regressions include controls for baseline rates of proper mask wearing and baseline symptom rates. Baseline Symptom Rate is defined as the rate of surveyed individuals in a village who report symptoms coinciding with the WHO definition of a probable COVID-19 case. We assume that (1) all reported symptoms were acute onset, (2) all people live or work in an area with high risk of transmission of virus and (3) all people have been a contact of a probable or confirmed case of COVID-19 or are linked to a COVID-19 cluster. §We report the mean rate of symptomatic seroprevalence at endline. This is not equivalent to the coefficient on the constant due to the inclusion of the pair indicators as controls. The analysis in the top panel utilizes the pre-registered sample, equivalent to Table S7; it includes all people surveyed in the baseline household visits, excluding individuals that we did not collect midline or endline symptoms for, symptomatic individuals that we did not collect blood from, and individuals that we drew blood from but did not test their blood. The analysis in the bottom panel replicates the regression in the top panel, but imputes the seropositivity of individuals for who we did not draw blood. For symptomatic individuals we did not draw blood from, we simulate their symptomatic-seroprevalence status by using the average rate of conditional seropositivity among all symptomatic individuals. This analysis includes all people surveyed in the baseline household visits, excluding individuals that we did not collect midline or endline symptoms for.

Table S28: WHO-Defined COVID Symptoms by Sex, Expressed in Prevalence Ratios

	All	Male	Female
	No Baseline Controls		
Intervention Coefficient	0.885***	0.884***	0.886***
	[0.834, 0.934]	[0.834, 0.933]	[0.823, 0.950]
Intervention Coefficient for Surgical Villages	0.874***	0.864***	0.882***
	[0.809, 0.939]	[0.803, 0.926]	[0.800, 0.964]
Intervention Coefficient for Cloth Villages	0.907**	0.922*	0.895**
	[0.823, 0.991]	[0.838, 1.005]	[0.798, 0.991]
Avg. Symptomatic Seroprevalence in Paired Control Villages§	0.0860	0.0824	0.0894
	With Baseline Controls		
Intervention Coefficient	0.884***	0.884***	0.885***
	[0.834, 0.934]	[0.837, 0.932]	[0.822, 0.947]
Intervention Coefficient for Surgical Villages	0.865***	0.857***	0.873***
	[0.803, 0.928]	[0.800, 0.915]	[0.792, 0.953]
Intervention Coefficient for Cloth Villages	0.922*	0.938	0.908*
	[0.838, 1.005]	[0.854, 1.022]	[0.812, 1.004]
N Individuals	321,948	156,846	165,102
N Villages	572	572	572

Confidence Intervals are in brackets.
*** Significant at the 1 percent level. ** Significant at the 5 percent level. * Significant at the 10 percent level.
All regressions include an indicator for each control-intervention pair. The regressions "with baseline controls" include controls for baseline rates of mask-wearing and baseline symptom rates.
Baseline Symptom Rate is defined as the rate of surveyed individuals in a village who report symptoms coinciding with the WHO definition of a probable COVID-19 case. We assume that (1) all reported symptoms were acute onset, (2) all people live or work in an area with high risk of transmission of virus and (3) all people have been a contact of a probable or confirmed case of COVID-19 or are linked to a COVID-19 cluster. §We report the mean rate of symptomatic seroprevalence at endline. This is not equivalent to the coefficient on the constant due to the inclusion of the pair indicators as controls.
The analysis includes all people surveyed in the baseline household visits, excluding individuals that we did not collect midline or endline symptoms for.

O Variation of Effects

In this Appendix, we investigate how WHO-Defined COVID symptoms and symptomatic seropositivity relate cross-sectionally to changes in mask-wearing and changes in physical distancing relative to baseline. This comparison should be interpreted with caution, since the observational variation across villages in mask-wearing and measured physical distancing is not random. For example, within the treatment or control group, some villages might have more mask-wearing precisely because people were observed with COVID-19 symptoms. Were this the case, even if masks reduced COVID-19, we might see a positive relationship between mask-wearing and biological outcomes; a similar bias could be present for physical distancing.

With these caveats in mind, Figure S6 shows the relationship between each biological outcome variable and the changes in mask-wearing and physical distancing graphically. Table S29 shows coefficients from a regression of each outcome on the respective change, controlling for the same covariates as our baseline regression, except for pair fixed effects (omitting these effects is necessary if we want to study cross-sectional variation across villages, rather than only pairwise comparisons). We report these results for each covariate separately, as well as both together (note that the latter specification makes sense as a causal model only if mask-wearing does not directly cause physical distancing).

We find clear evidence of a negative relationship between mask-wearing and both symptoms and symptomatic seropositivity. Once we control for mask-wearing, we see no significant relationship between physical distancing and symptomatic seropositivity. The standard deviation of the change in mask-wearing across villages is also considerably larger than the change in physical distancing, at 0.21 vs. 0.13 respectively, so even were the coefficients the same, the change in mask-wearing in the causal interpretation would account for more of the variation outcomes.

Figure S6: Variation of Effect within Treatment Arms

(a) Symptomatic Seroprevalence by Change in Mask Wearing

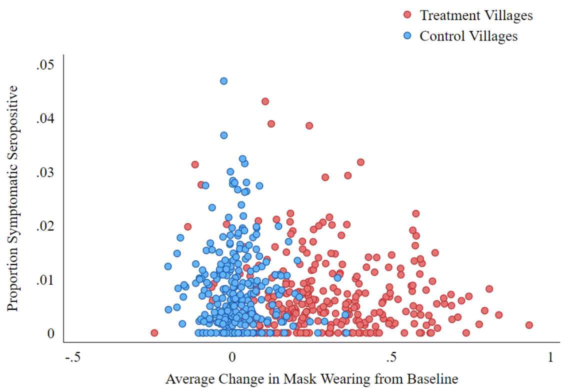

(b) Symptoms by Change in Mask Wearing

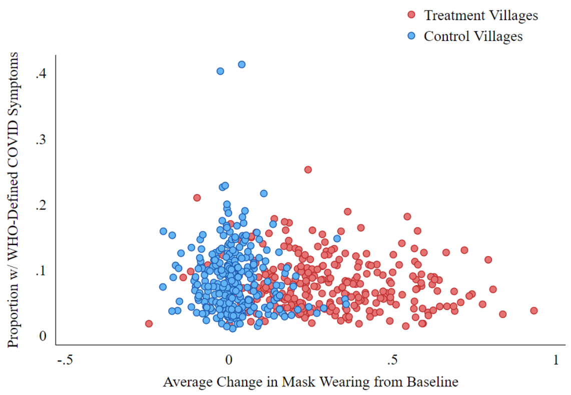

(c) Symptomatic Seroprevalence by Change in Physical Distancing

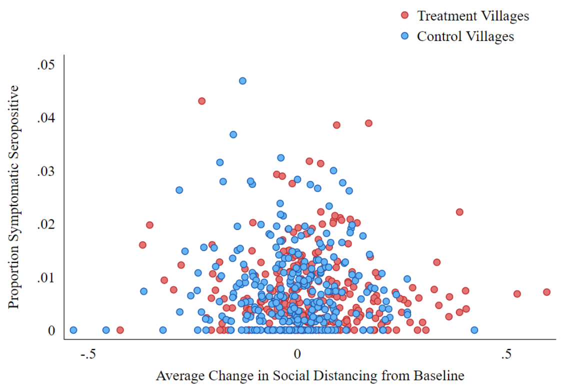

(d) Symptoms by Change in Physical Distancing

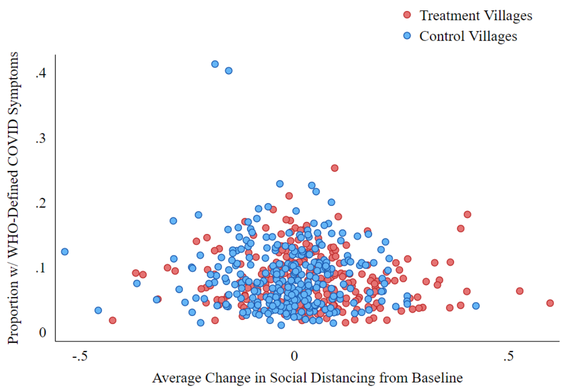

Panels S6a and S6b plot the average change in mask wearing for each village against the proportion of people in their village that are symptomatic-seropositive (panel S6a) or have WHO-defined COVID symptoms (panel S6b). Panels S6c and S6d plot for each village, the average change in physical distancing against the proportion of individuals that are symptomatic-seropositive (panel S6c) or have WHO-defined COVID symptoms (panel S6d). Change in mask wearing [physical distancing] is the difference in the average rate of mask wearing [physical distancing] during the intervention between the average rate of mask wearing [physical distancing] in the baseline. Each point represents a village, with color indicating treatment status.

Table S29: Symptomatic Seroprevalence & COVID Symptoms by Mask Wearing & Physical Distancing

	Mask Wearing	Physical Distancing	Mask Wearing & Physical Distancing
	WHO-Defined COVID Symptoms		
Coefficient on Change in Mask-Wearing from Baseline	-0.0279*** (0.0081)		-0.0241*** (0.0081)
Coefficient on Change in Social-Distancing from Baseline		-0.0301* (0.0157)	-0.0174 (0.0159)
N Individuals	321,948	321,948	321,948
N Villages	572	572	572
	Symptomatic-Seropositivity		
Coefficient on Change in Mask-Wearing from Baseline	-0.0032** (0.0013)		-0.0028** (0.0014)
Coefficient on Change in Social-Distancing from Baseline		-0.0031 (0.0023)	-0.0016 (0.0025)
N Individuals	304,726	304,726	304,726
N Villages	572	572	572

Standard errors are in parentheses.
*** Significant at the 1 percent level. ** Significant at the 5 percent level. * Significant at the 10 percent level.
All regressions include controls for baseline rates of mask-wearing and baseline symptom rates.
Baseline Symptom Rate is defined as the rate of surveyed individuals in a village who report symptoms coinciding with the WHO definition of a probable COVID-19 case. We assume that (1) all reported symptoms were acute onset, (2) all people live or work in an area with high risk of transmission of virus and (3) all people have been a contact of a probable or confirmed case of COVID-19 or are linked to a COVID-19 cluster.
The analysis in the top panel includes all people surveyed in the baseline household visits, excluding individuals that we did not collect midline or endline symptoms for.
The analysis in the bottom panel includes all people surveyed in the baseline household visits, excluding individuals that we did not collect midline or endline symptoms for, symptomatic individuals that we did not collect blood from, and individuals that we drew blood from but did not test their blood.

P Additional Preregistered Specifications

In this section, we discuss additional preregistered specifications not reported in the text. For reference, our pre-analysis plan is available at: https://osf.io/vzdh6/. Our initial intention had been to collect blood from a single high-risk individual within each household at endline. When we failed to collect as many baseline bloodspots as hoped, we decided to test all symptomatic individuals at endline rather than a single high-risk individual in each household. The only data observed at the time of this decision was the count of total baseline bloodspots collected.

We had initially planned to do only telephone surveys at weeks 5 and 9. Near the start of our Week 9 activities, we switched to in-person household surveys in Week 9 in order to increase the survey response rate. At the time this decision was made, analysts were still blinded to treatment and control villages. In total, we surveyed 104,063 households in week 5 (all phone surveys), and 118,018 households in week 9. Of these, 102,871 were household surveys.

Our pre-registration document suggests that we can compute the impact of our intervention on seroconversions by comparing our effect size to the difference between endline and baseline seropositives among individuals symptomatic during our intervention. As the analysis in Appendix I makes clear, this is not quite correct. If P_{prior}, the fraction of symptomatic seropositives due to infections prior to baseline, is zero, then the estimated impact on symptomatic seropositives equals the impact on symptomatic seroconversions and no further adjustment is needed. More generally, the impact on symptomatic seropositives incorporates both seroconversions, as well as reductions in symptomatic seroconversions due to non-COVID respiratory diseases. We cannot determine the impact on seroconversions without knowing both $P_{prior}(0)$ and the relative impact of masks on COVID-19 and non-COVID respiratory diseases. If the latter two quantities are equal in proportion, the impact on symptomatic seropositives again equals the impact on symptomatic seroconversions with no further adjustment needed.

Given that we find no evidence of an impact of any of the cross-randomizations, we did not estimate the specification flexibly interacting them.

We did not proceed with the "individual intervention" described in the pre-registration docu-

ment which was designed to test the protective benefits of masks to the wearer, because we were unable to entice a sufficient number of markets and vendors to participate in that trial and switch mask-wearing behavior.

We did not collect the intended pharmacy data to use as an auxiliary outcome, and we did not collect follow-up hospitalization and mortality data due to the expense of revisiting households. We also do not yet have data on distance to nearby city or estimated average village-wealth.

In Table S30, we report our pre-specified instrumental variable regressions. If we assume that the entire impact of our intervention is via proper mask-wearing, then we estimate that going from zero percent to one hundred percent of villagers wearing masks would reduce symptomatic seroprevalence by -0.0024, a 32% reduction. Essentially, this specification scales our “intent-to-treat” estimates by a factor of 3.33, the reciprocal of the first stage.

Table S30: IV Regressions

	Symptomatic Seroprevalence	WHO-Defined COVID-19 Symptoms
	No Baseline Controls	
Proper Mask-Wearing Coefficient	-0.0024**	-0.0327***
	(0.0012)	(0.0077)
	With Baseline Controls	
Proper Mask-Wearing Coefficient	-0.0024**	-0.0325***
	(0.0012)	(0.0075)
N Individuals	304,726	321,948
N Villages	572	572

Standard errors are in parentheses.
*** Significant at the 1 percent level. ** Significant at the 5 percent level. * Significant at the 10 percent level.
All regressions include an indicator for each control-intervention pair. The regressions "with baseline controls" include controls for baseline rates of proper mask wearing and baseline symptom rates.
Baseline Symptom Rate is defined as the rate of surveyed individuals in a village who report symptoms coinciding with the WHO definition of a probable COVID-19 case. We assume that (1) all reported symptoms were acute onset, (2) all people live or work in an area with high risk of transmission of virus and (3) all people have been a contact of a probable or confirmed case of COVID-19 or are linked to a COVID-19 cluster.
The analysis includes all people surveyed in the baseline household visits, excluding individuals that we did not collect midline or endline symptoms for.
Proper Mask-Wearing is defined as the village-level rate of individuals observed properly wearing mask during the intervention period. The instrument is the treatment status of the village.

Q Intervention Cost and Benefit Estimates

The average person-day of staff time in our intervention cost $20 of wages plus $0.50 of communication costs. All management salaries, benefits, support, internal monitoring, and equipment costs $71,696. We exclude these from the below calculation as they will vary from setting to setting. As reported in the main text, we estimate that we induced 51,660 people to regularly wear masks, or 173 people per intervention village.

Costs per village The main fixed costs of the intervention (as opposed to costs that vary over days):

- Masks for initial household distribution (3 masks per household), ($0.13 per surgical mask and $0.50 per cloth masks), 68,775 cloth masks, and 136,770 surgical masks
- Staffing for initial household distribution (4 person-days per village)
- 1 person-day of training per village
- PPE for staff: $70 per village
- Media costs: $100 per village
- Other transportation and materials costs: $30 per village

This amounts to fixed costs of: $302.50 per village for non-mask materials, $347.35 worth of cloth masks per village, and $89.35 of surgical masks per village. We estimate that we induced 598 x 29% = 173 people per village to wear masks, which amounts to fixed costs of $3.75 per adult induced to wear a mask in cloth mask villages, and $2.26 per adult in surgical mask villages.

Costs per village-day of intervention The main costs paid per day of the intervention:

- 1,089,947 masks distributed through promotion over an average of 29 days per village. Of these, there were 301,868 cloth masks distributed (105 cloth masks per day per village) and 788,079 surgical masks distributed (160 surgical masks per day per village).

- 14 person-days per week per village in week 1, 8 person-days per week per village in week 2, 6 person-days per village in weeks 3, 4 and 5, and 4 person-days per week per village thereafter.

Over the first four weeks of our intervention, this amounts to mask supply costs of $52.57 per village-day for cloth masks and $17.75 per village-day for surgical masks. The promotion costs were $24 per village-day. Dividing by the number of people induced to wear masks per village (173), we obtain costs of $0.44 per person-day in cloth mask villages and $0.24 per person-day in surgical mask villages. Using these figures, we calculated that after subtracting surveillance costs, our intervention cost $17.00 for each person induced to regularly wear a cloth mask and $9.49 for each person to regularly wear a surgical mask.

Cost-effectiveness To determine the impact of the intervention using surgical masks in reducing mortality from COVID-19 in Bangladesh, we used estimates of current and projected deaths from COVID-19, including excess deaths that occurred over the same time period (May 1, 2021-September 1, 2021) (*80*). The lower bound includes only COVID-19 reported deaths. The mid-range estimates include 50% of excess deaths as being directly attributable to COVID-19. The upper bound includes all excess deaths that occurred over the same time period as being directly attributable to COVID-19. We projected the impact of the intervention using surgical masks on deaths over four months following one month of intervention. We calculated the absolute risk reduction as the difference in death rate over the intervening period with and without the surgical mask intervention. We applied a 35% reduction of deaths among those 60 and older and a 23% reduction of deaths among those aged 50-60 based on the study findings and age-adjusted COVID-19 mortality rates for Bangladesh (*81*). We assumed no change in deaths for those under age 50. We determined the number needed to treat by taking the inverse of the absolute risk reduction.

As shown in Table S31, for one month of the intervention, the number needed to treat to prevent one death ranges from 6,682 to 35,001. Our estimates above suggest that the total cost of our intervention per person induced to wear a mask for a month was: $3.75 + $0.44 x 30 = $17.00

in cloth mask villages and $2.26 + $0.24 x 30 = $9.49 in surgical mask villages. By multiplying the number needed to treat times the cost per person induced to wear a mask, we estimate that after four months, the intervention as we conducted it (with cloth and surgical masks) cost between $63,408 and $332,161 per life saved, depending on mortality estimates. Notably, we do not assume continued mask-wearing beyond one month. Rather, infections prevented during the one month of the intervention propagate into infections prevented in future months. Furthermore, this does not account for reductions of morbidity associated with hospitalization or other complications of COVID-19.

Table S31: Calculation of Number Needed to Treat and Cost per Life Saved

	COVID-19-related Deaths (May 1 - Sept 1, 2021)*	Estimated Deaths with Intervention†	ARR	NNT	Cost per Life Saved - Intervention (USD)	Cost per Life Saved - at Scale (USD)
Lower bound	17,984	13,233	2.86E-05	35,001	$332,161	$52,502
Mid-range	56,097	41,276	8.91E-05	11,221	$106,487	$16,831
Upper bound	94,209	69,319	1.50E-04	6,682	$63,408	$10,022

ARR = Absolute Risk Reduction; NNT = Number Needed to Treat
*https://covid19.healthdata.org/bangladesh
†Applying 35% reduction to deaths in the 60+ age group and 23% reduction to deaths in the 50-59 age group

Many cost elements can be brought down further through "at-scale implementation". This is because some of our information campaigns and promotion activities had to be individualized for the purposes of conducting a trial with a control group, whereas at scale the government could use mass media and social media based dissemination strategies more cost-effectively. Additionally, surgical masks are about 8 times cheaper than cloth masks, and factory production costs can be brought down at scale. We calculate based on our current at scale activities that conducting the intervention for one month for the entire country of Bangladesh would cost $1.50 USD/person. Following out the effects for four months after one month of intervention, this translates to sub-

stantially lower costs per life saved: \$10,022-\$52,502 (Table S31).

For context, (*51*) estimate that the value of a statistical life is \$205,000 in Bangladesh, implying that our intervention at scale is 4-20 times more cost-effective than what the typical Bangladeshi would be willing to pay to reduce mortality risk, and therefore a "very good buy" for policymakers. This cost-effectiveness analysis was not pre-specified.

R Polling Policy Makers

R.1 Polling and Policy-Maker Priors

To assess how our findings compared to the priors of relevant policy makers, we polled participants during presentations to the World Health Organization, the World Bank, and the National Council of Applied Economic Research in Delhi, India. In total, more than 100 audience members with expertise and specific interest in public health and mask-wearing were surveyed and asked to make predictions about the impact of our various interventions on mask-wearing and physical distancing, just before we showed them our empirical results (at the time, our biological outcomes were unavailable).

There are three main takeaways from this polling exercise: first, only a tiny fraction of policy-makers correctly predicted the impact of our core intervention on mask-wearing and physical distancing. Second, policy maker predictions varied widely, both for effects of the intervention on mask-wearing and physical distancing. Third, policy-makers systematically underestimated the overall impact of our intervention and especially the impact of in-person reinforcement on mask-wearing.

When asked if they thought the intervention would increase mask-wearing by 5, 10, 20, 30, or 40 percentage points, only 21% of respondents correctly predicted that the intervention increased mask-wearing by 30 percentage points (about what we would expect if they guessed randomly). The expected value of the predicted increase in mask-wearing was 22 percentage points whether we described the intervention with or without mask promotion included. The difference in mask-

wearing observed in our two pilot studies suggests that in-person reinforcement increased mask-wearing by 18 percentage points. In other words, policy-makers makers believed that in-person reinforcement would have no additional impact, despite our piloting suggesting it is the single most important element of our intervention. With regard to behavioral adjustments, 64% of respondents predicted that physical distancing would either decrease or remain unchanged as a result of the mask-promotion interventions, when in fact, it increased.

Policy-makers consistently believed that our cross-randomizations would increase mask-wearing, when in fact, we find that none of them had a significant effect (often with fairly precise zeros). 68% of respondents believed that text messages would help (they didn't), 62% of respondents believed that incentives for village-leaders would help (they didn't), and 77% of respondents believed that verbal commitments or commitments made using signs on one's door would increase mask-wearing (they didn't). More results from this polling exercise are presented in the tables below.

Table S32: What do you think was the increase in mask-wearing as a result of household mask distribution and mask promotion in the community?

	WHO	NCAER	World Bank	Frequency	Percent
No change	0	1	3	4	3%
Increased by 5 percentage points	5	10	8	23	20%
Increased by 10 percentage points	4	12	8	24	21%
Increased by 20 percentage points	4	19	9	32	28%
Increased by 30 percentage points	4	7	11	22	19%
Increased by 40 percentage points	2	6	3	11	9%
Total	19	55	42	116	100%

These are polls taken in response to the prompt: "We provided free masks to all households and promoted mask-wearing in mosques and markets with community leaders and imams. What do you think happened to mask-wearing relative to the 13% proper mask usage rate in the control villages without any interventions?" The results were collected from audience participants during live presentations to the World Health Organization (WHO), the National Council of Applied Economic Research (NCAER) in Delhi, and the World Bank.

Table S33: What do you think was the additional effect of mask promoters reminding people to wear masks?

	WHO	NCAER	World Bank	Frequency	Percent
No change	0	1	4	5	4%
Increased by 5 percentage points	2	4	5	11	9%
Increased by 10 percentage points	6	20	5	31	26%
Increased by 20 percentage points	2	10	14	26	22%
Increased by 30 percentage points	4	10	11	25	21%
Increased by 40 percentage points	5	10	7	22	18%
Total	19	55	46	120	100%

These are polls taken in response to the prompt: " In addition to the mask distribution and promotion activities described previously, we had mask promoters periodically monitor passers-by and remind them to wear masks. What do you think happened to mask-wearing relative to the 13% proper mask usage rate in the control villages without any interventions?"
The results were collected from audience participants during live presentations to the World Health Organization (WHO), the National Council of Applied Economic Research (NCAER) in Delhi, and the World Bank.

Table S34: Do you think text message reminders to wear masks further increased mask-wearing?

	WHO	NCAER	World Bank	Frequency	Percent
Yes	0	33	32	65	68%
No	0	19	11	30	32%
Total	0	52	43	95	100%

These are polls taken in response to the prompt: "We sent text reminders to wear masks. Do you think this increased mask-wearing further?"
The results were collected from audience participants during live presentations to the World Health Organization (WHO), the National Council of Applied Economic Research (NCAER) in Delhi, and the World Bank.

Table S35: How do you think mask distribution and promotion affected physical distancing?

	WHO	NCAER	World Bank	Frequency	Percent
Physical distancing decreased	5	0	8	13	22%
Physical distancing was unchanged	9	0	16	25	42%
Physical distancing increased	5	0	17	22	37%
Total	19	0	41	60	100%

These are polls taken in response to the prompt: "How did mask distribution and promotion affect individuals' physical distancing?"
The results were collected from audience participants during live presentations to the World Health Organization (WHO), the National Council of Applied Economic Research (NCAER) in Delhi, and the World Bank.

Table S36: Do you think incentive payments to village leaders further increased mask-wearing?

	WHO	NCAER	World Bank	Frequency	Percent
Yes	0	32	0	32	62%
No	0	20	0	20	38%
Total	0	52	0	52	100%

These are polls taken in response to the prompt: "We promised the village and leaders an incentive payment if we saw increases in mask-wearing. Do you think this increased mask-wearing further?"
The results were collected from audience participants during live presentations to the World Health Organization (WHO), the National Council of Applied Economic Research (NCAER) in Delhi, and the World Bank.

Table S37: Do you think verbal commitments and signage to wearing masks further increased mask-wearing?

	WHO	NCAER	World Bank	Frequency	Percent
Yes	0	40	0	40	77%
No	0	12	0	12	23%
Total	0	52	0	52	100%

These are polls taken in response to the prompt: "We had households verbally committing to wear masks and putting up signs to display to others that they were a mask-wearing household. Do you think this increased mask-wearing further?"
The results were collected from audience participants during live presentations to the World Health Organization (WHO), the National Council of Applied Economic Research (NCAER) in Delhi, and the World Bank.

References and Notes

1. J. Howard, A. Huang, Z. Li, Z. Tufekci, V. Zdimal, H.-M. van der Westhuizen, A. von Delft, A. Price, L. Fridman, L.-H. Tang, V. Tang, G. L. Watson, C. E. Bax, R. Shaikh, F. Questier, D. Hernandez, L. F. Chu, C. M. Ramirez, A. W. Rimoin, An evidence review of face masks against COVID-19. *Proc. Natl. Acad. Sci. U.S.A.* **118**, e2014564118 (2021). doi:10.1073/pnas.2014564118 Medline

2. N. H. L. Leung, D. K. W. Chu, E. Y. C. Shiu, K.-H. Chan, J. J. McDevitt, B. J. P. Hau, H.-L. Yen, Y. Li, D. K. M. Ip, J. S. M. Peiris, W.-H. Seto, G. M. Leung, D. K. Milton, B. J. Cowling, Respiratory virus shedding in exhaled breath and efficacy of face masks. *Nat. Med.* **26**, 676–680 (2020). doi:10.1038/s41591-020-0843-2 Medline

3. C. R. MacIntyre, A. A. Chughtai, Facemasks for the prevention of infection in healthcare and community settings. *BMJ* **350**, h694 (2015). doi:10.1136/bmj.h694 Medline

4. H. Bundgaard, J. S. Bundgaard, D. E. T. Raaschou-Pedersen, C. von Buchwald, T. Todsen, J. B. Norsk, M. M. Pries-Heje, C. R. Vissing, P. B. Nielsen, U. C. Winsløw, K. Fogh, R. Hasselbalch, J. H. Kristensen, A. Ringgaard, M. Porsborg Andersen, N. B. Goecke, R. Trebbien, K. Skovgaard, T. Benfield, H. Ullum, C. Torp-Pedersen, K. Iversen, Effectiveness of adding a mask recommendation to other public health measures to prevent SARS-CoV-2 infection in Danish mask wearers: A randomized controlled trial. *Ann. Intern. Med.* **174**, 335–343 (2021). doi:10.7326/M20-6817 Medline

5. C. N. Ngonghala, E. Iboi, S. Eikenberry, M. Scotch, C. R. MacIntyre, M. H. Bonds, A. B. Gumel, Mathematical assessment of the impact of non-pharmaceutical interventions on curtailing the 2019 novel Coronavirus. *Math. Biosci.* **325**, 108364 (2020). doi:10.1016/j.mbs.2020.108364 Medline

6. C. T. Leffler, E. Ing, J. D. Lykins, M. C. Hogan, C. A. McKeown, A. Grzybowski, Association of country-wide coronavirus mortality with demographics, testing, lockdowns, and public wearing of masks. *Am. J. Trop. Med. Hyg.* **103**, 2400–2411 (2020). doi:10.4269/ajtmh.20-1015 Medline

7. W. Lyu, G. L. Wehby, Community use of face masks and COVID-19: Evidence from a natural experiment of state mandates in the US. *Health Aff.* **39**, 1419–1425 (2020). doi:10.1377/hlthaff.2020.00818 Medline

8. V. Chernozhukov, H. Kasahara, P. Schrimpf, Causal impact of masks, policies, behavior on early covid-19 pandemic in the U.S. *J. Econom.* **220**, 23–62 (2021). doi:10.1016/j.jeconom.2020.09.003 Medline

9. J. Abaluck, J. A. Chevalier, N. A. Christakis, H. P. Forman, E. H. Kaplan, A. Ko, S. H. Vermund, The case for universal cloth mask adoption and policies to increase supply of medical masks for health workers. SSRN 3567438 [Preprint] (2020); https://dx.doi.org/10.2139/ssrn.3567438.

10. Y. Cheng, N. Ma, C. Witt, S. Rapp, P. S. Wild, M. O. Andreae, U. Pöschl, H. Su, Face masks effectively limit the probability of SARS-CoV-2 transmission. *Science* **372**, 1439–1443 (2021). doi:10.1126/science.abg6296 Medline

11. A. Mullard, “How COVID vaccines are being divvied up around the world,” *Nature News*, 30 November 2020.

12. T. A. Ghebreyesus, WHO Director-General’s opening remarks at the media briefing on COVID-19 - 5 June 2020 (World Health Organization, 2020).

13. L. M. Brosseau, M. Sietsema, “Commentary: Masks-for-all for COVID-19 not based on sound data” (Center for Infectious Disease Research and Policy, University of Minnesota, 2020); www.cidrap.umn.edu/news-perspective/2020/04/commentary-masks-all-covid-19-not-based-sound-data.

14. M. A. Johansson, T. M. Quandelacy, S. Kada, P. V. Prasad, M. Steele, J. T. Brooks, R. B. Slayton, M. Biggerstaff, J. C. Butler, SARS-CoV-2 transmission from people without COVID-19 symptoms. *JAMA Netw. Open* **4**, e2035057 (2021). doi:10.1001/jamanetworkopen.2020.35057 Medline

15. Centers for Disease Control and Prevention (CDC), “Science brief: Community use of cloth masks to control the spread of SARS-CoV-2” (CDC, 2021).

16. J. M. Brophy, “Covid-19: Controversial trial may actually show that masks protect the wearer,” *BMJ Opinion*, 24 November 2020.

17. J. Pan, C. Harb, W. Leng, L. C. Marr, Inward and outward effectiveness of cloth masks, a surgical mask, and a face shield. *Aerosol Sci. Technol.* **55**, 718–733 (2021). doi:10.1080/02786826.2021.1890687

18. D. Kahneman, D. T. Miller, Norm theory: Comparing reality to its alternatives. *Psychol. Rev.* **93**, 136–153 (1986). doi:10.1037/0033-295X.93.2.136

19. J. Jordan, E. Yoeli, D. Rand, Don’t get it or don’t spread it: Comparing self-interested versus prosocial motivations for COVID-19 prevention behaviors. PsyArXiv [Preprint] (2020); https://doi.org/10.31234/osf.io/yuq7x.

20. R. B. Cialdini, N. J. Goldstein, Social influence: Compliance and conformity. *Annu. Rev. Psychol.* **55**, 591–621 (2004). doi:10.1146/annurev.psych.55.090902.142015 Medline

21. M. Bates, R. Glennerster, K. Gumede, E. Duflo, The price is wrong. *Field Actions Sci. Rep.* **4**, 30 (2012).

22. A. Karing, “Social signaling and childhood immunization: A field experiment in Sierra Leone,” Working paper, University of California, Berkeley (2018).

23. D. Karlan, M. McConnell, S. Mullainathan, J. Zinman, Getting to the top of mind: How reminders increase saving. *Manage. Sci.* **62**, 3393–3411 (2016). doi:10.1287/mnsc.2015.2296

24. N. J. Goldstein, R. B. Cialdini, V. Griskevicius, A room with a viewpoint: Using social norms to motivate environmental conservation in hotels. *J. Consum. Res.* **35**, 472–482 (2008). doi:10.1086/586910

25. G. Miller, A. M. Mobarak, Learning about new technologies through social networks: Experimental evidence on nontraditional stoves in Bangladesh. *Mark. Sci.* **34**, 480–499 (2014). doi:10.1287/mksc.2014.0845

26. P. Manchanda, Y. Xie, N. Youn, The role of targeted communication and contagion in product adoption. *Mark. Sci.* **27**, 961–976 (2008). doi:10.1287/mksc.1070.0354

27. C. Bicchieri, *Norms in the Wild: How to Diagnose, Measure, and Change Social Norms* (Oxford Univ. Press, 2016).

28. T. R. Bhuiyan, J. D. Hulse, S. T. Hegde, M. Akhtar, M. T. Islam, Z. H. Khan, I. I. Khan, S. Ahmed, M. M. Rashid, R. Rashid, E. S. Gurley, T. Shirin, A. I. Khan, A. S. Azman, F. Qadri, SARS-CoV-2 seroprevalence in Chattogram, Bangladesh before the Delta surge, March-June 2021. medRxiv 2021.07.16.21260611 [Preprint] (2021); https://doi.org/10.1101/2021.07.16.21260611.

29. icddr,b, "Higher covid-19 seropositivity observed among residents in Dhaka and Chattogram," 22 June 2021; www.icddrb.org/news-and-events/news?id=878.

30. Management Information System (MIS), Directorate General of Health Services (DGHS), COVID-19 dynamic dashboard for Bangladesh (2021); http://dashboard.dghs.gov.bd/webportal/pages/covid19.php [accessed 16 August 2021].

31. M. V. Murhekar, T. Bhatnagar, J. W. V. Thangaraj, V. Saravanakumar, M. S. Kumar, S. Selvaraju, K. Rade, C. P. G. Kumar, R. Sabarinathan, A. Turuk, S. Asthana, R. Balachandar, S. D. Bangar, A. K. Bansal, V. Chopra, D. Das, A. K. Deb, K. R. Devi, V. Dhikav, G. R. Dwivedi, S. M. S. Khan, M. S. Kumar, A. Laxmaiah, M. Madhukar, A. Mahapatra, C. Rangaraju, J. Turuk, R. Yadav, R. Andhalkar, K. Arunraj, D. K. Bharadwaj, P. Bharti, D. Bhattacharya, J. Bhat, A. S. Chahal, D. Chakraborty, A. Chaudhury, H. Deval, S. Dhatrak, R. Dayal, D. Elantamilan, P. Giridharan, I. Haq, R. K. Hudda, B. Jagjeevan, A. Kalliath, S. Kanungo, N. N. Krishnan, J. S. Kshatri, A. Kumar, N. Kumar, V. G. V. Kumar, G. G. J. N. Lakshmi, G. Mehta, N. K. Mishra, A. Mitra, K. Nagbhushanam, A. Nimmathota, A. R. Nirmala, A. K. Pandey, G. V. Prasad, M. A. Qurieshi, S. D. Reddy, A. Robinson, S. Sahay, R. Saxena, K. Sekar, V. K. Shukla, H. B. Singh, P. K. Singh, P. Singh, R. Singh, N. Srinivasan, D. S. Varma, A. Viramgami, V. C. Wilson, S. Yadav, S. Yadav, K. Zaman, A. Chakrabarti, A. Das, R. S. Dhaliwal, S. Dutta, R. Kant, A. M. Khan, K. Narain, S. Narasimhaiah, C. Padmapriyadarshini, K. Pandey, S. Pati, S. Patil, H. Rajkumar, T. Ramarao, Y. K. Sharma, S. Singh, S. Panda, D. C. S. Reddy, B. Bhargava, ICMR Serosurveillance Group, SARS-CoV-2 seroprevalence among the general population and healthcare workers in India, December 2020-January 2021. *Int. J. Infect. Dis.* **108**, 145–155 (2021). doi:10.1016/j.ijid.2021.05.040 Medline

32. A. Anand, J. Sandefur, A. Subramanian, "Three new estimates of India's all-cause excess mortality during the COVID-19 pandemic," Working paper no. 589, Center for Global Development, July 2021.

33. J. Abaluck, A. M. Mobarak, "Getting all Bangladeshis to wear masks," *WhiteBoard Magazine*, 1 December 2020.

34. A. Jakubowski, D. Egger, C. Nekesa, L. Lowe, M. Walker, E. Miguel, Self-reported mask wearing greatly exceeds directly observed use: Urgent need for policy intervention in Kenya. medRxiv 2021.01.27.21250487 [Preprint] (2021); https://doi.org/10.1101/2021.01.27.21250487.

35. K. K. Tithila, "Brac's efforts to mask up Bangladesh could be game-changer," *Dhaka Tribune* (Bangladesh), 15 July 2021.

36. S. Riaz, "Punjab authorities kick off 'NORM' campaign to increase mask-wearing," *Arab New* (Pakistan), 30 June 2021.

37. S. Bhattacharjee, "Covid-19 Crisis: India draws lessons from Bangladesh's mask study," *Business Standard* (India), 15 May 2021.

38. Republica, "Nepal Mask Campaign launches with the slogan 'Let's wear masks, let's save each other's lives'," *myRepública*, 16 August 2021).

39. G. J. Hollands, P. Carter, S. Answer, S. E. King, S. A. Jebb, D. Ogilvie, I. Shemilt, J. P. T. Higgins, T. M. Marteau, Altering the availability or proximity of food, alcohol, and tobacco products to change their selection and consumption. *Cochrane Database Syst. Rev.* **2019**, CD012576 (2019). doi:10.1002/14651858.CD012573.pub3 Medline

40. S. Naikoba, A. Hayward, The effectiveness of interventions aimed at increasing handwashing in healthcare workers – a systematic review. *J. Hosp. Infect.* **47**, 173–180 (2001). doi:10.1053/jhin.2000.0882 Medline

41. C. Houghton, P. Meskell, H. Delaney, M. Smalle, C. Glenton, A. Booth, X. H. S. Chan, D. Devane, L. M. Biesty, Barriers and facilitators to healthcare workers' adherence with infection prevention and control (IPC) guidelines for respiratory infectious diseases: A rapid qualitative evidence synthesis. *Cochrane Database Syst. Rev.* **4**, CD013582 (2020). doi:10.1002/14651858.CD013582 Medline

42. H. J. Seo, K.-Y. Sohng, S. O. Chang, S. K. Chaung, J. S. Won, M.-J. Choi, Interventions to improve hand hygiene compliance in emergency departments: A systematic review. *J. Hosp. Infect.* **102**, 394–406 (2019). doi:10.1016/j.jhin.2019.03.013 Medline

43. D. Biswas, M. Ahmed, K. Roguski, P. K. Ghosh, S. Parveen, F. A. Nizame, M. Z. Rahman, F. Chowdhury, M. Rahman, S. P. Luby, K. Sturm-Ramirez, A. D. Iuliano, Effectiveness of a behavior change intervention with hand sanitizer use and respiratory hygiene in reducing laboratory-confirmed influenza among schoolchildren in Bangladesh: A cluster randomized controlled trial. *Am. J. Trop. Med. Hyg.* **101**, 1446–1455 (2019). doi:10.4269/ajtmh.19-0376 Medline

44. S. L. McGuinness, S. F. Barker, J. O'Toole, A. C. Cheng, A. B. Forbes, M. Sinclair, K. Leder, Effect of hygiene interventions on acute respiratory infections in childcare, school and domestic settings in low- and middle-income countries: A systematic review. *Trop. Med. Int. Health* **23**, 816–833 (2018). doi:10.1111/tmi.13080 Medline

45. R. Guiteras, J. Levinsohn, A. M. Mobarak, Sanitation subsidies. Encouraging sanitation investment in the developing world: A cluster-randomized trial. *Science* **348**, 903–906 (2015). doi:10.1126/science.aaa0491 Medline

46. S. R. Patil, B. F. Arnold, A. L. Salvatore, B. Briceno, S. Ganguly, J. M. Colford Jr., P. J. Gertler, The effect of India's total sanitation campaign on defecation behaviors and child health in rural Madhya Pradesh: A cluster randomized controlled trial. *PLOS Med.* **11**, e1001709 (2014). doi:10.1371/journal.pmed.1001709 Medline

47. J. S. Solís Arce, S. S. Warren, N. F. Meriggi, A. Scacco, N. McMurry, M. Voors, G. Syunyaev, A. A. Malik, S. Aboutajdine, O. Adeojo, D. Anigo, A. Armand, S. Asad, M. Atyera, B. Augsburg, M. Awasthi, G. E. Ayesiga, A. Bancalari, M. Björkman Nyqvist, E. Borisova, C. M. Bosancianu, M. R. Cabra García, A. Cheema, E. Collins, F. Cuccaro, A.

Z. Farooqi, T. Fatima, M. Fracchia, M. L. Galindo Soria, A. Guariso, A. Hasanain, S. Jaramillo, S. Kallon, A. Kamwesigye, A. Kharel, S. Kreps, M. Levine, R. Littman, M. Malik, G. Manirabaruta, J. L. H. Mfura, F. Momoh, A. Mucauque, I. Mussa, J. A. Nsabimana, I. Obara, M. J. Otálora, B. W. Ouédraogo, T. B. Pare, M. R. Platas, L. Polanco, J. A. Qureshi, M. Raheem, V. Ramakrishna, I. Rendrá, T. Shah, S. E. Shaked, J. N. Shapiro, J. Svensson, A. Tariq, A. M. Tchibozo, H. A. Tiwana, B. Trivedi, C. Vernot, P. C. Vicente, L. B. Weissinger, B. Zafar, B. Zhang, D. Karlan, M. Callen, M. Teachout, M. Humphreys, A. M. Mobarak, S. B. Omer, COVID-19 vaccine acceptance and hesitancy in low- and middle-income countries. *Nat. Med.* **27**, 1385–1394 (2021). doi:10.1038/s41591-021-01454-y Medline

48. Y. Yan, J. Bayham, A. Richter, E. P. Fenichel, Risk compensation and face mask mandates during the COVID-19 pandemic. *Sci. Rep.* **11**, 3174 (2021). doi:10.1038/s41598-021-82574-w Medline

49. A. Cohen, L. Einav, Estimating risk preferences from deductible choice. *Am. Econ. Rev.* **97**, 745–788 (2007). doi:10.1257/aer.97.3.745

50. M. L. Kasting, G. K. Shapiro, Z. Rosberger, J. A. Kahn, G. D. Zimet, Tempest in a teapot: A systematic review of HPV vaccination and risk compensation research. *Hum. Vaccin. Immunother.* **12**, 1435–1450 (2016). doi:10.1080/21645515.2016.1141158 Medline

51. W. K. Viscusi, C. J. Masterman, Income elasticities and global values of a statistical life. *J. Benefit Cost Anal.* **8**, 226–250 (2017). doi:10.1017/bca.2017.12

52. GiveWell, 2021 GiveWell cost-effectiveness analysis – Version 1 (2021); www.givewell.org/how-we-work/our-criteria/cost-effectiveness/cost-effectiveness-models/changelog-2021#Version_1_Published_May_5_2021 [accessed 4 January 2021].

53. J. Hadfield, C. Megill, S. M. Bell, J. Huddleston, B. Potter, C. Callender, P. Sagulenko, T. Bedford, R. A. Neher, Nextstrain: Real-time tracking of pathogen evolution. *Bioinformatics* **34**, 4121–4123 (2018). doi:10.1093/bioinformatics/bty407 Medline

54. L. H. Kwong, R. Wilson, S. Kumar, Y. S. Crider, Y. Reyes Sanchez, D. Rempel, A. Pillarisetti, Review of the breathability and filtration efficiency of common household materials for face masks. *ACS Nano* **15**, 5904–5924 (2021). doi:10.1021/acsnano.0c10146 Medline

55. S. Duncan, P. Bodurtha, S. Naqvi, The protective performance of reusable cloth face masks, disposable procedure masks, KN95 masks and N95 respirators: Filtration and total inward leakage. *PLOS ONE* **16**, e0258191 (2021). doi:10.1371/journal.pone.0258191 Medline

56. O. O. Fadare, E. D. Okoffo, Covid-19 face masks: A potential source of microplastic fibers in the environment. *Sci. Total Environ.* **737**, 140279 (2020). doi:10.1016/j.scitotenv.2020.140279 Medline

57. Environment and Social Development Organization (ESDO), "COVID-19 pandemic pushes single use plastic waste outbreak: No management, no protection: High health and environmental risk unveil" (ESDO, 2020).

58. I. M. Steensgaard, K. Syberg, S. Rist, N. B. Hartmann, A. Boldrin, S. F. Hansen, From macro- to microplastics – Analysis of EU regulation along the life cycle of plastic bags. *Environ. Pollut.* **224**, 289–299 (2017). doi:10.1016/j.envpol.2017.02.007 Medline

59. E.-S. Jang, C.-W. Kang, Do face masks become worthless after only one use in the COVID-19 pandemic? *Infect. Chemother.* **52**, 583–591 (2020). doi:10.3947/ic.2020.52.4.583 Medline

60. M. Kremer, E. Miguel, The illusion of sustainability. *Q. J. Econ.* **122**, 1007–1065 (2007). doi:10.1162/qjec.122.3.1007

61. P. S. van Eck, W. Jager, P. S. Leeflang, Opinion leaders' role in innovation diffusion: A simulation study. *J. Prod. Innov. Manage.* **28**, 187–203 (2011). doi:10.1111/j.1540-5885.2011.00791.x

62. E. Oster, R. Thornton, Determinants of technology adoption: Peer effects in menstrual cup take-up. *J. Eur. Econ. Assoc.* **10**, 1263–1293 (2012). doi:10.1111/j.1542-4774.2012.01090.x

63. H. Allcott, Social norms and energy conservation. *J. Public Econ.* **95**, 1082–1095 (2011). doi:10.1016/j.jpubeco.2011.03.003

64. R. Guiteras, J. Levinsohn, A. M. Mobarak, "Demand estimation with strategic complementarities: Sanitation in Bangladesh," Discussion paper no. DP13498, Centre for Economic Policy Research, January 2019).

65. L. Beaman, A. BenYishay, J. Magruder, A. M. Mobarak, Can network theory-based targeting increase technology adoption? *Am. Econ. Rev.* **111**, 1918–1943 (2021). doi:10.1257/aer.20200295

66. N. Ashraf, O. Bandiera, K. Jack, No margin, no mission? A field experiment on incentives for public service delivery. *J. Public Econ.* **120**, 1–17 (2014). doi:10.1016/j.jpubeco.2014.06.014

67. R. Chetty, E. Saez, L. Sandor, What policies increase prosocial behavior? An experiment with referees at the *Journal of Public Economics*. *J. Econ. Perspect.* **28**, 169–188 (2014). doi:10.1257/jep.28.3.169

68. D. Ariely, A. Bracha, S. Meier, Doing good or doing well? Image motivation and monetary incentives in behaving prosocially. *Am. Econ. Rev.* **99**, 544–555 (2009). doi:10.1257/aer.99.1.544

69. G. Bryan, D. Karlan, S. Nelson, Commitment devices. *Annu. Rev. Econ.* **2**, 671–698 (2010). doi:10.1146/annurev.economics.102308.124324

70. J. Luoto, D. Levine, J. Albert, S. Luby, Nudging to use: Achieving safe water behaviors in Kenya and Bangladesh. *J. Dev. Econ.* **110**, 13–21 (2014). doi:10.1016/j.jdeveco.2014.02.010

71. N. Ashraf, O. Bandiera, E. Davenport, S. S. Lee, Losing prosociality in the quest for talent? Sorting, selection, and productivity in the delivery of public services. *Am. Econ. Rev.* **110**, 1355–1394 (2020). doi:10.1257/aer.20180326

72. L. Bursztyn, R. Jensen, Social image and economic behavior in the field: Identifying, understanding, and shaping social pressure. *Annu. Rev. Econ.* **9**, 131–153 (2017). doi:10.1146/annurev-economics-063016-103625

73. World Health Organization (WHO), WHO COVID-19 case definition (2020); www.who.int/publications/i/item/WHO-2019-nCoV-Surveillance_Case_Definition-2020.2 [accessed on 15 October 2020].

74. P. Guimarães, P. Portugal, A simple feasible procedure to fit models with high-dimensional fixed effects. *Stata J.* **10**, 628–649 (2010). doi:10.1177/1536867X1101000406

75. S. Gaure, "OLS with multiple high dimensional category dummies," Memorandum 14/2010, Oslo University (2011).

76. P. Guimarães, P. Portugal, A simple feasible procedure to fit models with high-dimensional fixed effects. *Stata J.* **10**, 628–649 (2011). doi:10.1177/1536867X1101000406

77. G. Zou, A modified poisson regression approach to prospective studies with binary data. *Am. J. Epidemiol.* **159**, 702–706 (2004). doi:10.1093/aje/kwh090 Medline

78. J. Abaluck, L. H. Kwong, A. Styczynski, A. Haque, M. A. Kabir, E. Bates-Jefferys, E. Crawford, J. Benjamin-Chung, S. Raihan, S. Rahman, S. Benhachmi, N. Z. Bintee, P. J. Winch, M. Hossain, H. M. Reza, A. A. Jaber, S. Gulshan Momen, A. Rahman, F. L. Banti, T. S. Huq, S. P. Luby, A. M. Mobarak, Impact of community masking on COVID-19: A cluster-randomized trial in Bangladesh. Zenodo (2021); https://doi.org/10.5281/zenodo.5703876.

79. C. Rutterford, A. Copas, S. Eldridge, Methods for sample size determination in cluster randomized trials. *Int. J. Epidemiol.* **44**, 1051–1067 (2015). doi:10.1093/ije/dyv113 Medline

80. Institute for Health Metrics and Evaluation (IHME), COVID-19 projections – Bangladesh (2021); https://covid19.healthdata.org/bangladesh?view=cumulative-deaths&tab=trend [accessed 17 August 2021].

81. WHO Bangladesh, COVID-19 Morbidity and Mortality Weekly Update, vol. 76, 9 August 2021; https://cdn.who.int/media/docs/default-source/searo/bangladesh/covid-19-who-bangladesh-situation-reports/who_covid-19-update_76_20210809.pdf?sfvrsn=9208a5a_9.
